# Cluster size convergence for the energetics of the oxygen evolving complex in PSII

**DOI:** 10.1002/jcc.24863

**Published:** 2017-06-30

**Authors:** Per E. M. Siegbahn, Xichen Li

**Affiliations:** ^1^ Department of Organic Chemistry, Arrhenius Laboratory Stockholm University SE‐106 91 Stockholm Sweden; ^2^ College of Chemistry Beijing Normal University Beijing 100875 China

**Keywords:** water oxidation, density functional theory, cluster model, size convergence, accuracy

## Abstract

Density functional theory calculations have been made to investigate the stability of the energetics for the oxygen evolving complex of photosystem II. Results published elsewhere have given excellent agreement with experiments for both energetics and structures, where many of the experimental results were obtained several years after the calculations were done. The computational results were obtained after a careful extension from small models to a size of about 200 atoms, where stability of the results was demonstrated. However, recently results were published by Isobe et al., suggesting that very different results could be obtained if the model was extended from 200 to 340 atoms. The present study aims at understanding where this difference comes from. © 2017 The Authors. Journal of Computational Chemistry Published by Wiley Periodicals, Inc.

## Introduction

Water oxidation in photosystem II (PSII) occurs by the use of sunlight in four steps, with intermediates termed S_0_ to S_4_. The catalyst is an Mn_4_Ca complex, termed the oxygen evolving complex (OEC), where the metals are bridged by oxo bonds. During the past decade, continuous efforts have been made trying to model water oxidation in PSII using theoretical models. Computational modeling without a starting X‐ray structure is very difficult and only qualitative results could be obtained before 2004. The initial quantitative modeling of water oxidation started from low‐resolution X‐ray structures with rather large uncertainties.^1^, ^2^ Nevertheless, in 2006 a mechanism was suggested in which the O—O bond of O_2_ is formed in S_4_ between an oxygen radical (oxyl) group and a bridging oxo group.^3^ That type of mechanism was found to be much superior to all other mechanisms investigated, for example, to a water attack on an oxo‐ (or oxyl‐) ligand, a finding confirmed also quite recently.^4^ Two years later, an improved structure of the OEC was also suggested.^5^ This structure was confirmed by high‐resolution X‐ray spectroscopy in 2011.^6^ Details of the structures of the S_1_ to S_3_ intermediates, have during 2011–2014 been confirmed in detail by spectroscopy.^7^

**Figure 1:**
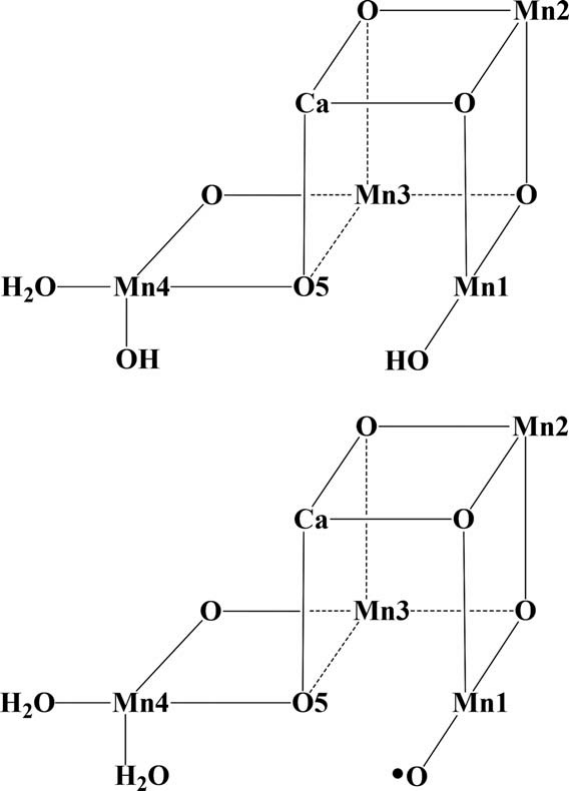
The two different S_3_ states discussed here; above with an OH on Mn1, below with an oxyl radical on Mn1. The upper structure has been strongly favored in previous studies, while the recent study by Isobe et al. place these structures at the same energy.

During the past decade, models of different sizes have been used for modeling the mechanism, the largest one being composed of around 200 atoms.^8^ Good convergence of the energetics with model size were obtained. Therefore, it came as a big surprise when quite different energetic results were obtained recently by Isobe et al. using a much larger model with 340 atoms.^9^ Different electronic states of the S_3_ intermediate were modeled. In particular, they found that an S_3_ state with an oxygen radical had a very low energy compared to the ground state found earlier. In that structure, the proton was moved to the OH‐group on the outer manganese (Mn4), from the OH group on Mn1, see Figure 1. Importantly, the two states have the same charge. Using the same computational level as the one used earlier in the previous study with 200 atoms, they found the oxygen radical state to be the ground state by −0.4 kcal/mol. Using the previous model, the oxygen radical state was here found to be at +21.3 kcal/mol. The difference to the old results was claimed to be due to the use of a larger model with, for example, a large number of water molecules not included before, with positions taken from the high‐resolution X‐ray structure. In the present study, the convergence of this energy difference has been investigated using models of different sizes between 202 and 340 atoms.

## Methods and Models

The density functional theory (DFT) calculations discussed here were performed in essentially the same way as described in detail previously.^8^ The hybrid functional B3LYP*^10^, ^11^ was used with polarized basis sets for the geometries (lacvp*), large basis sets for energies (cc‐pvtz(‐f)/lacv3p+), and a surrounding dielectric medium with dielectric constant equal to 6.0 (basis lacvp*). Dispersion effects were added using the empirical D2 formula of Grimme.^12^ Full geometry optimizations (except for fixed backbone atoms) were made for all structures. For the 202 atom model, the same back‐bone atoms were fixed as in the previous study.^8^ This means fixing the alpha‐carbon and the two neighboring hydrogen atoms placed along the X‐ray backbone. For the larger cluster, the added atoms were placed in the following way to simplify the incorporation of them, while keeping the same atoms fixed for the 202 atom part of the cluster. The metal atoms of the OEC were first overlapped with the ones of the 340 atom structure of Isobe et al. as well as possible. The added atoms were after that taken directly from that 340 atom structure.^9^ For these atoms, only the alpha‐carbons were fixed in most cases. For Val185, an additional hydrogen was fixed, and also for Asn298. For all structures, it was visually checked that no major distortions were found during the geometry optimizations. It should be added that experience has shown that the exact positions of the fixed atoms are not important for relative energies as long as the fixing is identical for the structures that are compared. When this is fulfilled even rather large changes in their positions have only very small effects on relative energies.^13^ The coordinates and the fixing of the atoms are shown in the Supporting Information. The calculations were performed with the Jaguar and Gaussian programs.^14^, ^15^

## Results

In the present study, the convergence of a certain energy difference for the OEC is investigated starting from the cluster model used in our previous study of 202 atoms,^8^ and ending at a model size of 340 atoms used by Isobe et al.^9^ This energy difference concerns the one between the ground state for the S_3_ state of the OEC and another state of S_3_ with an oxygen radical instead of an OH group bound to Mn1. The core structures of the two states are sketched in Figure 1. For the 202 atom model, the energy difference is +21.3 kcal/mol if the model is used in exactly the same form as previously, entry 202(a) in Table 1. The energy difference reported by Isobe et al. for the 340 atom model is very different with −0.4 kcal/mol. This dramatic change of an energy difference by extending the model from 202 to 340 atoms is completely different from what would have been expected based on the experience obtained during at least a decade of model tests. It should be added that the convergence of the cluster that led to the 202 atom model was tested for other energy differences than the one studied here. This convergence was tested by, for example, varying the number of external water molecules and their positions. Of the two states in Figure 1, only the hydroxyl state was studied in detail previously. If a similar convergence study is done here for the 202 atom model of the oxyl state, by moving the water molecules around, a better position is obtained for one of the external waters for that state, reducing the energy difference between the two states to 15.4 kcal/mol, entry 202(b) in Table 1. If the previous study would have been concerned with this particular energy difference, this experience would have been enough to motivate a larger cluster model than 202 atoms, including the optimal water positions for both the oxyl and the OH state at the same time with one model.

**Table 1:** Energy difference (kcal/mol) between the hydroxide and oxyl radical structures for the S_3_‐state.

Number of atoms	Energy difference
202(a)	+21.3
202(b)	+15.4
247	+16.3
261	+15.3
304	+16.3
309	+17.1
340	+16.2

A positive value means that the hydroxide state is lower in energy.

The big difference between the results obtained by Isobe et al. and the previous study, was claimed to be due to the addition of many external water molecules forming an important network. For this reason, the next model studied was extended from the 202 atom model by including all the 15 additional water molecules placed in the positions as used by Isobe et al., leading to a model of 247 atoms. However, this did not change the energy difference very much from the 202 atom model with 15.4 kcal/mol to 16.3 kcal/mol. This was expected based on previous experience. A notable advantage with the larger model is that both structures for the S_3_ states are well‐described by the same arrangement of water molecules. For the 202 atom model one water had to be moved to a different position for the oxyl state. It should be noted that several orientations of the other water molecules obviously did change between the two states.

Rather recently, it has been discovered that Val185, situated a bit outside the OEC, is important for water transport.^16^ Therefore, this group was added in the next model, leading to 261 atoms. Some of the water molecules in the Val185 region were then displaced during the optimization. However, the inclusion of Val185 did not change the energy difference very much, from 16.3 for the 247 atom model to 15.3 kcal/mol. By chance, the energy difference is now almost identical to the one for the 202 atom model. The very small differences between these first three models are within the uncertainty of the calculations, so no chemical effect is so far seen.

It is well‐known since long, that Tyr161 (Tyr_*Z*_) is very important for the mechanism of water oxidation. The main effect is that an electron is transferred to P_680_ in the reaction center, where the charge separation takes place. In this procees, Tyr_*Z*_ loses its OH proton to the nearby His191. The positive His191 is stabilized by hydrogen bonding to Asn298 residue. The Tyr_*Z*_ radical is after this ready to accept an electron from the OEC. In the next model, these three residues were therefore added leading to a 304 atom model. As the present modeling is for the ground state of S_3_, Tyr_*Z*_ is in its neutral form with an OH group. The energy difference is still very stable with 16.3 kcal/mol, see Table 1, which is also an expected result.

Again, it was decided to look at the importance of Val185 by deleting it and instead add Asn181 and the backbone part of Phe182, just like Isobe et al. did. A problem with this model is that Phe182, with just one backbone atom frozen from the X‐ray structure, can rotate and obtain a position not allowed by the rest of the X‐ray structure. During the optimization, this nonallowed rotation was therefore checked carefully, and found not to occur. The model now contains 309 atoms, as seen in the table, the energy difference between the two states is still hardly affected with 17.1 kcal/mol compared to 16.3 kcal/mol for the 304 atom model. With the two different tests of the importance of Val185, it is now clear that it is of no significance for the present energy difference.

The final model tested here is the same one as used by Isobe et al. with 340 atoms. From the 304 atom model, Gln165, Ser169, and Asn181 were added. The energy difference is still very stable with 16.2 kcal/mol compared to 16.3 kcal/mol for the 304 atom model.

It would, of course, be desirable to identify exactly where the present calculations differ from the ones by Isobe et al. However, for such a large model this is very difficult. Still, some comments can be made. A likely reason for the discrepancy to the present results is that the structure obtained by Isobe et al. has become stuck in one of a huge number of local minima present in such a big model. The same problem will occur for any large model, also of the QM/MM type, which therefore normally requires a sampling and averaging procedure. Using the cluster modeling technique, a different way to obtain stable results is to slowly extend the model as has been done here. In a first comparison between their structures and the present ones, it can be noted that the core structures of the OEC are nearly identical. The core structure includes Mn_4_Ca and the bridging oxo groups, as well as the position of the key OH or oxyl group. An indication of a problem with local minima in their optimizations, is that they did not obtain the ground state of Tyr_*Z*_ with an OH group, but instead a TyrO^–^anion. There is an energy gain going to the proper Tyr_*Z*_ state, but it is not big enough to alone explain the discrepancy of their results to the present ones. Furthermore, when the Isobe et al. structures were optimized using the present methodology starting with their structures, an energy difference of +9.8 kcal/mol was obtained compared to −0.4 kcal/mol in their study. A larger basis set has been used here for the final energy calculation, and compared to the result with the smaller basis set used for the geometry optimization, there is a large effect of nearly +10 kcal/mol. However, Isobe et al. used a basis set size somewhere in between these two basis sets, so this is not the full explanation for the discrepancy.

As a final attempt to shed light on the discrepancies of the two studies, ours and the one by Isobe et al., calculations were done by deleting atoms from the Isobe 340 atom structure. Two structures were done, one with 206 atoms and one with 309 atoms. The 206 atom structure is then similar to the 202(a) structure in Table 1. The result for the energy difference is 19.8 kcal/mol for the 206 atom model and 14.6 kcal/mol for the 309 atom model, very similar to the results of our model and quite different from the results of the Isobe 340 atom model.

## Summary

In the present study, the cluster size convergence for an energy difference between two S_3_ states has been investigated. The background is that Isobe et al. recently claimed a huge energetic effect by extending the model from the previous size with 202 atoms to 340 atoms. The previous model with 202 atoms gave an energy difference of +21.3 kcal/mol, which could here be corrected to +15.4 kcal/mol, see above. The result by Isobe et al. for their 340 atom model was −0.4 kcal/mol. To investigate this discrepancy, the energy difference between the two states was here calculated, increasing the model size from 202 atoms for six models of different sizes, at the end reaching the same size as the one used by Isobe et al. of 340 atoms. The results in Table 1 show a remarkable stability for this energy difference varying only between +15.4 to +17.1 kcal/mol. Based on previous experience a stability was expected, but an energy variation of a few kcal/mol could, of course, occur, like that seen in the table. One likely contributing factor for the large discrepancy to the results of Isobe et al. is that their geometry optimization has been stuck in a local minimum. Methodological differences, like the use of different basis sets, probably also contribute. It can be added that a very similar discrepancy to results from the Isobe group as discussed here has also very recently been pointed out for a biomimetic model of the OEC.^17^

The conclusion from the present work, and from others performed during the past two decades, is that the only way a result for a large cluster model can be trusted is that some sort of cluster size convergence test is provided. This is particularly true when the result differs strongly from previously published data. For results based on QM/MM modeling, the same problem as the one pointed out here obviously also occurs. The strategy developed in that case is to do sampling and averaging. That procedure requires calculations on a very large number of structures and can therefore not yet be adopted for cluster model calculations.

## Supporting information

Supporting Information

## References

[1] K. N. Ferreira , T. M. Iverson , K. Maghlaoui , J. Barber , S. Iwata , Science 2004, 303, 1831. 14764885 10.1126/science.1093087

[2] B. Loll , J. Kern , W. Saenger , A. Zouni , J. Biesiadka , Nature 2005, 438, 1040. 16355230 10.1038/nature04224

[3] P. E. M. Siegbahn , Chem. Eur. J. 2006, 12, 9217. 17029313 10.1002/chem.200600774

[4] P. E. M. Siegbahn , Proc. Natl. Acad. Sci USA 2017, 114, 4966. 28438997 10.1073/pnas.1617843114 PMC5441723

[5] P. E. M. Siegbahn , Chem. Eur. J. 2008, 27, 8290. 10.1002/chem.200800445 18680116

[6] (a) Y. Umena , K. Kawakami , J.‐R. Shen , N. Kamiya , Nature 2011, 473, 55; 21499260 10.1038/nature09913

[7] (a) W. Ames , D. A. Pantazis , V. Krewald , N. Cox , J. Messinger , W. Lubitz , F. Neese , J. Am. Chem. Soc. 2011, 133, 19743; 22092013 10.1021/ja2041805

[8] P. E. M. Siegbahn , Biochim. Biophys. Acta 2013, 1827, 1003. 23103385 10.1016/j.bbabio.2012.10.006

[9] H. Isobe , M. Shoji , J.‐R. Shen , K. Yamaguchi , Inorg. Chem. 2016, 55, 502. 26717045 10.1021/acs.inorgchem.5b02471

[10] A. D. Becke , J. Chem. Phys. 1993, 98, 5648.

[11] M. Reiher , O. Salomon , B. A. Hess , Theor. Chem. Acc. 2001, 107, 48.

[12] (a) S. Grimme , J. Chem. Phys. 2006, 1, 034108; 16438568 10.1063/1.2148954

[13] M. R. A. Blomberg , T. Borowski , F. Himo , R.‐Z. Liao , P. E. M. Siegbahn , Chem. Rev. 2014, 114, 3601. 24410477 10.1021/cr400388t

[14] (a) Schrodinger, Jaguar, Version 8.9, Schrodinger, Inc: New York, NY, 2015;

[15] M. J. Frisch , G. W. Trucks , H. B. Schlegel , G. E. Scuseria , M. A. Robb , J. R. Cheeseman , G. Scalmani , V. Barone , B. Mennucci , G. A. Petersson , H. Nakatsuji , M. Caricato , X. Li , H. P. Hratchian , A. F. Izmaylov , J. Bloino , G. Zheng , J. L. Sonnenberg , M. Hada , M. Ehara , K. Toyota , R. Fukuda , J. Hasegawa , M. Ishida , T. Nakajima , Y. Honda , O. Kitao , H. Nakai , T. Vreven , J. A. Montgomery, Jr ., J. E. Peralta , F. Ogliaro , M. Bearpark , J. J. Heyd , E. Brothers , K. N. Kudin , V. N. Staroverov , R. Kobayashi , J. Normand , K. Raghavachari , A. Rendell , J. C. Burant , S. S. Iyengar , J. Tomasi , M. Cossi , N. Rega , M. J. Millam , M. Klene , J. E. Knox , J. B. Cross , V. Bakken , C. Adamo , J. Jaramillo , R. Gomperts , R. E. Stratmann , O. Yazyev , A. J. Austin , R. Cammi , C. Pomelli , J. W. Ochterski , R. L. Martin , K. Morokuma , V. G. Zakrzewski , G. A. Voth , P. Salvador , J. J. Dannenberg , S. Dapprich , A. D. Daniels , Ö. Farkas , J. B. Foresman , J. V. Ortiz , J. Cioslowski , D. J. Fox , Gaussian 09, Revision C.01; Gaussian, Inc: Wallingford, CT, 2009.

[16] P. L. Dilbeck , H. Bao , C. L. Neveu , L. Burnap , Biochemistry 2013, 52, 6824. 24010490 10.1021/bi400930g

[17] S. Paul , N. Cox , D. A. Pantazis , Inorg. Chem. 2017, 56, 3875. 28291351 10.1021/acs.inorgchem.6b02777

