# Supplementary material for: Cluster size convergence for the energetics of the oxygen evolving complex in PSII
Source: J Comput Chem. 2017 Jun 30;38(25):2157–60. doi: 10.1002/jcc.24863 (PMC5575486; doi:10.1002/jcc.24863)
Supplement: Supplementary file 1 — Supporting Information [file JCC-38-2157-s001.docx]

**Supporting information for:**

Cluster size convergence for the energetics of the oxygen

 evolving complex in PSII

Per E. M. Siegbahn

Department of Organic Chemistry, Arrhenius Laboratory, Stockholm

University, SE-106 91, Stockholm, Sweden.

Xichen Li

College of Chemistry, Beijing Normal University, 100875, Beijing,

China.

**The different structures discussed in the paper**.

Previous 202 atom structure with an OH

 Mn1             3.0010807524            -1.4108789220            3.8707072036

 Mn2             4.5813484604            -1.6266285147            8.8026270460

 Mn3             5.3354245509            -1.5129034121            5.3319233042

 Mn4             6.5355214360            -0.0849577392            7.4335517495

 O5              3.5692503246            -1.1181151084            5.5971322633

 O6              5.7613657604             0.1955836927            5.8412527816

 O7              4.9959835623             0.1417488420            8.3862114347

 O8              5.9128589978            -1.8668368103            7.3786006247

 Ca9             3.4532021049             1.2201103917            6.7917884734

 C10             0.6675457306#            1.9515094407#           1.7063472934#

 H11             1.2251836376#            1.9058025727#           0.7825777531#

 H12            -0.3243897951#            2.3686415158#           1.6143241306#

 H13             0.4729246114             0.8953780233            1.9396661068

 C14             1.4794308567             2.5261391773            2.8790264076

 H15             0.8055866141             2.9054848912            3.6585141401

 H16             2.0896476200             3.3893606057            2.5863104723

 C17             2.4232495973             1.5756473894            3.6487918127

 O18             3.0707177974             2.0445945061            4.6062591255

 O19             2.4672044710             0.3450566474            3.2576662285

 C20             1.0582596502#            1.1829965126#          13.4563134137#

 H21             0.3702239488#            0.3601390381#          13.5824629694#

 H22             0.7336468391#            2.1291270287#          13.8635788046#

 H23             1.9417636345             0.9058067643           14.0510353037

 C24             1.4711178433             1.2305315699           11.9831957010

 H25             2.3428841885             1.8826685390           11.8436140346

 H26             0.6670821759             1.6287279413           11.3535855060

 C27             1.8140696840            -0.2027552479           11.5465910229

 H28             0.8891931505            -0.7890600623           11.4423030021

 H29             2.4158742481            -0.7007067027           12.3131563434

 C30             2.5548871358            -0.2961599398           10.2320475400

 O31             2.2582413906             0.4080347711            9.2593181982

 O32             3.5209077969            -1.1856832560           10.2715229893

 C33             2.7844498162#           -7.8608084299#           8.6257440817#

 H34             2.3510773939            -8.8213178583            8.9330163583

 H35             2.0108712928            -7.3197620208            8.0735831622

 C36             3.9919966832            -8.1384734853            7.7236265633

 O37             5.1120944335            -8.4035069050            8.2085529247

 C38             3.2000308516            -7.0907857579            9.9116221294

 H39             3.8740790035            -7.7281882443           10.4954376186

 H40             2.3117284493            -6.9083172007           10.5232136651

 C41             3.8713889706            -5.7804686168            9.6085519302

 N42             4.9716495448            -5.7647739487            8.7692500928

 H43             5.4134327256            -6.6255117541            8.4327669898

 C44             3.6039001152            -4.4533584108            9.8631204015

 H45             2.8293343097            -4.0060761340           10.4663527552

 C46             5.2901659362            -4.4942381941            8.4870878990

 H47             5.9894342378            -4.2021686942            7.7266812668

 N48             4.5042038888            -3.6687608578            9.1609328963

 N49             3.7976321406            -7.9633031409            6.4029564368

 H50             2.8643690807            -7.6802108844            6.0854853358

 C51             4.9172534511#           -7.8463447999#           5.4688656791#

 H52             4.6635018060            -8.3858597015            4.5481498438

 H53             5.7804547959            -8.3333902882            5.9241485947

 C54             5.2442715669            -6.3564412135            5.1492867396

 H55             6.1229327335            -6.3287868797            4.4911976913

 H56             5.5459067160            -5.8703851750            6.0809151681

 C57             4.0865468807            -5.5548182632            4.5127263181

 H58             3.1217651795            -5.8149013494            4.9682332875

 H59             3.9722782787            -5.7924085109            3.4477557418

 C60             4.1583506637            -4.0325593904            4.6028285171

 O61             3.2094220284            -3.3919087871            4.0488273551

 O62             5.1135631332            -3.4830817145            5.2397701919

 C63             9.1595118028#            0.1191581060#          11.4478229429#

 H64             9.8203459372            -0.1593498796           10.6199117993

 H65             9.7326016642             0.0001453162           12.3766547469

 C66             7.9313159086            -0.7864490576           11.4978936579

 H67             7.2244858145            -0.4229486219           12.2505130393

 H68             8.2160711265            -1.7989762171           11.8110272946

 C69             7.1568880416            -0.9080764805           10.1900435749

 O70             7.6072286145            -0.3249191142            9.1607227765

 O71             6.1220814703            -1.6440786378           10.2165974212

 C72             8.7681704574             1.5943347144           11.3252091028

 O73             7.6332178083             2.0042401726           11.5920904829

 N74             9.7776883034             2.4287864799           10.9741239924

 H75            10.6372448459             2.0303410905           10.6207548788

 C76             9.5677777604#            3.8588147862#          10.7651888697#

 H77            10.5127162409             4.3813652817           10.9209014308

 H78             8.8343229356             4.2014697024           11.4983393800

 C79             9.1074587221             4.1347206467            9.3152598749

 O80             9.9103003595             4.4248808261            8.4349280591

 N81             7.7657649948             4.0002555696            9.1128513781

 H82             7.1857321191             3.6304862993            9.8572438127

 C83             7.1721121643             4.0865248704            7.7949370476

 H84             6.4252988059             4.8849936585            7.7486936713

 H85             7.9843323107             4.3217067520            7.0988857274

 C86             6.4796121288             2.7887436806            7.3574239363

 O87             5.3590146825             2.8423165888            6.8286522147

 O88             7.1643254053             1.7088734452            7.5818365587

 C89            11.5816619294#           -0.6170832909#           3.4808991531#

 H90            12.4441751961#           -1.2180927646#           3.7284028885#

 H91            11.8304270544#            0.3821369961#           3.1552080301#

 H92            11.1176478048            -1.0971780227            2.6078622115

 C93            10.5270638946            -0.4645638312            4.5928640396

 H94             9.9083669152             0.4097378790            4.3546573012

 H95            10.9902591380            -0.2430028896            5.5606448285

 C96             9.5995866544            -1.6822844959            4.7067538941

 H97            10.1175081943            -2.5021151384            5.2234414861

 H98             9.3456189911            -2.0641815976            3.7137284638

 C99             8.2795474145            -1.3944032864            5.4132682791

 O100            7.2470869667            -1.8695144328            4.8395587073

 O101            8.2808079699            -0.7315579532            6.4972805380

 C102           10.8187242978#            4.2285683259#          -0.8150213469#

 H103           11.5403643581#            3.4839223640#          -0.5131309583#

 H104           11.2010929692#            5.2306259359#          -0.6882234012#

 H105           10.6689348773             4.0988978204           -1.8939487552

 C106            9.4811353847             4.0119277468           -0.0879774859

 H107            9.0294356923             3.0891824596           -0.4827222644

 H108            8.7840532048             4.8229160699           -0.3397323069

 C109            9.6202287895             3.9102766474            1.4387464583

 H110           10.0797676154             4.8291266476            1.8250500369

 H111           10.3010535735             3.0879960020            1.6968317757

 C112            8.2969353681             3.7147379263            2.1931449556

 H113            7.6231432216             4.5603958847            1.9907261596

 H114            8.4905106092             3.6917723187            3.2704072818

 N115            7.6458312525             2.4513592620            1.8335206220

 H116            7.6820927712             2.1684368512            0.8633471401

 C117            6.8178251543             1.7328943330            2.6205836510

 N118            6.4274395773             2.2010042748            3.8148853201

 H119            6.0219785945             1.5606470248            4.5026636847

 H120            6.3824066487             3.1883354213            4.0170547047

 N121            6.3874010969             0.5404783351            2.2110987501

 H122            6.9351460860            -0.1117624432            1.6424046788

 H123            5.6933513222             0.0333737290            2.7775983289

 C124           11.8423977358#           -5.3930895903#           7.2492548905#

 H125           11.2522022451#           -5.9724612236#           6.5547079783#

 H126           12.7964629221#           -5.8524491317#           7.4617420638#

 H127           12.0211980382            -4.3931434198            6.8418811346

 C128           11.1765605765            -5.3467260121            8.6383398772

 H129           11.8789718011            -4.8973750617            9.3549155085

 H130           11.0137236471            -6.3763602879            8.9849481329

 C131            9.8876715849            -4.6015222217            8.7137842949

 N132            9.0485143584            -4.6754157980            9.8207100428

 C133            9.2865771513            -3.7121819311            7.8687970956

 H134            9.5986367827            -3.3527721662            6.9063212734

 C135            7.9908910836            -3.8695548971            9.6502891054

 H136            7.1702997347            -3.7162419679           10.3334715476

 N137            8.1278558002            -3.2719130723            8.4712888317

 H138            7.4102998542            -2.6370353041            8.0509181752

 C139            6.0989363243#           -4.2351881156#          -1.2221974272#

 H140            5.3833631522#           -4.5684339759#          -1.9592871588#

 H141            7.1094170057#           -4.1301811051#          -1.5886720131#

 H142            5.8003351259            -3.2114083586           -0.9649072576

 C143            5.9753881694            -5.0819282354            0.0612796763

 H144            5.9661597359            -6.1542516814           -0.1564096630

 H145            6.8257611551            -4.8670752642            0.7195789889

 C146            4.7009299448            -4.6757465769            0.8076164133

 O147            4.6512614928            -3.4743819927            1.1988691945

 O148            3.7613341214            -5.5225955268            0.9704524200

 C149           -1.8288756382#          -11.9867176039#           4.2485683710#

 H150           -2.3586933338#          -12.7180953892#           3.6563005447#

 H151           -2.3284063879#          -11.7832501405#           5.1842338233#

 H152           -0.8504230595           -12.4140627043            4.5050165908

 C153           -1.6532387464           -10.6558468133            3.4820005347

 H154           -2.5687571347           -10.4477368135            2.9095862496

 H155           -1.5585719349            -9.8364733363            4.2064899794

 C156           -0.4391031874           -10.6313184976            2.5375933436

 H157            0.4826892544           -10.5674465034            3.1341480328

 H158           -0.3781193872           -11.5865161705            1.9983746332

 C159           -0.4764273312            -9.5174544064            1.4753596972

 H160            0.3944894905            -9.6022030166            0.8112201496

 H161           -1.3570742502            -9.6831539608            0.8402555100

 C162           -0.5766339035            -8.0673046824            1.9626957932

 H163           -0.8749491355            -7.4304545913            1.1253905608

 H164           -1.3233600014            -7.9441661600            2.7506550669

 N165            0.7124148788            -7.4883691226            2.4868070365

 H166            0.7973202094            -6.4991826927            2.1600079811

 H167            0.7605708384            -7.4519635638            3.5302486853

 H168            1.5692865127            -7.9507964458            2.1033869845

 Cl169           0.8519466411            -6.6993198737            5.5536851457

 O170            1.2585265045            -1.6274809398            4.4026460131

 H171            0.3098510308            -2.1550066839            2.8578644332

 O172            4.8957097556            -1.2640902070            3.6499209841

 O173            7.1182118067            -2.1354233477            1.7460558623

 H174            7.0442001489            -2.1507114319            2.7167773363

 H175            6.2597240359            -2.5286412207            1.4629637523

 O176            1.1695339695             0.3080486211            6.5030746640

 H177            1.0518895010            -0.3168295604            5.7447740481

 H178            0.9282727694            -0.1929476563            7.2996324895

 O179            2.8880223950             2.9211545003            8.5128297145

 H180            3.6709786802             3.1116774648            9.0697925276

 H181            2.3188790475             2.3878103597            9.0993192986

 O182            5.1302833224             2.3312865641           10.1726966750

 H183            5.1495696763             1.4920152079            9.6661865234

 H184            5.7999296442             2.2142912919           10.8721249555

 O185            2.8144886767            -1.8005997990            1.9501396771

 H186            3.4182109050            -2.5658439235            1.6774499328

 H187            0.9240526137            -4.4023130687            6.2037194291

 O188            3.0919705287            -1.9080038303            7.8952522851

 O189            1.1754146147            -4.9901331915            1.2817072955

 H190            2.1809926395            -5.0390592581            1.2524209915

 H191            0.9108237122            -4.9864325755            0.3472673565

 O192            0.2860522808            -2.4131942032            1.9079184016

 H193            0.4389334930            -3.3834902778            1.8925219932

 H194            1.8391453050            -2.0139356824            1.7556746312

 O195            3.1207322703            -8.1524087694            1.2135535733

 H196            3.4844648762            -7.2510959137            1.0124650036

 H197            3.8604236582            -8.6602761264            1.5767719416

 O198            0.9728068235            -3.4475768601            6.4231427156

 H199            1.1947760616            -2.3872609097            5.0500330067

 H200            1.6713943112            -3.3427978504            7.0928279309

 H201            3.2160465525            -1.7201465682            6.9143311470

 H202            9.1997716654            -5.2579866704           10.6350265043

Previous 202 atom structure with an oxyl radical 202(a)

 Mn1             3.1710726127            -1.4389674450            3.6572416401

 Mn2             4.6003764623            -1.4801533589            8.6436015537

 Mn3             5.3883753060            -1.5238690377            5.3473478411

 Mn4             6.6552123476            -0.0402521318            7.3893971599

 O5              3.6550632579            -1.2491862130            5.6203892469

 O6              5.8219117939             0.2020801148            5.8013319954

 O7              5.1733160276             0.2730462275            8.3710330185

 O8              5.9604390429            -1.8186951702            7.2961496219

 Ca9             3.5436395312             1.1067629571            6.7028520274

 C10             0.6675458548#            1.9515092497#           1.7063473753#

 H11             1.2251835819#            1.9058026286#           0.7825777167#

 H12            -0.3243897689#            2.3686415809#           1.6143241443#

 H13             0.4917092930             0.8987590858            1.9612850487

 C14             1.4664557918             2.5859830687            2.8603760597

 H15             0.7865013195             3.0161232850            3.6079024553

 H16             2.0839677705             3.4280968440            2.5258447078

 C17             2.3886423342             1.6564933494            3.6698180771

 O18             3.0796102233             2.1527793960            4.5878304426

 O19             2.3523566992             0.3998312015            3.3931471333

 C20             1.0582597107#            1.1829964383#          13.4563134067#

 H21             0.3702239585#            0.3601390311#          13.5824629773#

 H22             0.7336468126#            2.1291270407#          13.8635787554#

 H23             1.9380400782             0.9079460442           14.0581563889

 C24             1.4825604068             1.2410149566           11.9927596425

 H25             2.3133665005             1.9452808150           11.8571687656

 H26             0.6649529192             1.5840283419           11.3487430506

 C27             1.9215550147            -0.1662466474           11.5735260619

 H28             1.0470716516            -0.8329755554           11.5285957722

 H29             2.6009013999            -0.5985473476           12.3153444228

 C30             2.5993052029            -0.2163829982           10.2261121168

 O31             2.2628905255             0.5499994962            9.3080350749

 O32             3.5410725318            -1.1141899654           10.1610174482

 C33             2.7844498498#           -7.8608083460#           8.6257440687#

 H34             2.4423344034            -8.8273954210            9.0164830979

 H35             1.9639587271            -7.4131637906            8.0587723227

 C36             3.9958003761            -8.1097962848            7.7322223961

 O37             5.1109923935            -8.3861166658            8.2114697824

 C38             3.1514082222            -6.9594744442            9.8422439174

 H39             3.8634047218            -7.5042462749           10.4739150060

 H40             2.2532735560            -6.7844924422           10.4412705279

 C41             3.7425186324            -5.6387216670            9.4338523549

 N42             4.8164519545            -5.6290435975            8.5599402378

 H43             5.3057807884            -6.4746369189            8.2683911125

 C44             3.4472807550            -4.3100456949            9.6386505477

 H45             2.6886577285            -3.8566834962           10.2570007794

 C46             5.0913299313            -4.3647721258            8.2144513251

 H47             5.7788469056            -4.0854106191            7.4391811068

 N48             4.3048373030            -3.5298642194            8.8755936841

 N49             3.8007816542            -7.9037505956            6.4113810715

 H50             2.9044377319            -7.5032610229            6.1354034629

 C51             4.9172534139#           -7.8463446879#           5.4688656670#

 H52             4.6228271903            -8.3757736547            4.5540090311

 H53             5.7498456192            -8.3835868864            5.9249322484

 C54             5.3321620485            -6.3949617368            5.1101199747

 H55             6.2092142315            -6.4426976462            4.4513094768

 H56             5.6632788775            -5.8874829971            6.0191401591

 C57             4.2085189459            -5.5836392621            4.4411344787

 H58             3.2449972675            -5.7819367409            4.9286446901

 H59             4.0786363719            -5.8692746393            3.3921734448

 C60             4.2908037365            -4.0651386503            4.4749554362

 O61             3.3739152313            -3.4467381611            3.8601935515

 O62             5.2211196141            -3.5002272259            5.1452267863

 C63             9.1595117507#            0.1191581166#          11.4478229268#

 H64             9.8451382983            -0.1462207795           10.6360569434

 H65             9.7078975314            -0.0069599524           12.3908223284

 C66             7.9388158080            -0.8018950371           11.4549803066

 H67             7.2079612183            -0.4512579932           12.1904351173

 H68             8.2286368727            -1.8129184721           11.7698310445

 C69             7.1967315042            -0.9285853856           10.1262262081

 O70             7.6911300073            -0.3438488503            9.1115004292

 O71             6.1544122362            -1.6487321956           10.1189429869

 C72             8.7671573930             1.5937933257           11.3367075482

 O73             7.6365142467             1.9993510266           11.6230748375

 N74             9.7757282742             2.4295661915           10.9794158373

 H75            10.6256547744             2.0289510523           10.6052755718

 C76             9.5677777413#            3.8588147826#          10.7651888354#

 H77            10.5001405140             4.3885148035           10.9679071220

 H78             8.7966543664             4.1921752411           11.4625887375

 C79             9.1803448512             4.1266420877            9.2933513057

 O80            10.0357586223             4.3584484082            8.4456079646

 N81             7.8438568045             4.0535396824            9.0305654657

 H82             7.2075128239             3.7096490370            9.7418212548

 C83             7.3216173883             4.1072100955            7.6798603667

 H84             6.5962189880             4.9190666771            7.5702410655

 H85             8.1733874685             4.2960151211            7.0183556013

 C86             6.6197849453             2.8064766223            7.2618176125

 O87             5.4934330728             2.8458291882            6.7482473983

 O88             7.3160903747             1.7303419256            7.4896224717

 C89            11.5816618811#           -0.6170833399#           3.4808991581#

 H90            12.4441752041#           -1.2180927495#           3.7284028972#

 H91            11.8304270585#            0.3821369980#           3.1552080391#

 H92            11.1246688314            -1.0974200057            2.6037358015

 C93            10.5187486294            -0.4577911295            4.5691758741

 H94             9.8420658927             0.3486331280            4.2589383140

 H95            10.9531310944            -0.1302423837            5.5198266243

 C96             9.6863622736            -1.7297419400            4.7785717915

 H97            10.2530257422            -2.4232216735            5.4136114215

 H98             9.4944670955            -2.2349906933            3.8279831163

 C99             8.3418723384            -1.4162155981            5.4108912411

 O100            7.3163632050            -1.8584007793            4.8084243608

 O101            8.3397127533            -0.7298106836            6.4824464953

 C102           10.8187242607#            4.2285682665#          -0.8150213234#

 H103           11.5403643809#            3.4839223827#          -0.5131309671#

 H104           11.2010929518#            5.2306259398#          -0.6882233790#

 H105           10.6783752106             4.0978218011           -1.8955393844

 C106            9.4723445620             4.0281852274           -0.1064747338

 H107            9.0428802334             3.0783026611           -0.4588119780

 H108            8.7729758845             4.8162529315           -0.4193313520

 C109            9.5744833912             4.0142519859            1.4249584057

 H110           10.0503144426             4.9418077037            1.7689497656

 H111           10.2233627268             3.1895722764            1.7484126043

 C112            8.2232610260             3.9018562276            2.1450517537

 H113            7.5979768676             4.7745796188            1.9096422916

 H114            8.3823632034             3.8874575674            3.2268037890

 N115            7.5128827424             2.6703629762            1.7870299739

 H116            7.4917808915             2.4108482812            0.8101979776

 C117            6.8122396644             1.8797698235            2.6225052616

 N118            6.5390900357             2.2814608350            3.8751258441

 H119            6.1644838806             1.6055961677            4.5485530694

 H120            6.3752389668             3.2552238476            4.0871467182

 N121            6.3972947841             0.6819129574            2.2172414300

 H122            6.8682189256             0.0944915649            1.5283037698

 H123            5.7544349044             0.1386253534            2.8076830063

 C124           11.8423977117#           -5.3930895483#           7.2492548582#

 H125           11.2522022606#           -5.9724612372#           6.5547079766#

 H126           12.7964629103#           -5.8524491458#           7.4617420869#

 H127           12.0199379612            -4.3943341999            6.8381226882

 C128           11.1512930034            -5.3384059754            8.6259911118

 H129           11.8211251702            -4.8434613453            9.3436707893

 H130           11.0216808649            -6.3648707751            8.9955486479

 C131            9.8302721098            -4.6422915716            8.6614382377

 N132            8.9926710808            -4.7014038852            9.7720475797

 C133            9.1930676466            -3.8131980779            7.7816548419

 H134            9.4694028713            -3.4884253670            6.7961729937

 C135            7.9072924131            -3.9383765694            9.5732577591

 H136            7.0922507181            -3.7702883737           10.2599623153

 N137            8.0213154670            -3.3860787831            8.3707227889

 H138            7.3088704511            -2.7407378267            7.9450119155

 C139            6.0989362190#           -4.2351880766#          -1.2221973575#

 H140            5.3833631907#           -4.5684339916#          -1.9592871890#

 H141            7.1094170095#           -4.1301811330#          -1.5886720105#

 H142            5.7952093080            -3.2219101890           -0.9434065216

 C143            5.9997433650            -5.1225558304            0.0330186434

 H144            6.0311108615            -6.1887026531           -0.2108248708

 H145            6.8331406362            -4.8946300939            0.7091141926

 C146            4.6999333443            -4.7637644548            0.7486832218

 O147            4.5701386487            -3.5452087442            1.0372816220

 O148            3.8153052286            -5.6614927895            0.9595503339

 C149           -1.8288755010#          -11.9867176062#           4.2485683801#

 H150           -2.3586933809#          -12.7180953454#           3.6563005329#

 H151           -2.3284064258#          -11.7832501224#           5.1842337991#

 H152           -0.8391609343           -12.3934354560            4.4894771836

 C153           -1.7164372545           -10.6259977310            3.5081237483

 H154           -2.5552010732           -10.5265235580            2.8053009767

 H155           -1.8557858659            -9.8234013138            4.2441402682

 C156           -0.4028242703           -10.3705140112            2.7513094422

 H157            0.4357528187           -10.3490011239            3.4634784852

 H158           -0.1991944601           -11.2079645085            2.0708754872

 C159           -0.4311696297            -9.0763775103            1.9114528736

 H160            0.4598384896            -9.0191495032            1.2727730582

 H161           -1.2896946152            -9.1323408811            1.2302167781

 C162           -0.5611532028            -7.7593229919            2.6979218259

 H163           -1.0871727706            -7.0079968585            2.1043068516

 H164           -1.1238220235            -7.8916199904            3.6237381547

 N165            0.7653772324            -7.1370713344            3.0689079580

 H166            0.9253817320            -6.2972767581            2.4641809243

 H167            0.8014969413            -6.8137995241            4.0615506719

 H168            1.5873555202            -7.7407129693            2.8635027115

 Cl169           1.0179908154            -5.8603366098            6.0098010474

 O170            1.1724566123            -1.7906507567            4.0861089465

 H171            0.2966212845            -2.3776725177            2.4115759853

 O172            4.9594924621            -1.2121695267            3.6079511827

 O173            6.9666366164            -2.0032917476            1.5469438442

 H174            6.8607934742            -2.0354974127            2.5132717543

 H175            6.1594889163            -2.4699206497            1.2346048961

 O176            1.0724730806             0.3865497891            6.8207890486

 H177            1.0070002231            -0.5864057659            6.7725563180

 H178            0.9923160057             0.5487557615            7.7811891273

 O179            3.0304244507             2.9338207364            8.3258319296

 H180            3.8470240346             3.2277313631            8.7745272431

 H181            2.5562655510             2.4439706112            9.0241151468

 O182            5.2781930871             2.5065342212           10.0732064397

 H183            5.3166522391             1.6562393323            9.5843050670

 H184            5.8867963396             2.3810542747           10.8254200979

 O185            2.8945477466            -1.6515837728            1.6030291444

 H186            3.4271197435            -2.4540829119            1.3347056315

 H187            1.0010168698            -3.6876723371            6.4437106572

 O188            3.2679111068            -1.3496351611            7.6285556311

 O189            1.2650751287            -5.1418776909            1.1731348876

 H190            2.2802280140            -5.1855837548            1.1570357870

 H191            1.0025086212            -5.4419066937            0.2876404424

 O192            0.3648842091            -2.5135304202            1.4483024037

 H193            0.6205557794            -3.4599391799            1.3592182377

 H194            1.9357045128            -1.8550441145            1.3991107820

 O195            3.1093697932            -8.1170986901            1.9044249230

 H196            3.5114151121            -7.3180298723            1.4798792667

 H197            3.7976488934            -8.7968165362            1.9147155465

 O198            1.1340195202            -2.7073108727            6.4858045585

 H199            1.1261686544            -2.2302668543            5.0170857743

 H200            2.0330234290            -2.5513572493            6.8541492438

 H201            0.8323219266            -0.8796223103            4.1726648227

 H202            9.1651008477            -5.2453137731           10.6083465172

Modified 202 atom structure with an oxyl radical 202(b)

 Mn1             3.0887210000            -1.3912010000            3.8571450000

 Mn2             4.6223700000            -1.5400320000            8.7122460000

 Mn3             5.3646140000            -1.5794480000            5.4156820000

 Mn4             6.6449310000            -0.0649040000            7.4290330000

 O5              3.6674420000            -1.2229610000            5.7601090000

 O6              5.8247300000             0.1716490000            5.8493640000

 O7              5.1639430000             0.2264210000            8.4266240000

 O8              5.9800750000            -1.8431890000            7.3681130000

 Ca9             3.5443380000             1.0367290000            6.8205490000

 C10             0.6675320000#            1.9515150000#           1.7063580000#

 H11             1.2251930000#            1.9058060000#           0.7825910000#

 H12            -0.3243980000#            2.3686340000#           1.6143130000#

 H13             0.4910390000             0.9016090000            1.9766650000

 C14             1.4654330000             2.6027260000            2.8465900000

 H15             0.7881850000             3.0761660000            3.5663770000

 H16             2.1096570000             3.4144280000            2.4816410000

 C17             2.3591550000             1.6578590000            3.6629930000

 O18             2.7295530000             2.0605630000            4.8039250000

 O19             2.6815440000             0.5317180000            3.1637010000

 C20             1.0582470000#            1.1829920000#          13.4563230000#

 H21             0.3702150000#            0.3601410000#          13.5824720000#

 H22             0.7336500000#            2.1291290000#          13.8635800000#

 H23             1.9359440000             0.9065100000           14.0606260000

 C24             1.4856310000             1.2266080000           11.9918220000

 H25             2.3378640000             1.9045280000           11.8542480000

 H26             0.6767860000             1.5891750000           11.3471480000

 C27             1.8804450000            -0.1987220000           11.5822510000

 H28             0.9793640000            -0.8272170000           11.5205030000

 H29             2.5265210000            -0.6526370000           12.3400460000

 C30             2.5815020000            -0.2855250000           10.2489760000

 O31             2.2309910000             0.4331850000            9.2929600000

 O32             3.5453920000            -1.1538870000           10.2267590000

 C33             2.7844220000#           -7.8608470000#           8.6257600000#

 H34             2.4177520000            -8.8268810000            8.9949990000

 H35             1.9723590000            -7.3976750000            8.0569580000

 C36             3.9962380000            -8.1091600000            7.7287390000

 O37             5.1225550000            -8.3306840000            8.2193910000

 C38             3.1665520000            -6.9937180000            9.8620190000

 H39             3.8622090000            -7.5693570000           10.4837270000

 H40             2.2692990000            -6.8101440000           10.4600340000

 C41             3.7929370000            -5.6780670000            9.4905470000

 N42             4.8945210000            -5.6667990000            8.6530420000

 H43             5.3611950000            -6.5257000000            8.3495950000

 C44             3.4985990000            -4.3482050000            9.6994960000

 H45             2.7206280000            -3.8977830000           10.2959340000

 C46             5.1890040000            -4.4017510000            8.3311820000

 H47             5.8948200000            -4.1205760000            7.5733870000

 N48             4.3861830000            -3.5684260000            8.9750520000

 N49             3.8014970000            -7.9490890000            6.4079150000

 H50             2.8573140000            -7.7135970000            6.0828640000

 C51             4.9172680000#           -7.8463570000#           5.4688570000#

 H52             4.6385530000            -8.3739810000            4.5482470000

 H53             5.7686110000            -8.3650900000            5.9128270000

 C54             5.2804190000            -6.3806480000            5.1464260000

 H55             6.1412240000            -6.3757180000            4.4648290000

 H56             5.6148010000            -5.8922160000            6.0647150000

 C57             4.1124460000            -5.5949070000            4.5354290000

 H58             3.1624090000            -5.8659690000            5.0137720000

 H59             3.9790020000            -5.8353780000            3.4738080000

 C60             4.1657370000            -4.0797450000            4.6197070000

 O61             3.2123980000            -3.4558970000            4.0866800000

 O62             5.1436140000            -3.5241290000            5.2391210000

 C63             9.1595090000#            0.1191630000#          11.4478550000#

 H64             9.8318700000            -0.1567390000           10.6284500000

 H65             9.7277530000             0.0159580000           12.3820670000

 C66             7.9488660000            -0.8051230000           11.4923750000

 H67             7.2265190000            -0.4532550000           12.2356430000

 H68             8.2498470000            -1.8119940000           11.8093270000

 C69             7.1985640000            -0.9392400000           10.1735720000

 O70             7.6836250000            -0.3591480000            9.1525110000

 O71             6.1643840000            -1.6674520000           10.1793870000

 C72             8.7600720000             1.5895040000           11.3133630000

 O73             7.6214330000             1.9939880000           11.5682470000

 N74             9.7697250000             2.4273750000           10.9672150000

 H75            10.6337410000             2.0288210000           10.6257230000

 C76             9.5677990000#            3.8588180000#          10.7652180000#

 H77            10.5169280000             4.3742190000           10.9194470000

 H78             8.8404130000             4.2032390000           11.5042240000

 C79             9.1006450000             4.1520010000            9.3245360000

 O80             9.8981030000             4.4553100000            8.4450790000

 N81             7.7569000000             4.0242560000            9.1288960000

 H82             7.1846310000             3.6318730000            9.8678830000

 C83             7.1604130000             4.1077090000            7.8137130000

 H84             7.9646470000             4.3806410000            7.1220880000

 H85             6.3854470000             4.8795330000            7.7794250000

 C86             6.5175050000             2.7898810000            7.3584690000

 O87             5.3854650000             2.8035900000            6.8492560000

 O88             7.2550690000             1.7386250000            7.5426100000

 C89            11.5816560000#           -0.6170440000#           3.4809120000#

 H90            12.4441590000#           -1.2181060000#           3.7283860000#

 H91            11.8304410000#            0.3821460000#           3.1552020000#

 H92            11.1218520000            -1.0965530000            2.6050360000

 C93            10.5284400000            -0.4729850000            4.5859010000

 H94             9.8838300000             0.3776120000            4.3304420000

 H95            10.9825740000            -0.2205730000            5.5502540000

 C96             9.6420100000            -1.7167820000            4.7184610000

 H97            10.1817180000            -2.5058720000            5.2604340000

 H98             9.4035080000            -2.1273050000            3.7330130000

 C99             8.3202100000            -1.4291000000            5.4124430000

 O100            7.2869620000            -1.9121370000            4.8562450000

 O101            8.3333700000            -0.7410690000            6.4819130000

 C102           10.8187760000#            4.2286350000#          -0.8151260000#

 H103           11.5403320000#            3.4839120000#          -0.5131410000#

 H104           11.2011380000#            5.2306370000#          -0.6882010000#

 H105           10.6533460000             4.0971980000           -1.8910740000

 C106            9.5013450000             4.0074070000           -0.0543770000

 H107            9.0366270000             3.0884940000           -0.4427200000

 H108            8.7960140000             4.8211580000           -0.2706390000

 C109            9.7070260000             3.8896530000            1.4635010000

 H110           10.1694560000             4.8109690000            1.8397530000

 H111           10.4118990000             3.0756950000            1.6798430000

 C112            8.4262550000             3.6616480000            2.2761440000

 H113            7.7265740000             4.4953600000            2.1140840000

 H114            8.6753650000             3.6342200000            3.3422300000

 N115            7.7848360000             2.3883900000            1.9356270000

 H116            7.8164910000             2.0949180000            0.9685580000

 C117            6.9332890000             1.7026160000            2.7259890000

 N118            6.5489390000             2.1954890000            3.9110640000

 H119            6.1715540000             1.5645140000            4.6274880000

 H120            6.6307890000             3.1743120000            4.1373500000

 N121            6.4555400000             0.5239450000            2.3260090000

 H122            6.9834460000            -0.1707630000            1.7816130000

 H123            5.7465610000             0.0586200000            2.9071020000

 C124           11.8423960000#           -5.3931000000#           7.2492800000#

 H125           11.2521970000#           -5.9724480000#           6.5547070000#

 H126           12.7964650000#           -5.8524550000#           7.4617370000#

 H127           12.0175380000            -4.3913660000            6.8447930000

 C128           11.1762820000            -5.3546710000            8.6402180000

 H129           11.8707940000            -4.8895390000            9.3543360000

 H130           11.0344190000            -6.3866640000            8.9887300000

 C131            9.8724130000            -4.6358220000            8.7155440000

 N132            9.0214130000            -4.7431150000            9.8114450000

 C133            9.2633240000            -3.7448430000            7.8780960000

 H134            9.5736850000            -3.3665260000            6.9218620000

 C135            7.9492990000            -3.9545110000            9.6421230000

 H136            7.1233270000            -3.8197580000           10.3228200000

 N137            8.0888830000            -3.3375860000            8.4731130000

 H138            7.3749760000            -2.6946630000            8.0540400000

 C139            6.0989480000#           -4.2352440000#          -1.2222040000#

 H140            5.3833500000#           -4.5684120000#          -1.9592940000#

 H141            7.1094170000#           -4.1301820000#          -1.5886810000#

 H142            5.8021870000            -3.2083910000           -0.9759020000

 C143            5.9826970000            -5.0604130000            0.0724950000

 H144            5.9300570000            -6.1351520000           -0.1266040000

 H145            6.8597520000            -4.8689480000            0.7036960000

 C146            4.7472040000            -4.6004080000            0.8539940000

 O147            4.7326790000            -3.3643920000            1.1616140000

 O148            3.8243380000            -5.4157160000            1.1343630000

 C149           -1.8288990000#          -11.9867260000#           4.2485600000#

 H150           -2.3586950000#          -12.7181170000#           3.6563130000#

 H151           -2.3284030000#          -11.7832450000#           5.1842350000#

 H152           -0.8561210000           -12.4243250000            4.5113360000

 C153           -1.6241360000           -10.6727290000            3.4593220000

 H154           -2.5403550000           -10.4440290000            2.8961170000

 H155           -1.4854070000            -9.8426440000            4.1642610000

 C156           -0.4233410000           -10.7263570000            2.4962250000

 H157            0.5067830000           -10.6679520000            3.0803420000

 H158           -0.4005210000           -11.7088650000            2.0049480000

 C159           -0.4210730000            -9.6702010000            1.3748280000

 H160            0.4473340000            -9.8319490000            0.7214920000

 H161           -1.3062400000            -9.8332010000            0.7449400000

 C162           -0.4537270000            -8.1908200000            1.7723740000

 H163           -0.6059520000            -7.5798400000            0.8782440000

 H164           -1.2699350000            -7.9722590000            2.4664890000

 N165            0.8047560000            -7.6858310000            2.4099360000

 H166            0.8929400000            -6.6302100000            2.2682540000

 H167            0.8210430000            -7.8153780000            3.4393000000

 H168            1.6761910000            -8.0684820000            1.9796660000

 Cl169           0.7863770000            -6.9456300000            5.5156170000

 O170            1.1506440000            -1.4846400000            4.4453520000

 H171            1.2507910000             3.3282240000            7.2792220000

 O172            4.8895280000            -1.2864970000            3.6988670000

 O173            7.1862780000            -2.0990230000            1.7773980000

 H174            7.1074380000            -2.1834060000            2.7447300000

 H175            6.3399630000            -2.4973210000            1.4581490000

 O176            1.1086780000             0.2807020000            6.6629500000

 H177            1.0368100000            -0.0566590000            7.5748930000

 H178            0.6941000000             1.1876510000            6.6565340000

 O179            2.8652930000             2.8700880000            8.4575420000

 H180            3.6645150000             3.1930100000            8.9217120000

 H181            2.4553760000             2.2593630000            9.1070130000

 O182            5.1312330000             2.4215110000           10.1510990000

 H183            5.2215160000             1.5830160000            9.6466330000

 H184            5.7577650000             2.3197340000           10.8909170000

 O185            2.7040040000            -1.9502670000            1.8680400000

 H186            3.4551570000            -2.5793720000            1.5638840000

 H187            0.5351650000            -4.8486800000            4.9430140000

 O188            3.2705150000            -1.4743740000            7.7259060000

 O189            1.1655180000            -5.0479140000            1.9253630000

 H190            2.1203170000            -4.9723710000            1.6867840000

 H191            1.0400580000            -4.5222980000            2.7412300000

 O192            0.7433800000             2.9495920000            6.5354050000

 H193            1.3821400000             2.9611520000            5.7894150000

 H194            2.7082350000            -1.1711670000            1.2892990000

 O195            3.2604370000            -8.0806510000            1.1207490000

 H196            3.5526370000            -7.1394570000            1.0033200000

 H197            4.0440140000            -8.5587530000            1.4267610000

 O198            0.2465060000            -4.0232850000            4.4724940000

 H199            0.8481170000            -2.4292120000            4.5775450000

 H200           -0.7153510000            -4.1174420000            4.3866220000

 H201            1.0300680000            -0.9769830000            5.2868860000

 H202            9.1750360000            -5.3378650000           10.6161480000

247 atom structure with an OH

 Mn1             2.8905806753            -1.4782751985            3.7673862183

 Mn2             4.6191175024            -1.5058048176            8.7117297034

 Mn3             5.2686355685            -1.4490584183            5.1812479536

 Mn4             6.4878550124             0.0173753203            7.2762685063

 O5              3.4897350756            -1.2064330263            5.4465585684

 O6              5.6256175442             0.2702595086            5.7140070826

 O7              5.0461538911             0.2666646632            8.3589314280

 O8              5.7936524367            -1.7541727037            7.1897671197

 Ca9             3.2928249833             1.1038127599            6.8799242881

 C10             2.1876270068#            1.2855489692#          -1.5249169827#

 C11             1.4949021776             0.9053827542           -0.2212722425

 O12             1.2413849423            -0.2719158552            0.0574471399

 C13             3.5143129777             0.5226074178           -1.6506661680

 O14             4.3875039056             0.8881434808           -0.5952214380

 H15             1.5242052674             1.0117475154           -2.3546316115

 H16             3.2987101656            -0.5530419418           -1.6342133917

 H17             3.9665796072             0.7600818384           -2.6257462176

 H18             4.7344085148             0.0635517783           -0.1885519050

 H19             2.3708397902             2.3648169208           -1.5818168366

 N20             1.1555094314             1.9354221185            0.6041277426

 C21             0.4831778864             1.7530285761            1.8831371973

 C22            -0.9597906992             2.2561220614            1.7813842890

 O23            -1.6537796890             2.5753095137            2.7230992995

 C24             1.2408884223             2.3549506891            3.0742858405

 C25             2.3244205955             1.4737298817            3.7033127738

 O26             2.9790443457             1.9408145775            4.6518631963

 O27             2.4449737667             0.3015863866            3.1903289358

 H28             1.4366841081             2.8699463689            0.3385309177

 H29             0.4065584569             0.6662839761            2.0218446159

 H30             0.5288790344             2.5547683696            3.8824461795

 H31             1.6869772553             3.3232377851            2.8172736117

 H32            -1.3635463039             2.2837229841            0.7480106010

 C33             1.0582598247#            1.1829964018#          13.4563130800#

 H34             0.1026715283             0.6433952921           13.4472409132

 H35             0.8575953490             2.1926637354           13.8336964954

 H36             1.7142061849             0.6843213416           14.1801448657

 C37             1.6871614998             1.2133706834           12.0809484171

 H38             2.6513055502             1.7339584149           12.1259002222

 H39             1.0467067975             1.7514899012           11.3753394734

 C40             1.9215543200            -0.2171714602           11.5945399655

 H41             0.9592887630            -0.7281310050           11.4433122514

 H42             2.4910286181            -0.7899161058           12.3305681117

 C43             2.6541766457            -0.2333055688           10.2822801261

 O44             2.3169678952             0.5258352914            9.3530530978

 O45             3.6399506339            -1.0820674768           10.2449076951

 C46             3.6972756430            -8.1562798932            8.3179079053

 H47             3.0098436281            -8.9390134542            7.9843772970

 H48             4.2384914071            -8.5227098149            9.1966823503

 C49             4.7396681859            -7.8707766031            7.2464379785

 O50             5.8559363084            -7.4103462731            7.5671683788

 C51             2.8380944076            -6.9122861746            8.6812115442

 H52             2.1997268134            -7.1914547282            9.5305924918

 H53             2.1602038380            -6.6985839248            7.8506187989

 C54             3.5749261949            -5.6500939141            8.9977857013

 N55             4.4340436395            -5.4872656240           10.0781072116

 H56             4.7029877491            -6.2069574392           10.7347901218

 C57             3.5419201462            -4.4212983703            8.3929554724

 H58             2.9623158060            -4.1137033865            7.5397813979

 C59             4.8863908156            -4.2144398190           10.0923648590

 H60             5.5598560558            -3.8006504485           10.8255903908

 N61             4.3669994401            -3.5443086607            9.0749155810

 N62             4.3811918402            -8.1168001443            5.9723138226

 H63             3.4936590772            -8.5919531501            5.8091609845

 C64             5.2069918374#           -7.7515171959#           4.8093070115#

 C65             6.5269258301            -8.5046640048            4.9622650858

 O66             7.6385605342            -8.0382003948            4.7797067322

 H67             6.4069353207            -9.5504010723            5.2985479675

 H68             4.6931180300            -8.1839544631            3.9453312270

 C69             5.3877823476            -6.2378633762            4.6043180675

 H70             6.0348139589            -6.1005732634            3.7333963977

 H71             5.9137461609            -5.8175354661            5.4624267959

 C72             4.0576521738            -5.5136653049            4.3853681497

 H73             3.6273006050            -5.7670884002            3.4131713489

 H74             3.3065185461            -5.8090882015            5.1309116338

 C75             4.1319570370            -4.0039145996            4.4468921143

 O76             3.1654831301            -3.3904194944            3.8779559314

 O77             5.0761809665            -3.4349344413            5.0726657981

 C78             9.1595116749#            0.1191580119#          11.4478228366#

 H79             9.9104939674            -0.3187599865           10.7747455622

 H80             9.5895510207             0.0193915959           12.4566787113

 C81             7.8218553915            -0.6281765875           11.3869856548

 H82             7.0560707813            -0.0941820028           11.9720475394

 H83             7.9203147192            -1.6122608031           11.8578139696

 C84             7.1861000054            -0.8472954418           10.0186004334

 O85             7.6257128365            -0.2346892632            8.9998655652

 O86             6.1950944697            -1.6483557490           10.0007584870

 C87             9.1035258385             1.5969792872           11.1622332741

 O88             8.0930986488             2.2514031046           11.0115867807

 H89            10.0937043794             2.0982958463           11.1171385653

 C90             7.7935565129#            4.3107436925#           8.7015790768#

 H91             8.2518459179             5.2981343499            8.8165777345

 H92             7.0584207389             4.1738393587            9.5014262671

 H93             8.5630967740             3.5468695635            8.8133109049

 C94             7.0955290187             4.1957335435            7.3355264708

 H95             6.3671938415             4.9996778464            7.1932132856

 H96             7.8393544742             4.2739538988            6.5304348083

 C97             6.3589033563             2.8735134282            7.1411074110

 O98             5.1667764200             2.8443086734            6.7827093212

 O99             7.0950191103             1.8213675506            7.3549009521

 C100           11.5816618274#           -0.6170833859#           3.4808991532#

 H101           12.2845265646            -0.9825883759            4.2392434026

 H102           12.0249338867             0.2735807281            3.0218910764

 H103           11.4919565705            -1.3864044844            2.7058235585

 C104           10.2193992093            -0.2997272848            4.0964549295

 H105            9.5425787431             0.0760081010            3.3195190408

 H106           10.3098538292             0.4866707842            4.8552763968

 C107            9.5950949630            -1.5523717273            4.7393063253

 H108           10.2381369422            -1.8981116978            5.5575294882

 H109            9.5189970888            -2.3567658766            4.0030042576

 C110            8.2254393857            -1.2674783123            5.3147324538

 O111            7.2138536829            -1.7564123806            4.7133146080

 O112            8.1726210462            -0.5543355450            6.3650649947

 C113           10.8187241310#            4.2285681323#          -0.8150213199#

 H114           11.5504498774             3.5653954119           -0.3392388622

 H115           11.1453562615             5.2620063190           -0.6511806017

 H116           10.8434116702             4.0361285331           -1.8923062597

 C117            9.4097538777             3.9992399754           -0.2479448311

 H118            9.1029304443             2.9687745739           -0.4821737912

 H119            8.6946382987             4.6560431571           -0.7626127969

 C120            9.3257288733             4.2496258692            1.2674011147

 H121            9.6758219911             5.2683451901            1.4799554277

 H122           10.0026174013             3.5689988502            1.8005649557

 C123            7.9167153016             4.1220045678            1.8683796094

 H124            7.2295205920             4.8177783664            1.3665392298

 H125            7.9418782371             4.3834888300            2.9304494497

 N126            7.4048980056             2.7527201134            1.7630832612

 H127            7.5583876869             2.2668933392            0.8890379368

 C128            6.7002535792             2.0724377612            2.6868776285

 N129            6.1435669292             2.6953288315            3.7413867331

 H130            5.7075393870             2.1273630802            4.4689778605

 H131            5.8246940201             3.6502822852            3.6647175537

 N132            6.5824112843             0.7549703272            2.5756580821

 H133            7.2262193315             0.1774067056            2.0349600866

 H134            5.8585296036             0.2317298887            3.0865398906

 C135           11.8423976378#           -5.3930895524#           7.2492549427#

 H136           11.3587989565            -6.3272875468            6.9446021429

 H137           12.8887184225            -5.6220149384            7.4751337339

 H138           11.8192431735            -4.7053633878            6.3977288692

 C139           11.1500490444            -4.7790245188            8.4751345005

 H140           11.6680206333            -3.8672977601            8.7916879264

 H141           11.2057496756            -5.4762121766            9.3212110191

 C142            9.7209985280            -4.4408209772            8.1992335375

 N143            8.7693981146            -5.4182493794            7.9473744837

 C144            9.0632639210            -3.2429959233            8.1013759517

 H145            9.4070273998            -2.2271353297            8.2132169675

 C146            7.5914514711            -4.8376233340            7.7009266178

 H147            6.6782852606            -5.3715528042            7.4840894304

 N148            7.7466042497            -3.5199667851            7.7878238178

 H149            6.9890908282            -2.8241293387            7.5902184123

 C150            6.0989358757#           -4.2351883009#          -1.2221974579#

 H151            5.5363340626            -4.7645315650           -2.0061492011

 C152            7.5233420046#           -4.0871570148#          -1.7388096288#

 H153            7.5881140624            -3.7439224678           -2.7937228495

 O154            8.5481992256            -4.2892232923           -1.1176143688

 H155            5.6758491427            -3.2210143573           -1.2272706147

 C156            5.9217361448            -4.9142485754            0.1278865069

 H157            6.2184552099            -5.9615871741            0.0665307746

 H158            6.5744427095            -4.4466119807            0.8740693483

 C159            4.4731451347            -4.8180040373            0.6237483972

 O160            3.8929044005            -3.6907834507            0.4913700962

 O161            3.9392376392            -5.8377643655            1.1415358310

 C162           -1.8288749435#          -11.9867170064#           4.2485681885#

 H163           -1.4112495691           -12.9782151750            4.0485251031

 H164           -2.8785580567           -12.0925410925            4.5528266260

 C165           -1.0564634105           -11.3343880344            5.3658287907

 H166           -1.4875915496           -10.3794258280            5.7387906763

 O167            0.0051073166           -11.7368979655            5.8087763549

 C168           -1.7390883701           -11.0751412091            2.9973573171

 H169           -2.4269812302           -11.4606446726            2.2348747352

 H170           -2.1069058211           -10.0732216004            3.2617701272

 C171           -0.3125110346           -11.0062987616            2.4319716941

 H172            0.3982791434           -10.7057987103            3.2098900000

 H173           -0.0062195512           -12.0192867204            2.1405562445

 C174           -0.1386518570           -10.0893488275            1.2121041934

 H175            0.8601739711           -10.2561157459            0.7913382271

 H176           -0.8550944030           -10.3712642915            0.4286852051

 C177           -0.3107632865            -8.5868472394            1.4549679706

 H178           -0.2201477966            -8.0473270131            0.5085069977

 H179           -1.2944319766            -8.3469635219            1.8687752004

 N180            0.7227548014            -7.9955179635            2.3714645624

 H181            0.9715902190            -7.0089641374            2.0226980019

 H182            0.4096435594            -7.8700335469            3.3456493020

 H183            1.5946806416            -8.5759257708            2.4409993431

 Cl184           0.7595256267            -6.7726025107            5.5093465494

 O185            1.2040803568            -1.7026117970            4.3135004441

 O186            4.8353252983            -1.2083253035            3.4885334517

 O187            0.9915236602             0.4515138477            6.4117248896

 H188            0.2097805373             0.8651236436            5.9453382603

 H189            0.9556315840            -0.4777423037            6.1141160741

 O190            2.9003373210             2.9956094033            8.5192992416

 H191            3.7616137524             3.4278107843            8.6620032435

 H192            2.7878881430             2.4312976329            9.3089439732

 O193            5.3178226506             2.3601556398           10.1545360030

 H194            5.2785455555             1.5638967904            9.5734870960

 H195            6.2712704309             2.4919332084           10.3240434265

 O196            2.5809462201            -1.8815778850            1.8245361248

 H197            2.8469441329            -2.7415272690            1.3896689579

 O198            3.0822495202            -1.5335308600            7.8376071211

 H199            2.0076952329            -1.3233326773            1.2466903202

 H200            0.9158267891            -2.6392807399            4.1567537095

 H201            3.1895924917            -1.7823662925            6.8819701633

 H202            8.9045290725            -6.4551707173            7.8389187604

 O203            0.0799866718             2.3754475407            9.1246531471

 H204            0.5998251240             1.5474226963            9.0936171633

 H205            0.7137920306             3.0522908468            8.8307048969

 O206           -1.2691545772             1.3843198461            5.3662433012

 H207           -1.6899046421             1.8806223190            6.1174474773

 H208           -1.4501408299             1.9039339216            4.5640905188

 O209           -2.1769386700             2.6617923104            7.5692570422

 H210           -2.8858851722             2.1734870815            8.0139473458

 H211           -1.3851789861             2.5389368478            8.1474940631

 O212            9.1281722310            -4.2203624146            1.6925564347

 H213            8.6416115078            -3.3692128826            1.7705729465

 H214            9.1546669735            -4.3655680155            0.7275946947

 O215            1.2083296894            -5.5487635413            1.4438690745

 H216            2.1911854152            -5.5070960986            1.3211241321

 H217            0.9750900196            -4.9462861910            2.1837317573

 O218            0.2242772627            -4.2402790956            3.7561916939

 H219            0.4010210366            -4.9827514847            4.3880151570

 H220           -0.7434340264            -4.1753107266            3.7138943029

 O221            5.2951138291            -1.4336864607            0.6947001039

 H222            4.8093327540            -2.2769453733            0.4651379828

 H223            4.9375368658            -1.2427415334            1.5887086085

 O224            7.7126219955            -1.7981205537            1.8938377130

 H225            6.8923677514            -1.8202973702            1.3370128953

 H226            7.3890015017            -1.9144135102            2.8113810765

 O227            8.6042651213            -8.0821226450            7.5245302181

 H228            7.6342166396            -8.0394934936            7.6569754339

 H229            8.6871566655            -8.2325376034            6.5638661107

 O230            2.7630014832            -9.6738673520            3.0687744033

 H231            2.5502227086            -9.8440497064            4.0110356972

 H232            3.6754388745           -10.0191900013            2.8945966990

 O233            5.7663798588            -8.0083905574            1.3470343965

 H234            5.0432430273            -7.3434212508            1.2886974992

 H235            6.5593785161            -7.5002908892            1.6444633525

 O236            7.8795344288            -6.5657239202            2.3354463804

 H237            8.1015023691            -6.8439924199            3.2379432531

 H238            8.3266638350            -5.6988086163            2.1623605126

 O239            1.9122688006            -9.6634917718            5.7466949517

 H240            1.3469998523            -8.8574797407            5.7471444153

 H241            1.3493975540           -10.4177000661            6.0204334949

 O242            5.3881039856           -10.2308597273            2.7383930148

 H243            5.6931579218           -10.9958045106            2.2273845652

 H244            5.5634779599            -9.4318985831            2.1563098533

 O245            5.1507325335             1.0370067319           12.6528560297

 H246            5.1100134946             1.5341233977           11.8059671517

 H247            5.2870037994             1.7233105600           13.3237246621

247 atom structure with an oxyl radical

 Mn1             2.9778054330            -1.3791931258            3.6936831447

 Mn2             4.5621465468            -1.4945147636            8.5954226291

 Mn3             5.2417304401            -1.4629182853            5.2986942839

 Mn4             6.5029012937             0.0651819476            7.2989000327

 O5              3.5295669985            -1.1931340922            5.6297876659

 O6              5.6379892001             0.2996392765            5.7279025180

 O7              5.0221250438             0.2916138840            8.3196457628

 O8              5.8399153545            -1.7253629000            7.1740644442

 Ca9             3.3945120570             1.1305338028            6.6928202619

 C10             2.1876270182#            1.2855489510#          -1.5249169529#

 C11             1.3910422428             0.9154796857           -0.2786264671

 O12             1.0834564494            -0.2558748412           -0.0343408146

 C13             3.4959732821             0.4850852971           -1.5625094241

 O14             4.3158169794             0.8616192581           -0.4688806867

 H15             1.5780517836             1.0333841001           -2.4013429793

 H16             3.2505665748            -0.5837993401           -1.5250106272

 H17             4.0044093059             0.6825752713           -2.5192143187

 H18             4.6978686437             0.0430178087           -0.0835608940

 H19             2.4058235102             2.3591828986           -1.5625877873

 N20             1.0185706451             1.9368605717            0.5487751860

 C21             0.3765073775             1.6907861317            1.8331308022

 C22            -1.1292185636             1.9395306995            1.7907451574

 O23            -1.8514436241             1.7952671881            2.7582906845

 C24             1.0557623872             2.4262014205            3.0028005170

 C25             2.1973906560             1.6458260031            3.6700306734

 O26             2.7990893381             2.1879119586            4.6220540531

 O27             2.3955840355             0.4700236284            3.2182322902

 H28             1.3735671148             2.8631081448            0.3522196861

 H29             0.4777008633             0.6133361015            2.0090828775

 H30             0.3187060204             2.6068911469            3.7935708519

 H31             1.4267536673             3.4140019323            2.7044527398

 H32            -1.5530311108             2.2227475442            0.8064340727

 C33             1.0582598435#            1.1829963843#          13.4563130443#

 H34             0.1327222459             0.5982899576           13.5299162563

 H35             0.8295656156             2.2013407377           13.7907622544

 H36             1.7808409272             0.7544896774           14.1609103235

 C37             1.6101912124             1.1721366263           12.0404799217

 H38             2.5406404437             1.7517361071           12.0035808441

 H39             0.9048117046             1.6431893088           11.3479322107

 C40             1.9077851384            -0.2627276016           11.5935260433

 H41             0.9731915952            -0.8394773612           11.5258102101

 H42             2.5584999736            -0.7623606213           12.3146061056

 C43             2.5742789742            -0.3024388023           10.2385577268

 O44             2.1953460340             0.4400098301            9.3060058633

 O45             3.5564697511            -1.1421718815           10.1590082622

 C46             3.8399778254            -8.1520209099            8.3710417118

 H47             3.2071500458            -9.0057123754            8.1085746614

 H48             4.4168663182            -8.4145743302            9.2637151313

 C49             4.8430754380            -7.8795741754            7.2594734294

 O50             5.9809356392            -7.4359909099            7.5204851654

 C51             2.9026669482            -6.9432987129            8.6519305984

 H52             2.2546994515            -7.2192556031            9.4951134833

 H53             2.2392525974            -6.7942065624            7.7957166896

 C54             3.5789459165            -5.6403671843            8.9429491654

 N55             4.3772525276            -5.4209428000           10.0604847086

 H56             4.6112196054            -6.1064710037           10.7653136071

 C57             3.5583892299            -4.4344783038            8.2888084537

 H58             3.0324238016            -4.1510007260            7.3929065762

 C59             4.8090970831            -4.1414216849           10.0495813515

 H60             5.4317735652            -3.6841294159           10.8018292652

 N61             4.3326078760            -3.5208550578            8.9815555952

 N62             4.4188936483            -8.1038257823            6.0026153936

 H63             3.5167510385            -8.5632399998            5.8778872339

 C64             5.2069918105#           -7.7515172166#           4.8093070180#

 C65             6.5313454888            -8.5058434181            4.9284600490

 O66             7.6394728218            -8.0341305334            4.7423565863

 H67             6.4174336132            -9.5583243749            5.2463225224

 H68             4.6649669488            -8.1857317065            3.9644380956

 C69             5.3848354614            -6.2367043497            4.6057772357

 H70             6.0099391643            -6.0925946018            3.7200817054

 H71             5.9349098669            -5.8229950395            5.4521766360

 C72             4.0486062841            -5.5133799030            4.4328576806

 H73             3.5743739716            -5.7861044817            3.4872274748

 H74             3.3386460563            -5.8002634802            5.2197242053

 C75             4.1094820248            -3.9990609429            4.4634788448

 O76             3.1715860463            -3.3906720206            3.8656009906

 O77             5.0546256085            -3.4262270232            5.1048448108

 C78             9.1595116494#            0.1191580088#          11.4478228207#

 H79             9.8806705806            -0.1547597189           10.6647894405

 H80             9.6734787583            -0.0795231154           12.4004443909

 C81             7.8676703417            -0.7121631200           11.3668540815

 H82             7.0984152981            -0.2949342247           12.0333272113

 H83             8.0562076127            -1.7291897210           11.7292051535

 C84             7.1722718231            -0.8543374479           10.0090295691

 O85             7.6142502445            -0.2075780792            9.0016916776

 O86             6.1672901389            -1.6230011405            9.9793670958

 C87             8.9594603720             1.6138205265           11.3722557095

 O88             7.8879555978             2.1831147184           11.3151267089

 H89             9.8955409230             2.2107233604           11.3959406534

 C90             7.7935564932#            4.3107436714#           8.7015790532#

 H91             8.3078788748             5.2710502452            8.8129991117

 H92             7.0719409910             4.2178115386            9.5180648025

 H93             8.5259017712             3.5078992476            8.8056569265

 C94             7.0961088910             4.2336986458            7.3384761947

 H95             6.3811219848             5.0509383392            7.2061163008

 H96             7.8427511628             4.3053673903            6.5357266700

 C97             6.3472086177             2.9240904457            7.1527334399

 O98             5.1450607688             2.9139589968            6.8265213319

 O99             7.0765996893             1.8701575450            7.3652377972

 C100           11.5816618434#           -0.6170833821#           3.4808991501#

 H101           12.2822545896            -1.0154676304            4.2246167231

 H102           12.0420571250             0.2725235867            3.0368441632

 H103           11.4665619210            -1.3673748609            2.6907571139

 C104           10.2322595715            -0.2811409577            4.1151164244

 H105            9.5586759365             0.1238849209            3.3501106865

 H106           10.3459811942             0.4897007644            4.8865808969

 C107            9.5846756718            -1.5294440246            4.7417847728

 H108           10.2179252496            -1.8932655777            5.5601864080

 H109            9.5021242558            -2.3267960595            3.9989160155

 C110            8.2155875345            -1.2332955113            5.3176828976

 O111            7.1996270855            -1.7351206984            4.7333684629

 O112            8.1781349093            -0.4982227527            6.3549213869

 C113           10.8187241289#            4.2285681278#          -0.8150213130#

 H114           11.5482485010             3.5355477135           -0.3802821680

 H115           11.1736804604             5.2491995699           -0.6313282872

 H116           10.8071714330             4.0673239777           -1.8976053344

 C117            9.4214028884             4.0143796158           -0.2130573481

 H118            9.0846005052             2.9976207182           -0.4653822301

 H119            8.7069860121             4.7003361473           -0.6893627884

 C120            9.3851105339             4.2263481544            1.3102495997

 H121            9.7631845876             5.2315327115            1.5386987834

 H122           10.0614821803             3.5178672609            1.8065293513

 C123            7.9910385515             4.1145716894            1.9484350468

 H124            7.3048431684             4.8340645622            1.4796369010

 H125            8.0511285623             4.3551567708            3.0141146818

 N126            7.4468784855             2.7582154355            1.8347095010

 H127            7.5767730220             2.2776258777            0.9538800624

 C128            6.7389955530             2.0864402932            2.7613808189

 N129            6.2135601519             2.7094225922            3.8310795024

 H130            5.8058075551             2.1311781688            4.5655816082

 H131            5.8991001536             3.6672154931            3.7731767610

 N132            6.5846183390             0.7735317966            2.6415456459

 H133            7.2197457005             0.1759017050            2.1089306053

 H134            5.8437506638             0.2818412738            3.1534694390

 C135           11.8423976356#           -5.3930895594#           7.2492549451#

 H136           11.3728889714            -6.3597392495            7.0381070655

 H137           12.9028994611            -5.5788792346            7.4468783317

 H138           11.7659134376            -4.7738217045            6.3495944143

 C139           11.1850006900            -4.7021167432            8.4532940136

 H140           11.6977104383            -3.7607668420            8.6785295669

 H141           11.2862737113            -5.3338948470            9.3455225163

 C142            9.7421603084            -4.4013521334            8.2056916455

 N143            8.8063683155            -5.3962753771            7.9575252134

 C144            9.0629937090            -3.2156562943            8.1086514129

 H145            9.3898315163            -2.1936622351            8.2164031046

 C146            7.6173726121            -4.8357828680            7.7170651337

 H147            6.7140677921            -5.3862726576            7.5013861494

 N148            7.7495497335            -3.5158632970            7.8038427224

 H149            6.9875901734            -2.8286313947            7.5953243428

 C150            6.0989358188#           -4.2351882676#          -1.2221974241#

 H151            5.5463694164            -4.7921598205           -1.9945189154

 C152            7.5233420092#           -4.0871570437#          -1.7388096191#

 H153            7.5891643286            -3.7627134493           -2.7996710499

 O154            8.5476910545            -4.2703299207           -1.1105369382

 H155            5.6611689493            -3.2283066758           -1.2582887246

 C156            5.9194711828            -4.8820715257            0.1427823661

 H157            6.2354667983            -5.9246974434            0.1112721347

 H158            6.5520397662            -4.3845189973            0.8867509596

 C159            4.4576609765            -4.7942055351            0.6000389317

 O160            3.8654320011            -3.6851302102            0.3964781904

 O161            3.9207015489            -5.8037465225            1.1341722776

 C162           -1.8288748610#          -11.9867169224#           4.2485681779#

 H163           -1.3696966156           -12.9574230138            4.0373392119

 H164           -2.8739430800           -12.1393692985            4.5483422449

 C165           -1.0876922666           -11.3164030938            5.3756986777

 H166           -1.5768919917           -10.4051698883            5.7846446326

 O167            0.0037968987           -11.6607232913            5.7933987807

 C168           -1.7752824756           -11.0612581160            3.0036809447

 H169           -2.4661085743           -11.4539136962            2.2475959115

 H170           -2.1612528936           -10.0699035358            3.2829264469

 C171           -0.3589292572           -10.9557513035            2.4181023320

 H172            0.3634161220           -10.6793762033            3.1938553025

 H173           -0.0490009416           -11.9544673350            2.0839919906

 C174           -0.2124301743            -9.9952228657            1.2271902387

 H175            0.7756165517           -10.1492600959            0.7769996574

 H176           -0.9474173139           -10.2488219569            0.4511801963

 C177           -0.3743603134            -8.5005482274            1.5227031576

 H178           -0.3066664016            -7.9326406998            0.5909911937

 H179           -1.3466510151            -8.2728571393            1.9694308522

 N180            0.6836760153            -7.9418579151            2.4316273440

 H181            0.9541029469            -6.9562071217            2.0993977402

 H182            0.3799869177            -7.8195387593            3.4091260747

 H183            1.5434725938            -8.5449848080            2.4915377813

 Cl184           0.6852749320            -6.7669206582            5.4896223753

 O185            0.9831563303            -1.7005873795            4.0256691554

 O186            4.7719144416            -1.1725076523            3.5431679352

 O187            1.1338282828             0.3049258381            6.5472811819

 H188            0.2710895564             0.4357775336            6.0613925071

 H189            1.0987830763            -0.5217955852            7.0539845783

 O190            2.7562429304             2.8832226043            8.4052996623

 H191            3.5592226116             3.3988451972            8.5923217965

 H192            2.6664362632             2.2716540733            9.1646999283

 O193            5.1160825166             2.1233974803           10.3822805119

 H194            5.1382637251             1.4232116981            9.6891827969

 H195            6.0529357654             2.2621107787           10.6167403205

 O196            2.6361015401            -1.7837393561            1.6579307266

 H197            2.8895451575            -2.6495646321            1.2375780465

 O198            3.1804091837            -1.5068778603            7.6363444532

 H199            2.0060071095            -1.2964223985            1.0849333769

 H200            0.7330634880            -2.6657593450            4.1283693576

 H201            0.5747207239            -1.1668096163            4.7302350279

 H202            8.9566016348            -6.4286446997            7.8392778919

 O203           -0.0932821989             2.1695932785            8.9751553193

 H204            0.5188076034             1.4077304688            9.0304085746

 H205            0.4821685124             2.8844613012            8.6527290382

 O206           -1.1836965373             0.4588340350            5.3053558860

 H207           -1.7025395189             0.9273959144            6.0189045985

 H208           -1.3800138070             0.9395706574            4.4769785316

 O209           -2.2847428659             1.7891736832            7.3567021833

 H210           -2.9324845325             1.2967540676            7.8824287088

 H211           -1.5142854293             1.9363931975            7.9629076991

 O212            9.1457124261            -4.1950940321            1.6952834579

 H213            8.6893280102            -3.3289528244            1.7809356397

 H214            9.1593601929            -4.3403788420            0.7298639251

 O215            1.2149865558            -5.4844979141            1.5447580903

 H216            2.1945925918            -5.4347224325            1.3924483561

 H217            0.9954562940            -4.8613893459            2.2670183804

 O218            0.1324690173            -4.2011767774            3.8948876755

 H219            0.3631451086            -4.9629968146            4.4878048419

 H220           -0.8316199077            -4.2420601666            3.7904950576

 O221            5.3675676526            -1.4704973775            0.7259931896

 H222            4.8558158743            -2.2805340221            0.4500444771

 H223            4.9717211808            -1.2866395068            1.6042813673

 O224            7.7795436934            -1.7313837137            1.9345669891

 H225            6.9751754799            -1.8037590327            1.3597435676

 H226            7.4408045810            -1.8712517597            2.8443528807

 O227            8.7052040736            -8.0706359772            7.4865725472

 H228            7.7339951827            -8.0643209827            7.6236198693

 H229            8.7816256132            -8.2088952491            6.5238252399

 O230            2.6890353305            -9.6585326368            3.0992989148

 H231            2.5058006508            -9.8085952675            4.0514524242

 H232            3.6018689442            -9.9955407494            2.9073490277

 O233            5.7236627123            -7.9799963165            1.3501374323

 H234            5.0088699654            -7.3047563818            1.2953163472

 H235            6.5263640397            -7.4801118089            1.6338230443

 O236            7.8575614856            -6.5377495361            2.3046360777

 H237            8.0792389725            -6.8370284820            3.2007861751

 H238            8.3105480275            -5.6706325203            2.1514555757

 O239            1.9233660475            -9.5913451019            5.8047879768

 H240            1.3555406981            -8.7868052601            5.7990297647

 H241            1.3536553194           -10.3441804662            6.0640705351

 O242            5.3027156356           -10.2112272338            2.7052074843

 H243            5.5934454000           -10.9733937362            2.1821068351

 H244            5.4911849084            -9.4050644965            2.1364809194

 O245            5.0144744490             0.5776750439           12.7784840791

 H246            4.9376961003             1.1040136346           11.9522843409

 H247            5.1368857704             1.2453731960           13.4705117546

261 atom structure with an OH

 Mn1             2.8980166637            -1.4739151107            3.7876854581

 Mn2             4.5391841384            -1.5242858552            8.7414569182

 Mn3             5.2575723405            -1.4348113876            5.2255216373

 Mn4             6.3921132993             0.0584030860            7.3249801377

 O5              3.4732327656            -1.1691515345            5.4816974837

 O6              5.5983901822             0.2762174332            5.7369301319

 O7              4.8828109842             0.2391384485            8.3224603659

 O8              5.7707124303            -1.7411849374            7.2392162931

 Ca9             3.1212914303             1.0489135366            6.8576541191

 C10             2.1876270759#            1.2855489160#          -1.5249168320#

 C11             1.5317922296             1.0152821371           -0.1735214065

 O12             1.2388643809            -0.1289424853            0.1851526699

 C13             3.5237943808             0.5328820067           -1.5976497753

 O14             4.3893387554             1.0015251165           -0.5779813872

 H15             1.5129911760             0.9241550474           -2.3100253581

 H16             3.3247361391            -0.5409775900           -1.4882466655

 H17             3.9691630453             0.6937034301           -2.5916497903

 H18             4.7790071642             0.2184921247           -0.1297781585

 H19             2.3542290290             2.3569375905           -1.6855131863

 N20             1.2860643794             2.1083793154            0.6058814304

 C21             0.6984862944             2.0428012400            1.9393347030

 C22            -0.6418360316             2.7846692744            1.9356240277

 O23            -1.0749968884             3.4449624495            2.8552017236

 C24             1.6254266566             2.5314381526            3.0569200066

 C25             2.5913893233             1.5014825685            3.6393523760

 O26             3.3958666807             1.8770529252            4.5221211337

 O27             2.4717327994             0.3125541681            3.1819935123

 H28             1.6129151683             3.0056987689            0.2726928010

 H29             0.4628441215             0.9824003499            2.1017135365

 H30             1.0070737416             2.8643102051            3.8981510660

 H31             2.2046508773             3.4086265269            2.7440284226

 H32            -1.2177438968             2.6701346547            0.9935226300

 C33             1.0582598535#            1.1829963901#          13.4563130168#

 H34            -0.0048228663             0.9350624443           13.3471601304

 H35             1.1230961357             2.1646394799           13.9397070698

 H36             1.5061956758             0.4496208525           14.1366339456

 C37             1.7734608994             1.1795791902           12.1115331708

 H38             2.8330818124             1.4119568990           12.2555948240

 H39             1.3586357297             1.9515582403           11.4518410716

 C40             1.6422100288            -0.1906252149           11.4254575626

 H41             0.5900788852            -0.3765210014           11.1779780050

 H42             1.9930206210            -0.9819573630           12.0924089057

 C43             2.4411139089            -0.2336464707           10.1423429741

 O44             2.1207654254             0.4733909299            9.1640176768

 O45             3.4736352537            -1.0251314375           10.2032038580

 C46             4.1119146680            -8.2173469912            8.4647547108

 H47             3.4860828483            -9.0856762494            8.2345527890

 H48             4.7755183673            -8.4850613909            9.2933972322

 C49             5.0066234679            -7.8868019752            7.2784640226

 O50             6.1471898957            -7.4088473038            7.4539894608

 C51             3.1615934535            -7.0562649348            8.8749675941

 H52             2.5925840391            -7.3953259908            9.7512534336

 H53             2.4275471945            -6.8946820175            8.0800084604

 C54             3.8007657959            -5.7340405292            9.1741588615

 N55             4.6384523859            -5.4884634329           10.2583194201

 H56             4.9248920336            -6.1640062063           10.9536151661

 C57             3.6815221958            -4.5195435620            8.5500938988

 H58             3.0805188567            -4.2549129796            7.6968553478

 C59             4.9932916822            -4.1833671312           10.2526289019

 H60             5.6246682000            -3.7079611647           10.9859593560

 N61             4.4324136889            -3.5713454990            9.2212982557

 N62             4.4964977897            -8.1087796876            6.0531578911

 H63             3.6097439551            -8.6132136509            5.9891060256

 C64             5.2069915225#           -7.7515171810#           4.8093069692#

 C65             6.5416324267            -8.4987719286            4.8500770783

 O66             7.6380870928            -8.0165784828            4.6287556404

 H67             6.4457555168            -9.5601695487            5.1444675842

 H68             4.6179412547            -8.1933235676            4.0012312477

 C69             5.3642329715            -6.2360147946            4.5957274165

 H70             5.9461577604            -6.0884567734            3.6820624977

 H71             5.9527432608            -5.8202377891            5.4148170248

 C72             4.0198654088            -5.5108885318            4.4862189162

 H73             3.4982362302            -5.7771269747            3.5649191357

 H74             3.3462840226            -5.7895705616            5.3082537542

 C75             4.1090767995            -3.9986040447            4.5160109856

 O76             3.1430994487            -3.3853453696            3.9423779360

 O77             5.0675028800            -3.4294246953            5.1163122526

 C78             9.1595115436#            0.1191579525#          11.4478228080#

 H79             9.8476185208            -0.1454529104           10.6319996833

 H80             9.7077413408            -0.1079543473           12.3746829167

 C81             7.8609602559            -0.6991667744           11.4006974513

 H82             7.1239062033            -0.3058729624           12.1176155197

 H83             8.0624540655            -1.7291478395           11.7180187777

 C84             7.1154114358            -0.7934265774           10.0699184047

 O85             7.5237080239            -0.1525952493            9.0544438826

 O86             6.0882898441            -1.5447114842           10.0765655455

 C87             8.9846006909             1.6179961979           11.4142973915

 O88             7.9231061444             2.2083261503           11.4033511883

 H89             9.9322395143             2.1962977331           11.4280968193

 C90             7.7935564680#            4.3107436657#           8.7015790121#

 H91             8.2389476331             5.3078380067            8.7844095702

 H92             7.2937070152             4.0818980841            9.6463583198

 H93             8.5955519014             3.5816581446            8.5600223402

 C94             6.8093164152             4.2682400809            7.5253286763

 H95             6.0276158699             5.0261918776            7.6347116183

 H96             7.3408375612             4.4880788049            6.5879356191

 C97             6.1178163706             2.9242783902            7.3275845353

 O98             4.9005415230             2.8748664258            7.0601080145

 O99             6.8994184134             1.8961707254            7.4333896866

 C100           11.5816617667#           -0.6170833920#           3.4808991652#

 H101           12.2970888903            -1.0026435489            4.2170228645

 H102           12.0353418111             0.2585373047            3.0041477949

 H103           11.4378470884            -1.3855917014            2.7134407791

 C104           10.2454425461            -0.2506469525            4.1412710019

 H105            9.5599947548             0.1359787872            3.3780406780

 H106           10.3855714486             0.5448289006            4.8831679652

 C107            9.5948929570            -1.4717765055            4.8247246306

 H108           10.2277680440            -1.7988497751            5.6583675821

 H109            9.5181794483            -2.2996484156            4.1146496050

 C110            8.2126628075            -1.1824494837            5.3860701248

 O111            7.2157035049            -1.7079470376            4.7822203254

 O112            8.1312259234            -0.4528845968            6.4210818984

 C113           10.8187240878#            4.2285681076#          -0.8150212978#

 H114           11.6046925039             3.6523393859           -0.3131834068

 H115           11.0957024607             5.2880148783           -0.7654396129

 H116           10.8088432910             3.9338552218           -1.8690832817

 C117            9.4492449793             3.9851030730           -0.1646110248

 H118            9.1898433564             2.9225019652           -0.2840668973

 H119            8.6768277790             4.5470141034           -0.7073787883

 C120            9.4141008264             4.3870537761            1.3200173760

 H121            9.6973081200             5.4432079333            1.4104072181

 H122           10.1628971954             3.8161238464            1.8852213793

 C123            8.0486967124             4.2242268928            2.0032366633

 H124            7.2867853763             4.7974327956            1.4529133775

 H125            8.1013513971             4.6216881097            3.0235943016

 N126            7.6693325452             2.8124517016            2.0829540053

 H127            8.0525321044             2.1943302146            1.3801384322

 C128            6.6677360425             2.3005989930            2.8270256396

 N129            5.9155523241             3.0929163564            3.6093566800

 H130            5.1306257030             2.6809963553            4.1240688335

 H131            5.8748184674             4.0839626348            3.4256910856

 N132            6.4503302475             0.9917811023            2.8039337643

 H133            7.1177554340             0.3281048522            2.3994503184

 H134            5.6882733981             0.5602611534            3.3300515968

 C135           11.8423975746#           -5.3930895638#           7.2492549400#

 H136           11.3775899867            -6.3806581478            7.1611633740

 H137           12.9109026261            -5.5486445997            7.4284831868

 H138           11.7295714005            -4.8744811533            6.2914961063

 C139           11.2218158064            -4.5806290925            8.3959633976

 H140           11.7407929759            -3.6225514207            8.5088653321

 H141           11.3547516971            -5.1192071594            9.3439579251

 C142            9.7697992164            -4.2995250658            8.1724880438

 N143            8.8464403661            -5.3061898144            7.9239861942

 C144            9.0648864500            -3.1255714032            8.1228785955

 H145            9.3763078259            -2.1004305075            8.2444076765

 C146            7.6416259583            -4.7627763861            7.7269636354

 H147            6.7457931162            -5.3252368890            7.5122592073

 N148            7.7486820773            -3.4431551338            7.8463470856

 H149            6.9636281079            -2.7694133065            7.6546197567

 C150            6.0989356464#           -4.2351883487#          -1.2221975107#

 H151            5.5557195198            -4.8457957281           -1.9587025617

 C152            7.5233420121#           -4.0871570053#          -1.7388094998#

 H153            7.5938150476            -3.8570290580           -2.8231592580

 O154            8.5444307384            -4.1744922386           -1.0845478364

 H155            5.6448627715            -3.2367891669           -1.3093278989

 C156            5.9363515263            -4.7939275120            0.1818960228

 H157            6.2291547319            -5.8423078530            0.2045525041

 H158            6.5991531507            -4.2646592686            0.8756795152

 C159            4.4959186395            -4.6415646340            0.6782373316

 O160            3.9530649996            -3.4992125381            0.4981509289

 O161            3.9316815093            -5.6240327412            1.2291036455

 C162           -1.8288745004#          -11.9867166181#           4.2485682547#

 H163           -1.3347361004           -12.8954958905            3.8909358288

 H164           -2.8285558068           -12.2427416637            4.6221195875

 C165           -1.0262376596           -11.3867127282            5.3737521826

 H166           -1.5504740687           -10.6001701030            5.9589677699

 O167            0.1374312564           -11.6532540427            5.6194409700

 C168           -1.9511271857           -10.9352523472            3.1132887178

 H169           -2.7286186000           -11.2656563691            2.4138304043

 H170           -2.3171813895            -9.9929774243            3.5463642159

 C171           -0.6270517236           -10.7182143199            2.3631411743

 H172            0.2082175731           -10.6061670513            3.0628124137

 H173           -0.4003522605           -11.6283109390            1.7928696308

 C174           -0.6354744579            -9.5293195494            1.3869576722

 H175            0.2530332588            -9.5851214710            0.7458001010

 H176           -1.5006766212            -9.6074817154            0.7146070126

 C177           -0.6876354316            -8.1381032657            2.0262771695

 H178           -0.8468166435            -7.3792153434            1.2559050880

 H179           -1.5127240762            -8.0563956047            2.7394261718

 N180            0.5766572312            -7.7506680821            2.7445718016

 H181            0.9429641438            -6.8386547726            2.3230581923

 H182            0.4293771200            -7.5391055424            3.7474251251

 H183            1.3374888180            -8.4763756312            2.7224964206

 Cl184           0.7900529281            -6.7265685939            5.8390035268

 O185            1.1911588554            -1.6278831588            4.3263874392

 O186            4.8166438594            -1.2213628079            3.5200998127

 O187            0.9593689688             0.5258021162            5.9969714168

 H188            0.1508272326             1.0285321469            5.7129793375

 H189            0.8993458968            -0.3333915397            5.5156569475

 O190            2.3646858745             3.3096779947            7.4924040071

 H191            3.2256355642             3.7709885817            7.4840014561

 H192            1.7159029511             3.8619504390            7.9465660910

 O193            5.1662779641             1.9456076096           10.5714829709

 H194            5.0448050409             1.3584636834            9.7956882897

 H195            6.1305207779             2.0547867893           10.6610096365

 O196            2.4396657199            -1.9392000353            1.8993800933

 H197            2.8820084898            -2.6771253825            1.3753043052

 O198            3.0340420726            -1.7876489751            7.8535727324

 H199            1.9964556496            -1.2751371542            1.3202793781

 H200            0.8762629225            -2.5668837827            4.2792715774

 H201            3.1551098219            -1.8293254215            6.8667451702

 H202            9.0176577234            -6.3306415312            7.7743818052

 O203           -0.5981808587             1.1933537204            9.3500194143

 H204            0.3244224501             0.9015504416            9.1653688289

 H205           -0.5136067178             2.1038734635            9.6663100479

 O206           -1.3295459534             1.6154635093            5.1564078856

 H207           -1.9169152794             1.4982485647            5.9467929453

 H208           -1.4725295533             2.5084420724            4.8073368477

 O209           -2.6306755645             1.1904078216            7.4958097260

 H210           -3.2014801909             0.4134677473            7.5839833805

 H211           -1.9104766493             1.0804128649            8.1629131167

 O212            9.0557746042            -4.0005154138            1.7347897449

 H213            8.6373741220            -3.1161985966            1.8126583093

 H214            9.0823869510            -4.1523652675            0.7698804857

 O215            1.2250194149            -5.3815085403            1.6874549876

 H216            2.1977124232            -5.3251451839            1.5092836337

 H217            1.0189957851            -4.7599666058            2.4172762585

 O218            0.1784749943            -4.1803716514            4.0549659860

 H219            0.4059001506            -4.8832184398            4.7092522567

 H220           -0.7888866787            -4.1126679520            4.0818783721

 O221            5.3458525989            -1.2490306348            0.7776669904

 H222            4.8883973368            -2.0999121841            0.5236116246

 H223            5.0160618190            -1.1065218187            1.6932558207

 O224            7.7495290047            -1.4775723209            2.0105619193

 H225            6.9563092043            -1.5326361602            1.4158837426

 H226            7.4103772234            -1.7260389011            2.8971165975

 O227            8.8636837133            -7.9666081516            7.3423515542

 H228            7.9012793616            -8.0240840563            7.5226331198

 H229            8.9016567614            -8.0737173996            6.3737830091

 O230            2.4661715884            -9.6913470707            3.1693585274

 H231            2.4020378983            -9.7837438733            4.1450250255

 H232            3.3884513417            -9.9480399469            2.9148736835

 O233            5.6519138364            -7.8708263260            1.4146402231

 H234            4.9645041385            -7.1685534021            1.3568559550

 H235            6.4806070686            -7.3969760253            1.6627282893

 O236            7.8777407768            -6.4529883820            2.2534619555

 H237            8.0836521019            -6.7611722372            3.1505720874

 H238            8.2571663886            -5.5462828499            2.1566568516

 O239            2.0252543579            -9.5895790216            5.9527509804

 H240            1.4492261860            -8.7921225213            5.9859602744

 H241            1.4446816666           -10.3622978156            6.1142059342

 O242            5.1076107730           -10.1470406274            2.6646016590

 H243            5.3551868463           -10.9047466794            2.1134946002

 H244            5.3373267222            -9.3342266122            2.1266746096

 O245            5.2218980003             0.5146902807           12.9804661534

 H246            5.0780087929             0.9275665414           12.0981433038

 H247            5.4494117024             1.2709916704           13.5431596803

 C248           -1.7760929738#           -3.1799649628#           9.5045649564#

 C249           -2.3274062240            -3.4402695578            8.0929974493

 C250           -1.6082270331            -2.5747995657            7.0484835500

 C251           -2.2456886656            -4.9268244087            7.7250145290

 H252           -3.3911659943            -3.1568199614            8.0958994673

 H253           -1.6879374595            -1.5012720886            7.2594492202

 H254           -2.0244784308            -2.7395965333            6.0458730944

 H255           -0.5396318917            -2.8189858013            7.0078484531

 H256           -2.6719177999            -5.1144477431            6.7314709900

 H257           -2.7991852290            -5.5419316549            8.4445555946

 H258           -1.2082236397            -5.2797773010            7.7042093840

 H259           -2.0586670334#           -2.1742880226#           9.8440010395#

 H260           -0.6822635067            -3.2623061010            9.5275834894

 H261           -2.1854411905            -3.8951822007           10.2279043653

261 atom structure with an oxyl radical

Mn1           2.8387602794     -1.4505723538      3.7720179889

  Mn2           4.5512503097     -1.4660400530      8.6374604412

  Mn3           5.1880878007     -1.4314065880      5.3204012984

  Mn4           6.4530372167      0.1037244780      7.3012568063

  O5            3.4939509580     -1.2169049625      5.7263112209

  O6            5.5747825467      0.3225818264      5.7437947568

  O7            4.9958805721      0.3203504371      8.3409396251

  O8            5.8293662897     -1.6932781497      7.1913345740

  Ca9           3.2681113486      1.0880339218      6.8647424365

  C10           2.1876270601      1.2855489251     -1.5249169752

  C11           1.3871947352      0.9516504456     -0.2770735497

  O12           1.0512463046     -0.2050102272     -0.0105040993

  C13           3.5202157452      0.5229516939     -1.5012780061

  O14           4.2815990031      0.9338474575     -0.3801334910

  H15           1.6016148139      0.9781235994     -2.3993417839

  H16           3.3054874599     -0.5529205618     -1.4646900489

  H17           4.0608016055      0.7264049178     -2.4390740806

  H18           4.6544124615      0.1279961046      0.0446121535

  H19           2.3778044173      2.3621464061     -1.6061746031

  N20           1.0498216599      2.0037922062      0.5286072781

  C21           0.3872070446      1.8086876616      1.8056528633

  C22          -1.0774511337      2.2324155440      1.7628586665

  O23          -1.8288994467      2.1107439155      2.7122427491

  C24           1.1379815323      2.4460413115      2.9912183320

  C25           2.1555355957      1.5291310700      3.6874297799

  O26           2.7857957438      1.9992679584      4.6526759653

  O27           2.2222087801      0.3358258780      3.2303307122

  H28           1.4417025613      2.9109118509      0.3144614872

  H29           0.3741974553      0.7230069233      1.9579643979

  H30           0.4216488832      2.7375826085      3.7673888759

  H31           1.6504811814      3.3675717654      2.6908145220

  H32          -1.4478589427      2.6359916889      0.7992833136

  C33           1.0582597414      1.1829963002     13.4563129117

  H34           0.1190029905      0.6200902598     13.5252932579

  H35           0.8700009179      2.1903359669     13.8462470927

  H36           1.7879695788      0.7004569406     14.1168941089

  C37           1.5795405458      1.2228655503     12.0261915173

  H38           2.5307314989      1.7665522747     11.9864350322

  H39           0.8776737599      1.7532318871     11.3723510713

  C40           1.7893132490     -0.2007820207     11.4983415461

  H41           0.8159824039     -0.6918894028     11.3575179086

  H42           2.3698002679     -0.7868419361     12.2144147376

  C43           2.5122751850     -0.2177859022     10.1706470384

  O44           2.1489480450      0.5047833830      9.2209231489

  O45           3.5256034908     -1.0333426410     10.1542182508

  C46           3.9314307821     -8.1132392702      8.4071134639

  H47           3.3185472515     -8.9909149340      8.1782634828

  H48           4.5332887751     -8.3397186754      9.2930321693

  C49           4.9044726029     -7.8444836640      7.2671454722

  O50           6.0501395304     -7.4009851782      7.4888495563

  C51           2.9662560927     -6.9269125044      8.6831669352

  H52           2.3132632407     -7.2211728340      9.5161958358

  H53           2.3097563903     -6.7810356661      7.8204720814

  C54           3.6149900019     -5.6137213217      8.9951947406

  N55           4.3635984440     -5.3784165790     10.1448842654

  H56           4.5581588924     -6.0497216380     10.8749882154

  C57           3.6080840498     -4.4121603055      8.3335470913

  H58           3.1100874275     -4.1344937238      7.4188838427

  C59           4.7771508626     -4.0920864184     10.1449566342

  H60           5.3610431381     -3.6222080870     10.9203858384

  N61           4.3379963728     -3.4850789658      9.0538729855

  N62           4.4424907427     -8.0789720470      6.0253809611

  H63           3.5347073804     -8.5417437149      5.9335322837

  C64           5.2069914248     -7.7515173135      4.8093069714

  C65           6.5232294600     -8.5242781999      4.9003075617

  O66           7.6323951683     -8.0740683070      4.6740502494

  H67           6.4016117860     -9.5720443164      5.2327093466

  H68           4.6431980928     -8.1881089971      3.9804922908

  C69           5.3958351531     -6.2392702593      4.5900676393

  H70           6.0156390759     -6.1078127736      3.6987218396

  H71           5.9540453491     -5.8227474412      5.4300000116

  C72           4.0593233281     -5.5145470957      4.4190327175

  H73           3.5993142458     -5.7636404002      3.4590493997

  H74           3.3381734915     -5.8234490803      5.1869478092

  C75           4.1002720281     -4.0005438937      4.4922841474

  O76           3.1198747778     -3.4061357731      3.9419500733

  O77           5.0524808091     -3.4193868607      5.1059491860

  C78           9.1595114348      0.1191579339     11.4478227393

  H79           9.8586932898     -0.1262306815     10.6353432636

  H80           9.7008737337     -0.1178919925     12.3764079158

  C81           7.8726115504     -0.7131173185     11.3681775254

  H82           7.1159307476     -0.3415839147     12.0742700634

  H83           8.0831480238     -1.7414804970     11.6847986757

  C84           7.1534062458     -0.8191655275     10.0205120842

  O85           7.5860920199     -0.1704404226      9.0117723819

  O86           6.1399778104     -1.5778927223     10.0079863939

  C87           8.9795810477      1.6170739200     11.4360175001

  O88           7.9205901418      2.2116385457     11.4093859685

  H89           9.9263471106      2.1954914493     11.4788557143

  C90           7.7935563838      4.3107436284      8.7015789879

  H91           8.2334186931      5.3021277712      8.8545861385

  H92           7.2265645049      4.0482236435      9.5987595192

  H93           8.6017125036      3.5832815975      8.5948194383

  C94           6.9003246800      4.3118819318      7.4597761103

  H95           6.1141058656      5.0702147914      7.5307130171

  H96           7.4973910108      4.5550761026      6.5682678054

  C97           6.2257164906      2.9744369939      7.1867602685

  O98           5.0484845214      2.9440239288      6.7623977456

  O99           6.9757055791      1.9421604489      7.3913976105

  C100         11.5816617591     -0.6170834019      3.4808992075

  H101         12.2675510072     -1.0357290764      4.2270870267

  H102         12.0647858389      0.2640565365      3.0444670365

  H103         11.4530390504     -1.3606148952      2.6865380925

  C104         10.2338785652     -0.2511726308      4.1101147863

  H105          9.5771304085      0.1755928169      3.3419136288

  H106         10.3634530920      0.5145899259      4.8842847413

  C107          9.5468590618     -1.4832495010      4.7293323563

  H108         10.1657575347     -1.8651771101      5.5505628640

  H109          9.4534919002     -2.2773543072      3.9840664062

  C110          8.1759646825     -1.1700849132      5.3024770756

  O111          7.1578633964     -1.6697118966      4.7188954618

  O112          8.1399681710     -0.4329481692      6.3384634967

  C113         10.8187240893      4.2285681031     -0.8150212740

  H114         11.5740407625      3.5512532218     -0.3999252399

  H115         11.1672604378      5.2560953284     -0.6589360609

  H116         10.7682293077      4.0523172106     -1.8941710872

  C117          9.4503114778      4.0049526820     -0.1566412718

  H118          9.1164746611      2.9815431000     -0.3845479105

  H119          8.7078985416      4.6760747901     -0.6104793734

  C120          9.4765438312      4.2359480905      1.3634541455

  H121          9.8543564838      5.2468529107      1.5636863762

  H122         10.1794799007      3.5400455522      1.8401287942

  C123          8.1124792455      4.1172582944      2.0577904495

  H124          7.4013738777      4.8252516521      1.6064184391

  H125          8.2159436898      4.3736958431      3.1174624259

  N126          7.5913876518      2.7507449273      1.9796107400

  H127          7.7843982529      2.2267000501      1.1361208816

  C128          6.7394039854      2.1697581508      2.8465749196

  N129          6.1452268135      2.8854063225      3.8165208806

  H130          5.6279980391      2.4087534917      4.5535323521

  H131          6.0117866993      3.8804952133      3.7236894814

  N132          6.5027714456      0.8680287476      2.7573446811

  H133          7.1320177780      0.2146902482      2.2792806870

  H134          5.7487130981      0.4334567839      3.2947684603

  C135         11.8423975422     -5.3930895717      7.2492549551

  H136         11.3732893083     -6.3761657834      7.1356720560

  H137         12.9090391959     -5.5576663660      7.4320734948

  H138         11.7376570275     -4.8532701808      6.3024306637

  C139         11.2165244774     -4.6034537256      8.4086393571

  H140         11.7357719944     -3.6481256069      8.5416769505

  H141         11.3436397747     -5.1597935403      9.3469397657

  C142          9.7667606197     -4.3157347447      8.1821155296

  N143          8.8298924065     -5.3180035920      7.9673808539

  C144          9.0773724388     -3.1349518044      8.0950265524

  H145          9.4004679867     -2.1102909978      8.1873641796

  C146          7.6314037885     -4.7652135012      7.7557133588

  H147          6.7270185238     -5.3214244558      7.5596027710

  N148          7.7574781212     -3.4441338100      7.8306987510

  H149          6.9859399879     -2.7621805763      7.6268187609

  C150          6.0989354702     -4.2351882909     -1.2221974475

  H151          5.5423206745     -4.7909269231     -1.9917421651

  C152          7.5233420644     -4.0871570650     -1.7388094015

  H153          7.5888081554     -3.8023576053     -2.8107654187

  O154          8.5487397458     -4.2277847548     -1.1007172496

  H155          5.6653456535     -3.2243240908     -1.2536487342

  C156          5.9122113808     -4.8670958852      0.1484247344

  H157          6.2078509072     -5.9156060370      0.1239671080

  H158          6.5576318336     -4.3728271221      0.8830264787

  C159          4.4551618772     -4.7494330069      0.6128171238

  O160          3.8955581412     -3.6134318226      0.4740975611

  O161          3.8893513559     -5.7695113443      1.0964490468

  C162         -1.8288743334    -11.9867164546      4.2485682643

  H163         -1.2873093686    -12.8887135430      3.9470191332

  H164         -2.8224413457    -12.2702756478      4.6191910718

  C165         -1.0787212818    -11.2942005081      5.3556813334

  H166         -1.6474081490    -10.4954817349      5.8803752533

  O167          0.0855259055    -11.5033035615      5.6482572040

  C168         -1.9791206980    -11.0008353327      3.0590257753

  H169         -2.7421801011    -11.3913823109      2.3750211812

  H170         -2.3772686044    -10.0491943314      3.4406425127

  C171         -0.6585066352    -10.7823652196      2.3027124089

  H172          0.1749982311    -10.6360745688      2.9978718111

  H173         -0.4160216623    -11.7047114440      1.7594365415

  C174         -0.6878012756     -9.6225382033      1.2919965090

  H175          0.1933158348     -9.6887631963      0.6418343595

  H176         -1.5602978451     -9.7270775458      0.6327187205

  C177         -0.7440554966     -8.2141599061      1.8932468061

  H178         -0.9393657345     -7.4799854576      1.1071601082

  H179         -1.5488428614     -8.1274348917      2.6291099642

  N180          0.5347789612     -7.7862438850      2.5611102565

  H181          0.8982082833     -6.9064526784      2.0718170530

  H182          0.3992577424     -7.5023216575      3.5470453904

  H183          1.2900487811     -8.5204256453      2.5797551957

  Cl184         0.6793754694     -6.4942484655      5.5765382894

  O185          0.9352504204     -1.8053255871      4.4263849398

  O186          4.6336110420     -1.1459881325      3.5852157492

  O187          0.9916231523      0.3034891751      6.6023408505

  H188          0.3804017447      0.4088418850      5.8396079638

  H189          1.0902469535     -0.6576588616      6.7219010198

  O190          2.6011947078      3.2955109998      7.7088067493

  H191          1.9275728083      3.8057119721      8.1762816559

  H192          3.3488301476      3.8788220792      7.4910653646

  O193          5.1437900720      1.8428450262     10.7312729916

  H194          5.0801053170      1.3233309010      9.9018983240

  H195          6.0986938334      1.9854173756     10.8700926937

  O196          2.1832457477     -2.0483471426      1.7867109727

  H197          2.6635015194     -2.7759238600      1.3103979488

  O198          3.1702930563     -1.5363080597      7.6806161344

  H199          1.8331197959     -1.4128233125      1.1276893506

  H200          0.5980723902     -2.6943256268      4.1098853167

  H201          0.2220581328     -1.1331715429      4.2924191741

  H202          8.9841180915     -6.3482039627      7.8462415873

  O203         -0.6883716642      0.9075389333      9.1767022254

  H204          0.2754649744      0.7354912636      9.0655435775

  H205         -0.7420754410      1.8511540871      9.3894709085

  O206         -0.9396654218      0.2024035510      4.6662655073

  H207         -1.5373216628      0.2119947955      5.4658957542

  H208         -1.2799408470      0.8992892359      4.0667175490

  O209         -2.2861710937      0.5705025707      6.9610181474

  H210         -3.0262823280      0.0237623248      7.2631394376

  H211         -1.6639740422      0.6265023424      7.7335192225

  O212          8.9985122520     -4.1249411784      1.7259418822

  H213          8.5706362176     -3.2448149143      1.8003107729

  H214          9.0524773461     -4.2625312135      0.7600265852

  O215          1.1773553991     -5.4934856030      1.3412781618

  H216          2.1635004105     -5.4696482785      1.2268125922

  H217          0.9548028427     -4.8192218471      2.0150286515

  O218          0.0271291113     -4.1722207651      3.6229027173

  H219          0.2951956353     -4.8517945141      4.2924703071

  H220         -0.9420482693     -4.2166341123      3.5961844853

  O221          5.2383990079     -1.3661635141      0.8660482673

  H222          4.7780661847     -2.2126700546      0.5915709952

  H223          4.9300654453     -1.2442054451      1.7941962580

  O224          7.6968074628     -1.6020609411      1.9554025971

  H225          6.8724738140     -1.6622811000      1.4044178703

  H226          7.3933726535     -1.7960300210      2.8696959620

  O227          8.7729445308     -7.9893277695      7.4354051308

  H228          7.8042714699     -8.0204112765      7.5858477401

  H229          8.8407237259     -8.1162221417      6.4710426647

  O230          2.3907021998     -9.7289228122      3.0760594087

  H231          2.3158253767     -9.7323020584      4.0546421609

  H232          3.3312978604     -9.9596925992      2.8635406622

  O233          5.6388740724     -7.9686705486      1.3625404669

  H234          4.9506132551     -7.2696572481      1.2742096887

  H235          6.4640376394     -7.4929083572      1.6217044528

  O236          7.8891985995     -6.6104549043      2.2165866617

  H237          8.0682857485     -6.8997353451      3.1261515600

  H238          8.2357069542     -5.6896191043      2.1295614047

  O239          1.9069088835     -9.3774939733      5.8286071121

  H240          1.3364406999     -8.5756907295      5.8175862498

  H241          1.3367478235    -10.1403809795      6.0587560424

  O242          5.0542301898    -10.1690123193      2.7038653603

  H243          5.3055872852    -10.9516519695      2.1904243991

  H244          5.3047972750     -9.3807007111      2.1353959673

  O245          4.9854168001      0.2014354361     12.9983168105

  H246          4.9290858144      0.6803745456     12.1396014465

  H247          5.0898220546      0.9158130359     13.6452420463

  C248         -1.7760929974     -3.1799649432      9.5045648512

  C249         -2.5653483990     -3.6268561693      8.2598922808

  C250         -1.9861029243     -3.0089281862      6.9795515996

  C251         -2.6039488358     -5.1568858476      8.1426093532

  H252         -3.6021480221     -3.2763135057      8.3745912323

  H253         -1.8951172094     -1.9193663460      7.0479579906

  H254         -2.6160940863     -3.2387768941      6.1099280439

  H255         -0.9850326618     -3.4126695378      6.7800033989

  H256         -3.1870017244     -5.4718886136      7.2684772082

  H257         -3.0590169937     -5.6113394815      9.0307540490

  H258         -1.5935765478     -5.5681248360      8.0265903284

  H259         -2.0586669969     -2.1742880286      9.8440010874

  H260         -0.6948865706     -3.1919055944      9.3151381621

  H261         -1.9685675140     -3.8547004244     10.3481906171

304 atom structure with an OH

 Mn1             2.8935076094            -1.4518054302            3.8069469056

 Mn2             4.5189904972            -1.5304313217            8.7455332389

 Mn3             5.2501248711            -1.4070968070            5.2472760647

 Mn4             6.3744266252             0.0749873830            7.3482895270

 O5              3.4595430222            -1.1401869656            5.5007817048

 O6              5.5896791760             0.3025920258            5.7563815007

 O7              4.8545069750             0.2378202742            8.3377803746

 O8              5.7605291954            -1.7310355198            7.2490282793

 Ca9             3.0833868557             1.1045422106            6.8716006775

 C10             2.1876270787#            1.2855489124#          -1.5249168564#

 C11             1.5419912337             0.9903406278           -0.1779644438

 O12             1.2012848038            -0.1503354871            0.1506510841

 C13             3.5158659800             0.5183863878           -1.6262405640

 O14             4.4036164278             0.9692034393           -0.6176591488

 H15             1.5021153391             0.9494884743           -2.3119921069

 H16             3.3065426881            -0.5540980486           -1.5212236060

 H17             3.9486648777             0.6814958160           -2.6252242071

 H18             4.7631723718             0.1809621981           -0.1524163780

 H19             2.3665862155             2.3576378853           -1.6632569107

 N20             1.3647152801             2.0729267458            0.6280146269

 C21             0.6835809478             2.0479660594            1.9193877672

 C22            -0.5667384368             2.9082779967            1.7127030976

 O23            -0.7586857381             3.9850638151            2.2320748847

 C24             1.5477681475             2.5289758844            3.0822924024

 C25             2.5330736443             1.5162874206            3.6638262098

 O26             3.3049207633             1.9022652040            4.5678769063

 O27             2.4572281857             0.3286914839            3.1884812693

 H28             1.7277833687             2.9616273245            0.3090849959

 H29             0.3760167553             1.0100825815            2.0786746295

 H30             0.8884755386             2.7931465452            3.9167078077

 H31             2.0930404077             3.4464215237            2.8342808867

 H32            -1.2791697675             2.4959928205            0.9682217594

 C33             1.0582598459#            1.1829964009#          13.4563130427#

 H34             0.0034907618             0.8862110737           13.4069613231

 H35             1.1096662039             2.1771813029           13.9099173407

 H36             1.5728238036             0.4845109097           14.1271975047

 C37             1.6986844366             1.1782113068           12.0733360298

 H38             2.7470919212             1.4853426038           12.1532373894

 H39             1.1921886189             1.8904236477           11.4131820323

 C40             1.6259713906            -0.2223988721           11.4425120037

 H41             0.5807010558            -0.4712516152           11.2189612542

 H42             2.0220773534            -0.9739513104           12.1305300652

 C43             2.4097043140            -0.2719343151           10.1491457781

 O44             2.0696161204             0.4134676598            9.1688957084

 O45             3.4587818679            -1.0504475449           10.2123374859

 C46             4.1359542201            -8.2286357669            8.4700096867

 H47             3.5212793752            -9.1050325943            8.2399121565

 H48             4.8019490726            -8.4874164844            9.2996848838

 C49             5.0270322644            -7.8866118048            7.2837164054

 O50             6.1643272451            -7.4014525829            7.4579469508

 C51             3.1718805784            -7.0771678762            8.8750443020

 H52             2.6039320438            -7.4202352515            9.7505950097

 H53             2.4401993776            -6.9256653647            8.0765111858

 C54             3.8010171922            -5.7515955750            9.1728711069

 N55             4.6432726593            -5.5051260316           10.2534753093

 H56             4.9382747739            -6.1817317532           10.9439309883

 C57             3.6741823326            -4.5368608860            8.5517848761

 H58             3.0701370309            -4.2729735430            7.7005332858

 C59             4.9941143337            -4.1984391099           10.2470212469

 H60             5.6282136435            -3.7212044026           10.9767645859

 N61             4.4257766281            -3.5876117298            9.2197631681

 N62             4.5101323120            -8.1060317952            6.0601846402

 H63             3.6304274328            -8.6213957911            6.0024297309

 C64             5.2069915804#           -7.7515171131#           4.8093069890#

 C65             6.5413108915            -8.5025752740            4.8390230726

 O66             7.6377270190            -8.0173155173            4.6238923296

 H67             6.4460699356            -9.5656405414            5.1260050264

 H68             4.6060418608            -8.1878916726            4.0068111985

 C69             5.3726633072            -6.2336997871            4.5974627124

 H70             5.9479567492            -6.0900747195            3.6789328293

 H71             5.9736437488            -5.8260493928            5.4116151222

 C72             4.0334329552            -5.4953729724            4.5025259222

 H73             3.4980302335            -5.7631745240            3.5896947088

 H74             3.3696062107            -5.7707578231            5.3333690484

 C75             4.1247497043            -3.9815022659            4.5291208265

 O76             3.1614242522            -3.3695898483            3.9490355054

 O77             5.0786805207            -3.4113867965            5.1351990198

 C78             9.1595115474#            0.1191579538#          11.4478228090#

 H79             9.8337894302            -0.1208024432           10.6135377429

 H80             9.7289919239            -0.1149048738           12.3601769946

 C81             7.8698376899            -0.7171666983           11.4091202584

 H82             7.1365407651            -0.3427263718           12.1389012347

 H83             8.0904099117            -1.7479865647           11.7108314457

 C84             7.1084019286            -0.8046126444           10.0839003391

 O85             7.5134681511            -0.1574484874            9.0707840968

 O86             6.0802669450            -1.5517668555           10.0949693287

 C87             8.9580102932             1.6151873783           11.4436296109

 O88             7.8863094001             2.1860272157           11.4863704964

 H89             9.8951129559             2.2105674751           11.4260956649

 C90             7.7935564894#            4.3107436776#           8.7015790283#

 H91             8.2163521156             5.3169282187            8.7965439587

 H92             7.3481420083             4.0372834094            9.6620048639

 H93             8.6065841853             3.6087565206            8.4977828922

 C94             6.7536723571             4.2780064666            7.5751565776

 H95             5.9525707260             5.0023414911            7.7521847903

 H96             7.2265341465             4.5574657068            6.6218244719

 C97             6.0874371652             2.9240332334            7.3429011917

 O98             4.8960380325             2.8640026640            6.9986881649

 O99             6.8772666965             1.9007634362            7.4991011613

 C100           11.5816617650#           -0.6170833909#           3.4808991637#

 H101           12.2960230394            -0.9821261390            4.2284554811

 H102           12.0314880231             0.2505061146            2.9862709792

 H103           11.4444123842            -1.4032708428            2.7302699774

 C104           10.2389444600            -0.2434850716            4.1278395743

 H105            9.5547993636             0.1213788939            3.3528042422

 H106           10.3725386266             0.5702520871            4.8511501654

 C107            9.5899979421            -1.4504999997            4.8391363423

 H108           10.2251748011            -1.7590376836            5.6777639293

 H109            9.5131672672            -2.2925589097            4.1455085487

 C110            8.2060625062            -1.1563894526            5.4000235197

 O111            7.2085672069            -1.6795419573            4.7937732083

 O112            8.1248544967            -0.4300560722            6.4369997287

 C113           10.8187241016#            4.2285681147#          -0.8150213088#

 H114           11.5889548779             3.6476341280           -0.2946402848

 H115           11.0976512174             5.2869537883           -0.7548501805

 H116           10.8346426866             3.9369244238           -1.8698598587

 C117            9.4297241360             3.9875907166           -0.2015414759

 H118            9.1692985906             2.9268172285           -0.3343886433

 H119            8.6757922048             4.5560279239           -0.7634229066

 C120            9.3492593379             4.3801308371            1.2849164892

 H121            9.6403193418             5.4329466216            1.3910510960

 H122           10.0720584420             3.7968868142            1.8707273673

 C123            7.9588343919             4.2306838819            1.9234718622

 H124            7.2226972092             4.8192427801            1.3549773609

 H125            7.9862982347             4.6218494912            2.9469128042

 N126            7.5532867083             2.8254741516            1.9902347221

 H127            7.8805707427             2.2197925800            1.2491645522

 C128            6.5903560358             2.3036507572            2.7808808559

 N129            5.8558816451             3.0855978921            3.5868513043

 H130            5.1363992000             2.6672171075            4.1820948137

 H131            5.8036549302             4.0796329419            3.4269773320

 N132            6.3934116561             0.9921834329            2.7641929102

 H133            7.0741588403             0.3449503893            2.3593814226

 H134            5.6457387503             0.5472666820            3.2997550434

 C135           11.8423975757#           -5.3930895535#           7.2492549391#

 H136           11.3789263907            -6.3836788440            7.1940130511

 H137           12.9114695750            -5.5410576261            7.4315804429

 H138           11.7271549942            -4.9058781854            6.2754151151

 C139           11.2229198690            -4.5447870840            8.3700129400

 H140           11.7419281026            -3.5834065666            8.4511035013

 H141           11.3595410302            -5.0529022682            9.3343903760

 C142            9.7697601410            -4.2696087028            8.1443197245

 N143            8.8472642206            -5.2793309611            7.9035359657

 C144            9.0609919948            -3.0978724471            8.0982762791

 H145            9.3698577134            -2.0716125332            8.2163071923

 C146            7.6397169186            -4.7382881782            7.7139752218

 H147            6.7439618316            -5.3022918136            7.5038576220

 N148            7.7441085886            -3.4184345317            7.8297203249

 H149            6.9530800452            -2.7458134814            7.6407939076

 C150            6.0989356639#           -4.2351883310#          -1.2221974936#

 H151            5.5588989303            -4.8547670670           -1.9536017420

 C152            7.5233420042#           -4.0871570086#          -1.7388095282#

 H153            7.5943214193            -3.8587386572           -2.8236049893

 O154            8.5443139720            -4.1766056518           -1.0847455030

 H155            5.6397013226            -3.2397263148           -1.3151837302

 C156            5.9403801835            -4.7859204863            0.1867764860

 H157            6.2578129897            -5.8267070520            0.2184520898

 H158            6.5883687865            -4.2363534216            0.8787439968

 C159            4.4944597174            -4.6631933700            0.6792168050

 O160            3.9283740751            -3.5330366158            0.5056173736

 O161            3.9502636011            -5.6599000009            1.2285113715

 C162           -1.8288746092#          -11.9867167117#           4.2485682405#

 H163           -1.3767193978           -12.9262962685            3.9158926024

 H164           -2.8455581532           -12.1825789330            4.6117173706

 C165           -1.0085898703           -11.4015979159            5.3689346447

 H166           -1.5081106283           -10.5970553003            5.9510722882

 O167            0.1485766689           -11.6972374458            5.6108141764

 C168           -1.8785344285           -10.9544928293            3.0904275018

 H169           -2.6526796221           -11.2671053030            2.3789143868

 H170           -2.2112925440            -9.9874105893            3.4943992051

 C171           -0.5299742847           -10.8158678156            2.3685271825

 H172            0.2875329132           -10.6797228000            3.0843420455

 H173           -0.3104714753           -11.7641356031            1.8610278100

 C174           -0.4780020883            -9.6909311341            1.3227095944

 H175            0.4413223428            -9.7971869053            0.7333584622

 H176           -1.3095474000            -9.8024389201            0.6134406488

 C177           -0.5409403228            -8.2598779738            1.8647938219

 H178           -0.6074788702            -7.5510456058            1.0353329379

 H179           -1.4210655888            -8.1047530112            2.4955089359

 N180            0.6691635879            -7.8572892415            2.6631144368

 H181            1.0195324199            -6.9143369347            2.2831011649

 H182            0.4738131787            -7.6882781944            3.6638347572

 H183            1.4493633227            -8.5617669660            2.6485101385

 Cl184           0.8469370634            -6.7853499505            5.7830604481

 O185            1.1866854438            -1.6336270930            4.3357103627

 O186            4.8089307502            -1.2019246632            3.5412820917

 O187            0.8966034710             0.5175554930            6.0356420873

 H188            0.0448259323             0.9770502106            5.8084610989

 H189            0.8632250328            -0.3336040317            5.5401668541

 O190            2.2341036259             3.2173665186            7.6731351015

 H191            2.8842329818             3.9195319604            7.8144206606

 H192            1.3588758192             3.5146791869            8.0076810462

 O193            5.1194198528             1.8993258914           10.6067777277

 H194            4.9959236218             1.3167885902            9.8267470698

 H195            6.0821107785             2.0237502293           10.6807058716

 O196            2.4789202822            -1.8944483777            1.8974446264

 H197            2.8595562063            -2.6728651913            1.3915031403

 O198            3.0214907170            -1.8227624948            7.8576765605

 H199            1.9656620799            -1.2740246938            1.3280595683

 H200            0.8761401230            -2.5711102114            4.2367390301

 H201            3.1368139708            -1.8083404659            6.8681527798

 H202            9.0214061167            -6.3022116867            7.7515897932

 O203           -0.6915958763             1.2568908181            9.3229422088

 H204            0.2005117812             0.8685040108            9.1970199108

 H205           -0.6172063888             2.1461265305            8.9223807617

 O206           -1.5069959798             1.5673670323            5.3614709113

 H207           -2.0477749469             1.2341008644            6.1265670494

 H208           -1.6118659621             2.5332665173            5.3943535655

 O209           -2.7719850981             0.7936720340            7.6179091393

 H210           -3.2599057281            -0.0367823924            7.7117221298

 H211           -2.0481713020             0.7702271953            8.2938613080

 O212            9.0695894103            -3.9863428509            1.7402183950

 H213            8.6269946630            -3.1130519249            1.8186250551

 H214            9.0894249812            -4.1397989512            0.7756396541

 O215            1.2571069629            -5.4768151070            1.6646224300

 H216            2.2311105496            -5.4036868323            1.4853265732

 H217            1.0351936987            -4.8637709533            2.4003597694

 O218            0.3054539767            -4.2100005167            3.9744707803

 H219            0.5654017737            -4.8782405306            4.6534541607

 H220           -0.6646019281            -4.2455333584            3.9629483054

 O221            5.3121456765            -1.2721791353            0.7753950939

 H222            4.8486952338            -2.1225670347            0.5305795030

 H223            4.9694724656            -1.1117033202            1.6825968220

 O224            7.7242153555            -1.4974412252            2.0104361247

 H225            6.9187698979            -1.5545767370            1.4330739603

 H226            7.3990459206            -1.7122326352            2.9107983460

 O227            8.8797253887            -7.9469038809            7.3238344266

 H228            7.9195684826            -8.0045232834            7.5164773778

 H229            8.9037608244            -8.0506983326            6.3544010174

 O230            2.5740072859            -9.7654980370            3.1460609043

 H231            2.4965087192            -9.8549996633            4.1203594233

 H232            3.5050281816           -10.0038757781            2.9044891824

 O233            5.7046811471            -7.8780656128            1.3964791521

 H234            5.0078636539            -7.1841293532            1.3470317642

 H235            6.5274193192            -7.3964680880            1.6502693724

 O236            7.8996087782            -6.4384754826            2.2598913723

 H237            8.0912253994            -6.7431602458            3.1611400081

 H238            8.2807461230            -5.5321618221            2.1620672639

 O239            2.0762052929            -9.6503664643            5.9293691787

 H240            1.5084160767            -8.8444964702            5.9260140094

 H241            1.4861838333           -10.4163709909            6.0823586287

 O242            5.2185668437           -10.1582472931            2.6504454372

 H243            5.4932219722           -10.9037052203            2.0956026872

 H244            5.4253153743            -9.3343780481            2.1181383701

 O245            5.2208740846             0.5019188808           13.0432435404

 H246            5.0675414984             0.8629240593           12.1405785618

 H247            5.6075626281             1.2576841305           13.5116435385

 C248           -1.6084959755#            8.5839729642#           4.3490290369#

 C249           -2.6144314015             7.4191804891            4.4198493594

 C250           -2.1613996521             6.3792483475            5.4192250208

 C251           -2.6374766482             6.3700358833            6.7357318740

 C252           -1.1474954095             5.4719326470            5.0797225859

 C253           -2.0915689464             5.5270190269            7.7048941864

 C254           -0.5829159737             4.6280446106            6.0359917654

 C255           -1.0329093961             4.6788617826            7.3603200836

 O256           -0.3792009783             3.9205196626            8.3006957671

 H257           -1.5068803325             9.0701625776            5.3259110720

 H258           -2.7205204446             6.9676238119            3.4251552535

 H259           -3.6034401749             7.8046530854            4.6973838783

 H260           -3.4396465140             7.0479826175            7.0193472394

 H261           -0.7812004480             5.4301939353            4.0593229300

 H262           -2.4606788213             5.5480376419            8.7242329105

 H263            0.2543249874             3.9819754103            5.7891949260

 H264           -1.9277176454             9.3416302255            3.6232765155

 H265           -0.6176716257             8.2237116585            4.0504195797

 C266           -1.2156619977#            3.5767179903#          14.9580159916#

 C267           -2.1636946307             4.4855944504           14.1583669901

 C268           -1.5941492686             4.8144065882           12.8162946839

 N269           -0.7383829989             5.8827152902           12.6161509111

 C270           -1.6322276131             4.1548031824           11.6130511667

 C271           -0.3016264383             5.8358616234           11.3386201947

 N272           -0.8221009602             4.7993069444           10.7032279631

 H273           -0.2249933965             4.0323160329           15.0651946493

 H274           -2.3655689868             5.4124555029           14.7101250421

 H275           -3.1302233913             3.9861720707           14.0280872849

 H276           -0.4446427323             6.5541785282           13.3318157119

 H277           -2.1636897698             3.2521710750           11.3472612643

 H278            0.3748061242             6.5628590511           10.9133620358

 H279           -0.5671626863             4.2906849203            9.2510895715

 H280           -1.6119813181             3.3798602329           15.9617917879

 H281           -1.0876772114             2.6185528050           14.4522961110

 C282           -0.2371729981#            6.7063619679#          17.0391969374#

 C283            0.6397716351             7.0068353556           15.8400383370

 O284            0.1610149808             7.4412607147           14.7889824760

 N285            1.9711929588             6.7911849768           15.9850585364

 H286           -1.0644050272             6.0690903359           16.7165060753

 H287            0.2797840005#            6.2148220178#          17.8670170102#

 H288            2.3794234153             6.4771057355           16.8524542189

 H289            2.5866130016             7.0212624229           15.2162780189

 H290           -0.6698191673             7.6457060743           17.3988282079

 C291           -1.7760929908#           -3.1799650182#           9.5045650286#

 C292           -1.9928331190            -3.3428913078            7.9932356167

 C293           -1.0486905381            -2.4234573347            7.2059870488

 C294           -1.8400985675            -4.8082940483            7.5728768791

 H295           -3.0287876432            -3.0407056422            7.7684962287

 H296           -1.2639508763            -1.3661672517            7.3918387350

 H297           -1.1359435086            -2.5913715287            6.1253558787

 H298           -0.0018314505            -2.6006062789            7.4826611044

 H299           -2.0023325540            -4.9354553390            6.4955205409

 H300           -2.5567220140            -5.4511218137            8.0990118988

 H301           -0.8322771793            -5.1826961137            7.7840091798

 H302           -2.0586670100#           -2.1742879923#           9.8440009690#

 H303           -0.7230583599            -3.3457359710            9.7666647231

 H304           -2.3800413451            -3.8978808071           10.0735644879

304 atom structure with an oxyl radical

 Mn1             2.8322671675            -1.4549794037            3.7841321702

 Mn2             4.5205686984            -1.4596305209            8.6387044728

 Mn3             5.1731596395            -1.4155131217            5.3319450372

 Mn4             6.4054401589             0.1412299437            7.3127485140

 O5              3.4741617129            -1.2100074226            5.7266513157

 O6              5.5368605536             0.3444315514            5.7495485953

 O7              4.9351814914             0.3319889641            8.3402004814

 O8              5.8137895000            -1.6691130129            7.2024106102

 Ca9             3.1899133483             1.1228841111            6.8584804468

 C10             2.1876270637#            1.2855489255#          -1.5249169858#

 C11             1.3621649775             0.8687973752           -0.3178468097

 O12             1.0766838893            -0.3126023133           -0.1023790945

 C13             3.5348431969             0.5487698669           -1.5106159319

 O14             4.2714530682             0.9214911092           -0.3599729542

 H15             1.6292916410             1.0154823889           -2.4297154036

 H16             3.3409223644            -0.5312839512           -1.5273017649

 H17             4.0878036761             0.8056433663           -2.4278306112

 H18             4.6209796699             0.1009252648            0.0562466816

 H19             2.3565269893             2.3686287197           -1.5431932868

 N20             0.9385051118             1.8723615874            0.5063474874

 C21             0.2490310819             1.5985792625            1.7563787252

 C22            -1.2495435537             1.8706868343            1.6537290680

 O23            -2.0206987180             1.7039411641            2.5795161703

 C24             0.8859152885             2.3035876997            2.9672884654

 C25             1.9809840114             1.5023187379            3.6891870653

 O26             2.5296900257             2.0356507834            4.6728145720

 O27             2.2061461289             0.3328526714            3.2226660498

 H28             1.2965561762             2.8037752074            0.3433384096

 H29             0.3399459753             0.5166150112            1.9073983793

 H30             0.1163103230             2.5058950537            3.7189221067

 H31             1.2986239841             3.2807895067            2.6883686228

 H32            -1.6212511951             2.2048943579            0.6642903122

 C33             1.0582597324#            1.1829963128#          13.4563129369#

 H34             0.0279795967             0.8063101866           13.4626225914

 H35             1.0543080902             2.1938667092           13.8733156746

 H36             1.6503564542             0.5489849230           14.1279488965

 C37             1.6457704382             1.1744531700           12.0501714574

 H38             2.6544100556             1.6030874745           12.0653889681

 H39             1.0421951691             1.7904575025           11.3748829108

 C40             1.7209248704            -0.2555423674           11.4985180013

 H41             0.7063576716            -0.6532949815           11.3561183906

 H42             2.2530086225            -0.9057907427           12.1973949843

 C43             2.4295131751            -0.2845905343           10.1614193655

 O44             2.0253727799             0.3962810505            9.2036948094

 O45             3.4851022285            -1.0532499863           10.1555432776

 C46             3.9881490622            -8.1200756337            8.4290948812

 H47             3.3836518091            -9.0050275288            8.2046742972

 H48             4.6062960937            -8.3428183129            9.3047566741

 C49             4.9397355725            -7.8297199178            7.2769261401

 O50             6.0746075501            -7.3546409895            7.4877859665

 C51             3.0116082706            -6.9474507397            8.7265326756

 H52             2.3784252049            -7.2530678013            9.5708066934

 H53             2.3381454334            -6.8118193413            7.8757041202

 C54             3.6454860148            -5.6247424416            9.0276200884

 N55             4.4018319334            -5.3730690102           10.1686724354

 H56             4.6127759932            -6.0375353760           10.9003283790

 C57             3.6190121230            -4.4273459417            8.3587621682

 H58             3.1114026364            -4.1619141112            7.4458002778

 C59             4.8005129203            -4.0815794196           10.1558472867

 H60             5.3840058715            -3.5985389048           10.9234424732

 N61             4.3446234951            -3.4869358182            9.0652625910

 N62             4.4692142960            -8.0823391723            6.0417645127

 H63             3.5855140049            -8.5904520571            5.9657068644

 C64             5.2069915040#           -7.7515172479#           4.8093069834#

 C65             6.5262567689            -8.5246645510            4.8753977440

 O66             7.6326762063            -8.0611296861            4.6626501925

 H67             6.4114970950            -9.5792740419            5.1856860046

 H68             4.6233333258            -8.1858965422            3.9931953964

 C69             5.3977215129            -6.2412200621            4.5906341535

 H70             5.9956070001            -6.1092996542            3.6845931252

 H71             5.9778118986            -5.8291559214            5.4176469240

 C72             4.0607207319            -5.5138935437            4.4537650073

 H73             3.5722223799            -5.7738566541            3.5114580191

 H74             3.3648241640            -5.8154902173            5.2473998535

 C75             4.1083133280            -3.9998625727            4.5070659335

 O76             3.1338699864            -3.4155046378            3.9381046852

 O77             5.0546526156            -3.4118650111            5.1238472275

 C78             9.1595114464#            0.1191579452#          11.4478227394#

 H79             9.8482427893            -0.0903860310           10.6165176431

 H80             9.7193991953            -0.1388852295           12.3597945233

 C81             7.8823791954            -0.7288919958           11.3617314648

 H82             7.1373106182            -0.4005451765           12.1002538285

 H83             8.1183355019            -1.7657896892           11.6297631943

 C84             7.1319356641            -0.7957927024           10.0264590641

 O85             7.5487644609            -0.1271428332            9.0244938818

 O86             6.1150444919            -1.5489603351           10.0188828213

 C87             8.9598597596             1.6139777563           11.4841325846

 O88             7.8928503901             2.1950357458           11.4981004476

 H89             9.9001824848             2.2035737467           11.5243584672

 C90             7.7935564059#            4.3107436377#           8.7015790000#

 H91             8.1688537159             5.3208216119            8.9000278283

 H92             7.3852885063             3.9108186501            9.6332051918

 H93             8.6366330118             3.6789853424            8.4092765647

 C94             6.7346513317             4.3495422735            7.5978168192

 H95             5.9026052577             5.0092403843            7.8659284324

 H96             7.1648301194             4.7598933618            6.6717344952

 C97             6.1280565683             2.9979463878            7.2314551772

 O98             4.9823696794             2.9501588983            6.7368797249

 O99             6.9015025475             1.9793140725            7.4372282828

 C100           11.5816617653#           -0.6170833992#           3.4808992007#

 H101           12.2647215362            -0.9959506462            4.2506147071

 H102           12.0613861088             0.2458020652            3.0061659070

 H103           11.4610508655            -1.3978194118            2.7217431006

 C104           10.2250541906            -0.2293196646            4.0841936759

 H105            9.5715653635             0.1549754556            3.2915211134

 H106           10.3466463645             0.5738731848            4.8211981980

 C107            9.5391329029            -1.4340376288            4.7582384984

 H108           10.1601121165            -1.7787465766            5.5940102950

 H109            9.4489197715            -2.2585245981            4.0458933209

 C110            8.1623537939            -1.1143922072            5.3210679399

 O111            7.1503165618            -1.6273539264            4.7368944506

 O112            8.1143774157            -0.3695590177            6.3504832844

 C113           10.8187240932#            4.2285681063#          -0.8150212789#

 H114           11.5788847958             3.6066087364           -0.3285767051

 H115           11.1145511061             5.2772587363           -0.6957540196

 H116           10.8292319412             3.9958650227           -1.8844895599

 C117            9.4247811905             3.9770345211           -0.2132643474

 H118            9.1432386542             2.9309207853           -0.4048113897

 H119            8.6834548139             4.5944555909           -0.7398186433

 C120            9.3524369735             4.2803759842            1.2942872353

 H121            9.7013764585             5.3073639678            1.4655837174

 H122           10.0355834103             3.6222055501            1.8469135563

 C123            7.9499602064             4.1791167525            1.9190561997

 H124            7.2546277415             4.8421155174            1.3831956437

 H125            7.9947095808             4.5127323121            2.9607646589

 N126            7.4410733536             2.8052629155            1.9192046108

 H127            7.5918057428             2.2562836707            1.0826820014

 C128            6.6325196903             2.2393141537            2.8411196999

 N129            6.0479080577             2.9732408748            3.8025322449

 H130            5.5923561337             2.5177884929            4.5928602264

 H131            5.8938798694             3.9627892278            3.6860411056

 N132            6.4297333538             0.9290338218            2.8019203445

 H133            7.0572964245             0.2803976081            2.3160410327

 H134            5.6918953186             0.4886983501            3.3568540330

 C135           11.8423975510#           -5.3930895523#           7.2492549556#

 H136           11.3688498438            -6.3785301740            7.1858451106

 H137           12.9094874818            -5.5530178460            7.4335069832

 H138           11.7340586257            -4.8985363339            6.2783884201

 C139           11.2276283251            -4.5459072796            8.3737422732

 H140           11.7534676051            -3.5886654935            8.4592445108

 H141           11.3594801418            -5.0590954436            9.3360118793

 C142            9.7768582898            -4.2581251735            8.1488008831

 N143            8.8396175551            -5.2607597838            7.9367038181

 C144            9.0837608338            -3.0782184484            8.0760822484

 H145            9.4055884045            -2.0533163347            8.1702542953

 C146            7.6381743929            -4.7083452336            7.7396740735

 H147            6.7327563976            -5.2639360137            7.5468604199

 N148            7.7621397245            -3.3878531199            7.8220014941

 H149            6.9840068778            -2.7072630200            7.6249992129

 C150            6.0989354935#           -4.2351882776#          -1.2221974308#

 H151            5.5366578444            -4.7807004538           -1.9944974899

 C152            7.5233420626#           -4.0871570677#          -1.7388094256#

 H153            7.5924900737            -3.8166251647           -2.8143001308

 O154            8.5471230708            -4.2139994716           -1.0950519015

 H155            5.6679400817            -3.2230765408           -1.2362326828

 C156            5.9272091062            -4.8853950931            0.1424168479

 H157            6.2395605815            -5.9289578904            0.1051678352

 H158            6.5703626649            -4.3907934050            0.8789085690

 C159            4.4736763697            -4.7953115309            0.6211098640

 O160            3.8840037176            -3.6785871230            0.4700365835

 O161            3.9421194255            -5.8210919187            1.1349009547

 C162           -1.8288744813#          -11.9867165743#           4.2485682426#

 H163           -1.3602391462           -12.9321182159            3.9577037557

 H164           -2.8396260208           -12.1879855231            4.6266058428

 C165           -1.0204727248           -11.3419893987            5.3442093110

 H166           -1.5235807384           -10.5000106912            5.8677947531

 O167            0.1279430359           -11.6339201168            5.6281946427

 C168           -1.9078520538           -11.0102958103            3.0465250530

 H169           -2.6883576092           -11.3620004416            2.3608356611

 H170           -2.2476031473           -10.0306851321            3.4128677358

 C171           -0.5699400380           -10.8862277821            2.3017173762

 H172            0.2592693127           -10.7442139171            3.0028282385

 H173           -0.3613442292           -11.8415086058            1.8029620978

 C174           -0.5318256686            -9.7727249197            1.2424093652

 H175            0.3776647209            -9.8832590584            0.6391051203

 H176           -1.3749853224            -9.8898943355            0.5478553549

 C177           -0.5895760047            -8.3396533658            1.7791915945

 H178           -0.6895510656            -7.6343339820            0.9499658491

 H179           -1.4515912189            -8.1927940912            2.4362900172

 N180            0.6403450193            -7.9219102775            2.5379885696

 H181            1.0027550743            -7.0121610330            2.0964279780

 H182            0.4538341810            -7.6869548130            3.5268726855

 H183            1.4086371664            -8.6429530754            2.5594213691

 Cl184           0.7687190533            -6.6940026195            5.5915845216

 O185            0.9380378416            -1.8830660902            4.3874977430

 O186            4.6243844808            -1.1404709356            3.5956959150

 O187            0.9399752002             0.2766580990            6.5991460898

 H188            0.3103534114             0.3207041107            5.8478198685

 H189            1.0245808956            -0.6654593765            6.8254631697

 O190            2.4261023516             3.1706578594            7.8744504221

 H191            1.4884470291             3.4274454824            8.0219650447

 H192            2.9565394901             3.9715705373            7.7657621209

 O193            5.1236175962             1.7173582536           10.8252847927

 H194            5.0308814350             1.2645219969            9.9606802279

 H195            6.0761718370             1.8982788767           10.9231888490

 O196            2.2982275094            -2.0029353662            1.7617736907

 H197            2.6925579455            -2.7922180793            1.3093726355

 O198            3.1485474895            -1.5655234365            7.6741188671

 H199            1.8764249861            -1.4218063054            1.0951502333

 H200            0.6332315934            -2.7875983006            4.0855801107

 H201            0.1892366057            -1.2382753862            4.3180685732

 H202            8.9956587405            -6.2893908515            7.8101026134

 O203           -0.8341556226             1.0462401541            9.0202643480

 H204            0.0878552503             0.7090066761            9.0161165018

 H205           -0.7307129847             1.9427956935            8.6413567685

 O206           -1.0027547680             0.0114138573            4.6687312079

 H207           -1.5929187274             0.0057158614            5.4762042481

 H208           -1.4091754275             0.6417860450            4.0375919092

 O209           -2.4427677415             0.3683562845            6.9057221522

 H210           -3.0316588944            -0.3281556382            7.2317401701

 H211           -1.8438640163             0.5730039433            7.6775307506

 O212            8.9934307411            -4.0444992323            1.7394269228

 H213            8.5549788595            -3.1690869893            1.8182760490

 H214            9.0333620606            -4.1898072454            0.7740853235

 O215            1.2383516462            -5.6083864105            1.3837543034

 H216            2.2250382543            -5.5602224368            1.2672681081

 H217            0.9931587873            -4.9662737867            2.0833144277

 O218            0.1700742757            -4.3218446914            3.6650976611

 H219            0.4559639711            -4.9741876457            4.3548393087

 H220           -0.7980570530            -4.4009173588            3.6536016727

 O221            5.2108851750            -1.3971874459            0.8713274497

 H222            4.7528433016            -2.2435675598            0.6002264209

 H223            4.8832546877            -1.2609085200            1.7907638323

 O224            7.6676719029            -1.5416533022            1.9779216239

 H225            6.8438415206            -1.6310449519            1.4300281245

 H226            7.3730048563            -1.7325032602            2.8957228609

 O227            8.7973949498            -7.9350864812            7.3971469302

 H228            7.8305588007            -7.9634808471            7.5604546270

 H229            8.8507121377            -8.0613743590            6.4317538049

 O230            2.5030852727            -9.8532230400            3.0774603431

 H231            2.4229172409            -9.8634860997            4.0559496005

 H232            3.4542584870           -10.0446738524            2.8727570627

 O233            5.7354582313            -7.9740530224            1.3852761528

 H234            5.0334919020            -7.2872588866            1.3011148239

 H235            6.5520389089            -7.4814651510            1.6410252433

 O236            7.9439575298            -6.5534217919            2.2464074649

 H237            8.0960101276            -6.8296802151            3.1646262074

 H238            8.2692952318            -5.6248331270            2.1601027642

 O239            2.0096194305            -9.5485000449            5.8421244929

 H240            1.4363899840            -8.7496283158            5.8307992437

 H241            1.4366220202           -10.3117268540            6.0611763916

 O242            5.1816939945           -10.1847029257            2.7152456038

 H243            5.4736271845           -10.9525793351            2.2011032674

 H244            5.4189660180            -9.3824902643            2.1591887652

 O245            5.0531201436             0.0473051543           13.0588049496

 H246            4.9549563704             0.5054675505           12.1919683338

 H247            5.1677228265             0.7824145298           13.6801099002

 C248           -1.6084959358#            8.5839729280#           4.3490290520#

 C249           -2.2366366834             7.2000316729            4.0999245992

 C250           -1.7982789149             6.1896054709            5.1376697844

 C251           -2.3374696263             6.2243554623            6.4317884076

 C252           -0.7862553944             5.2566200871            4.8779950470

 C253           -1.8699198285             5.3860338615            7.4408949283

 C254           -0.2967848754             4.4108968180            5.8756775063

 C255           -0.8312476650             4.4880575419            7.1648395977

 O256           -0.2857635886             3.7026936264            8.1521487210

 H257           -1.8871261566             8.9667808186            5.3369357979

 H258           -1.9625286021             6.8478537038            3.0972637522

 H259           -3.3304804958             7.2948479791            4.1058520683

 H260           -3.1346495171             6.9287693612            6.6596582945

 H261           -0.3519800646             5.2076536663            3.8811964940

 H262           -2.2927685237             5.4337204792            8.4386791707

 H263            0.5198493575             3.7231280254            5.6768613346

 H264           -1.9399010482             9.3109688499            3.5982043081

 H265           -0.5146107323             8.5284676232            4.3107980259

 C266           -1.2156620077#            3.5767179788#          14.9580159549#

 C267           -2.2178252605             4.2410410290           13.9998909778

 C268           -1.6017030640             4.5538643394           12.6744199471

 N269           -0.7481337460             5.6305827608           12.5025692348

 C270           -1.6210807052             3.9156513739           11.4588455915

 C271           -0.2975339620             5.6081233605           11.2286968237

 N272           -0.8075968078             4.5834598863           10.5662727577

 H273           -0.3100626010             4.1825829419           15.0643742646

 H274           -2.6127332749             5.1624808183           14.4480860368

 H275           -3.0771088420             3.5793920775           13.8454538165

 H276           -0.4564292317             6.2821064309           13.2353548493

 H277           -2.1466104883             3.0166258721           11.1698012625

 H278            0.3821238091             6.3450581493           10.8260262596

 H279           -0.5140334739             4.0802050330            9.0853734609

 H280           -1.6572327758             3.4459054290           15.9534869917

 H281           -0.9231361881             2.5940131990           14.5885606409

 C282           -0.2371729546#            6.7063619429#          17.0391968780#

 C283            0.6544546527             7.0013119743           15.8456171685

 O284            0.1933084335             7.1619792580           14.7139616324

 N285            1.9828972516             7.1201212271           16.0978128269

 H286           -1.0656805397             6.0766685026           16.7108234516

 H287            0.2797839751#            6.2148220264#          17.8670170312#

 H288            2.3701223312             7.0264698221           17.0246191720

 H289            2.6013539750             7.3684673819           15.3374680892

 H290           -0.6601806484             7.6489171998           17.4054396393

 C291           -1.7760929949#           -3.1799649729#           9.5045649024#

 C292           -2.3444413583            -3.5752957700            8.1298320354

 C293           -1.5194903212            -2.9655329880            6.9886099334

 C294           -2.4169839973            -5.1019047895            7.9862651118

 H295           -3.3721672515            -3.1862423852            8.0598600798

 H296           -1.5031059530            -1.8730764518            7.0425797258

 H297           -1.9312767881            -3.2444173243            6.0095868047

 H298           -0.4844757999            -3.3269562833            7.0232378953

 H299           -2.8334965271            -5.3896577407            7.0133924513

 H300           -3.0472831081            -5.5437787295            8.7672359315

 H301           -1.4192522075            -5.5521284775            8.0555383482

 H302           -2.0586670001#           -2.1742880128#           9.8440010382#

 H303           -0.6799400110            -3.2431221088            9.5070766334

 H304           -2.1392114302            -3.8681916043           10.2788563309

309 atom structure with an OH

 Mn1             2.9444801386            -1.4252511260            3.8167682175

 Mn2             4.5747591380            -1.5777756792            8.7582156146

 Mn3             5.2995291856            -1.4458804146            5.2547472586

 Mn4             6.4699488634            -0.0130005650            7.3595634634

 O5              3.5206061396            -1.1266815594            5.5146559499

 O6              5.6911945890             0.2492748222            5.7678015663

 O7              4.9480843171             0.1882701861            8.3465875537

 O8              5.8025379890            -1.7984080137            7.2576111904

 Ca9             3.2439815816             1.1282438362            6.8717024054

 C10             2.1876270803#            1.2855489119#          -1.5249168648#

 C11             1.5554835389             0.9620983606           -0.1770107919

 O12             1.2753828858            -0.1970066210            0.1463737216

 C13             3.5203769597             0.5313480032           -1.6558500679

 O14             4.4156046815             0.9543826463           -0.6422549300

 H15             1.4977253216             0.9601079717           -2.3129908137

 H16             3.3185803075            -0.5451610513           -1.5863890748

 H17             3.9457588830             0.7303855781           -2.6514608147

 H18             4.7625145881             0.1571075636           -0.1845530140

 H19             2.3562309735             2.3619001137           -1.6443484755

 N20             1.3198294836             2.0285151092            0.6339033934

 C21             0.6646333795             1.9503168833            1.9370664141

 C22            -0.6744752396             2.6795769952            1.7812539676

 O23            -1.0247488868             3.6441078844            2.4221252881

 C24             1.5078410780             2.4975426326            3.0869753934

 C25             2.5574710339             1.5525308826            3.6730034647

 O26             3.3124246448             1.9904205341            4.5655441244

 O27             2.5506937702             0.3581358131            3.2006758200

 H28             1.6121640668             2.9410136354            0.3096114294

 H29             0.4508719409             0.8889736050            2.1019657433

 H30             0.8336881477             2.7316634225            3.9176375794

 H31             1.9959082739             3.4427307663            2.8224797664

 H32            -1.3102483145             2.2682927311            0.9684733939

 C33             1.0582598319#            1.1829964034#          13.4563130599#

 H34            -0.0118773750             1.0070907225           13.2940344607

 H35             1.1700080571             2.1553673119           13.9459095685

 H36             1.4239482122             0.4168767344           14.1510645371

 C37             1.8269681532             1.1421502375           12.1399360791

 H38             2.8832754414             1.3561493085           12.3307543580

 H39             1.4430904971             1.9057199991           11.4543344291

 C40             1.7023240889            -0.2336835041           11.4632542319

 H41             0.6515556094            -0.4193670132           11.2064455731

 H42             2.0405155599            -1.0258900584           12.1368135192

 C43             2.5134601772            -0.2828517272           10.1846046681

 O44             2.2255362882             0.4429812223            9.2182046921

 O45             3.5328639266            -1.0993223279           10.2428934248

 C46             4.0768333724            -8.2616209092            8.4452250618

 H47             3.4479242256            -9.1235728634            8.2000402234

 H48             4.7277656533            -8.5402550134            9.2802785307

 C49             4.9880020988            -7.9268819982            7.2725413282

 O50             6.1357099567            -7.4748120705            7.4641952859

 C51             3.1327982348            -7.0933366714            8.8484787203

 H52             2.5491592002            -7.4300977266            9.7163316381

 H53             2.4115937908            -6.9205860095            8.0451645977

 C54             3.7850041965            -5.7820073754            9.1579604762

 N55             4.6311672622            -5.5601577686           10.2405072972

 H56             4.9170273553            -6.2478897876           10.9236681968

 C57             3.6802009241            -4.5603203960            8.5460139195

 H58             3.0792746030            -4.2754596768            7.6993623189

 C59             5.0044443752            -4.2598606974           10.2447756850

 H60             5.6482205314            -3.8003526248           10.9773590610

 N61             4.4479024114            -3.6305412514            9.2228273402

 N62             4.4792086985            -8.1101088389            6.0395571696

 H63             3.5920032024            -8.6086731932            5.9595885209

 C64             5.2069916540#           -7.7515171149#           4.8093070017#

 C65             6.5389398659            -8.5031457315            4.8656940258

 O66             7.6396748258            -8.0274891411            4.6519572530

 H67             6.4364738422            -9.5628659470            5.1638841845

 H68             4.6262493763            -8.1877171486            3.9921840735

 C69             5.3748871078            -6.2355656329            4.5976841133

 H70             5.9665514999            -6.0934793753            3.6892395708

 H71             5.9592203428            -5.8229326305            5.4215253838

 C72             4.0385092290            -5.4973772796            4.4763087542

 H73             3.5253592968            -5.7519341448            3.5466289682

 H74             3.3589238889            -5.7777181687            5.2911993133

 C75             4.1374971506            -3.9838212657            4.5239501434

 O76             3.1836906728            -3.3531797212            3.9550838871

 O77             5.0970759259            -3.4347185466            5.1410026702

 C78             9.1595115826#            0.1191579841#          11.4478227917#

 H79             9.8476540999            -0.1429046264           10.6320633167

 H80             9.7247738674            -0.0552242251           12.3760962701

 C81             7.8880563387            -0.7417747107           11.4356720929

 H82             7.1383437808            -0.3349926934           12.1300768813

 H83             8.1159287796            -1.7549643757           11.7872556425

 C84             7.1558850964            -0.8839407981           10.1021691880

 O85             7.5868074936            -0.2720479664            9.0786695097

 O86             6.1266156210            -1.6291763972           10.1128917663

 C87             8.9165221795             1.6086890512           11.3680342895

 O88             7.8294323315             2.1509617468           11.3697292193

 H89             9.8387847940             2.2271982166           11.3285441420

 C90             7.7935565057#            4.3107436824#           8.7015790579#

 H91             8.3100798509             5.2750378344            8.7539536618

 H92             7.0645390673             4.2752853079            9.5170964342

 H93             8.5223780500             3.5141036643            8.8651498009

 C94             7.1055560208             4.1475465666            7.3382440788

 H95             6.3930059054             4.9567281463            7.1542462954

 H96             7.8612967031             4.1803115229            6.5407653526

 C97             6.3478411074             2.8326808231            7.1888630973

 O98             5.1759360489             2.8111322322            6.7943010658

 O99             7.0551544008             1.7806694633            7.5076987601

 C100           11.5816617623#           -0.6170833907#           3.4808991682#

 H101           12.3098009628            -1.0175178332            4.1964873637

 H102           12.0207710522             0.2782061439            3.0270944968

 H103           11.4392459505            -1.3626266989            2.6907908131

 C104           10.2509610742            -0.2896547534            4.1659696393

 H105            9.5501277928             0.1126379532            3.4247020784

 H106           10.3885369483             0.4829409824            4.9320187474

 C107            9.6267950838            -1.5402524240            4.8168251468

 H108           10.2741732510            -1.8870915671            5.6312776228

 H109            9.5454919634            -2.3451323800            4.0813781514

 C110            8.2540054342            -1.2661099228            5.4018371906

 O111            7.2474109323            -1.7615510787            4.7929051832

 O112            8.1941664635            -0.5659244553            6.4589269183

 C113           10.8187241122#            4.2285681205#          -0.8150213164#

 H114           11.5546142713             3.5298147618           -0.4003563367

 H115           11.1951093994             5.2459327734           -0.6563697861

 H116           10.7645084583             4.0559565286           -1.8946109756

 C117            9.4425596703             4.0405266011           -0.1593376190

 H118            9.0855353921             3.0238107561           -0.3816054351

 H119            8.7163495548             4.7230213151           -0.6218931903

 C120            9.4640820668             4.2856159329            1.3593836806

 H121            9.8330224612             5.3013802946            1.5506510226

 H122           10.1731588136             3.6007856816            1.8435745164

 C123            8.1007414061             4.1581598205            2.0555085236

 H124            7.3765011322             4.8386661388            1.5822435682

 H125            8.1960147903             4.4484658310            3.1077517169

 N126            7.6155873702             2.7780911660            2.0132716847

 H127            7.8884546935             2.2134526115            1.2201014584

 C128            6.6992623011             2.2257560944            2.8369937104

 N129            6.0437793491             2.9801408840            3.7330760400

 H130            5.3530210859             2.5479594713            4.3494653261

 H131            5.9281007733             3.9690411842            3.5667513874

 N132            6.4902310082             0.9176020828            2.7744891094

 H133            7.1525930854             0.2755847813            2.3311313843

 H134            5.7403357267             0.4526293904            3.2948645684

 C135           11.8423975822#           -5.3930895404#           7.2492549458#

 H136           11.3817577970            -6.3749664604            7.0977462976

 H137           12.9106606356            -5.5555934023            7.4251855926

 H138           11.7328647035            -4.8177610598            6.3241192786

 C139           11.2089284805            -4.6545141788            8.4374339151

 H140           11.7191126912            -3.7006358591            8.6099822608

 H141           11.3375167201            -5.2465850160            9.3534539448

 C142            9.7585829695            -4.3735593381            8.2103317078

 N143            8.8389909463            -5.3807668193            7.9495160515

 C144            9.0557366372            -3.1995546383            8.1487734440

 H145            9.3655329381            -2.1741825419            8.2716811168

 C146            7.6381337102            -4.8350221797            7.7327322992

 H147            6.7444453760            -5.3962517863            7.5078119626

 N148            7.7444907553            -3.5161105706            7.8508099892

 H149            6.9600649094            -2.8376555243            7.6497064965

 C150            6.0989356866#           -4.2351883260#          -1.2221974870#

 H151            5.5488230006            -4.8237451731           -1.9716018073

 C152            7.5233419973#           -4.0871570086#          -1.7388095556#

 H153            7.5926009017            -3.8165422458           -2.8142251088

 O154            8.5465083417            -4.2161142670           -1.0948597645

 H155            5.6519682845            -3.2319822624           -1.2813867656

 C156            5.9443574837            -4.8300790970            0.1680562944

 H157            6.2467648035            -5.8765640434            0.1650688788

 H158            6.6048370998            -4.3123218430            0.8725199472

 C159            4.5043217734            -4.7048950896            0.6731880141

 O160            3.9414470491            -3.5713117677            0.5178736226

 O161            3.9600081103            -5.7098982261            1.2076042782

 C162           -1.8288747219#          -11.9867168118#           4.2485682107#

 H163           -1.3703911642           -12.9137692197            3.8906425744

 H164           -2.8380293892           -12.2038056512            4.6213623291

 C165           -1.0031419138           -11.4177214666            5.3720904609

 H166           -1.5024911663           -10.6190686855            5.9644752470

 O167            0.1534604566           -11.7155082717            5.6110500185

 C168           -1.9062560008           -10.9313089395            3.1115703143

 H169           -2.6923453733           -11.2343847619            2.4091480234

 H170           -2.2372288306            -9.9741276195            3.5409025895

 C171           -0.5726254485           -10.7637117985            2.3661732461

 H172            0.2582191394           -10.6375492043            3.0687127872

 H173           -0.3565431906           -11.6994513190            1.8343714359

 C174           -0.5512051035            -9.6155631096            1.3424075271

 H175            0.3487714887            -9.7100520255            0.7222214460

 H176           -1.4037631249            -9.7155091745            0.6565607523

 C177           -0.6002152331            -8.1957742847            1.9155378020

 H178           -0.7211481005            -7.4726732796            1.1045550160

 H179           -1.4433768532            -8.0577183596            2.5978408500

 N180            0.6473269258            -7.7915120488            2.6519800243

 H181            0.9991480400            -6.8595029405            2.2358567142

 H182            0.4857460821            -7.5927271287            3.6514010027

 H183            1.4166950822            -8.5057194537            2.6351996683

 Cl184           0.8058100033            -6.7509129783            5.7911734342

 O185            1.2433714887            -1.5736298525            4.3446043833

 O186            4.8714360088            -1.2141997669            3.5514822883

 O187            1.0373057677             0.5889331295            6.0652873760

 H188            0.2180562484             1.0869826698            5.8052667732

 H189            0.9645371535            -0.2697102333            5.5869024741

 O190            2.6949712887             3.1412740283            8.1340399886

 H191            3.1871750249             3.1590784730            8.9725377676

 H192            1.7803177567             3.4546207189            8.3147141095

 O193            5.0208966660             2.0817784241           10.3630603516

 H194            4.9927018338             1.3652371889            9.6869955002

 H195            5.9696856598             2.2816515973           10.4541234299

 O196            2.5374503328            -1.8788268360            1.9117821356

 H197            2.8872968924            -2.6796179901            1.4229243771

 O198            3.0714093994            -1.8395423078            7.8738845933

 H199            2.0279299827            -1.2654562694            1.3299854311

 H200            0.9150429435            -2.5071321101            4.2077968021

 H201            3.1782859461            -1.7941603328            6.8834951527

 H202            9.0125310593            -6.4038475062            7.8018081726

 O203           -0.5113049344             1.3055582526            9.2620489866

 H204            0.3726322608             0.8966528366            9.1535047639

 H205           -0.4019840253             2.1869805584            8.8542652948

 O206           -1.3311002339             1.6713079964            5.2972059164

 H207           -1.8726111336             1.3086840759            6.0532361616

 H208           -1.4256900638             2.6359205311            5.3638182561

 O209           -2.5789636065             0.8062819261            7.5187269821

 H210           -3.0698882338            -0.0270394876            7.6113356788

 H211           -1.8657284753             0.7931342660            8.2005488393

 O212            9.0884530719            -4.0591087945            1.7213176116

 H213            8.6382903258            -3.1888634589            1.7988143212

 H214            9.1106670655            -4.2144410247            0.7572039925

 O215            1.2619082663            -5.4416536407            1.6028944834

 H216            2.2365609102            -5.4015496186            1.4302986108

 H217            1.0670653408            -4.8079506859            2.3339548392

 O218            0.4051090365            -4.1050688860            3.8878127210

 H219            0.6877833631            -4.7343305373            4.5796303105

 H220           -0.5818697983            -4.1688863996            3.8665283421

 O221            5.3268111472            -1.3009556528            0.7595310546

 H222            4.8582020492            -2.1524537933            0.5278307514

 H223            4.9855357385            -1.1237477674            1.6633031489

 O224            7.7283145265            -1.5882800730            1.9960180833

 H225            6.9218371944            -1.6250302404            1.4185314925

 H226            7.4003999816            -1.8075826545            2.8940473083

 O227            8.8526273073            -8.0484345957            7.3690863810

 H228            7.8895808883            -8.0983160993            7.5463230392

 H229            8.8900055331            -8.1415317773            6.3988554641

 O230            2.5389816463            -9.7198060084            3.1355776968

 H231            2.4484476098            -9.8216277406            4.1073688370

 H232            3.4651094651            -9.9858459319            2.9025283515

 O233            5.7018998953            -7.9202154127            1.4048356771

 H234            5.0071310513            -7.2246810185            1.3387110573

 H235            6.5219911135            -7.4356046250            1.6623263974

 O236            7.8870869094            -6.4770597308            2.2734364856

 H237            8.0969154083            -6.7744982871            3.1729579800

 H238            8.2868905078            -5.5805512546            2.1531097099

 O239            2.0264918274            -9.6316368924            5.9187938279

 H240            1.4424645769            -8.8414186792            5.9369164164

 H241            1.4577236124           -10.4101195549            6.0895412608

 O242            5.1789564179           -10.1817046022            2.6767124556

 H243            5.4374496231           -10.9366012181            2.1267821340

 H244            5.4025523487            -9.3661278791            2.1369095920

 O245            5.3215152250             0.7765627204           12.8887775727

 H246            5.1064818460             1.1207377524           11.9938890423

 H247            5.7625120475             1.5315167806           13.3078985458

 C248           -1.6084959843#            8.5839729751#           4.3490290187#

 C249           -2.3220663342             7.2191298050            4.3308719134

 C250           -1.7593057441             6.2784361446            5.3735077556

 C251           -2.2672641648             6.2565232180            6.6790273932

 C252           -0.6575933585             5.4601105472            5.0861999905

 C253           -1.6878158749             5.4734501154            7.6769070007

 C254           -0.0599414816             4.6723788329            6.0720413185

 C255           -0.5657223412             4.6907221642            7.3760192928

 O256            0.0697019679             3.9496934121            8.3398181476

 H257           -1.7071283990             9.0632183321            5.3297502751

 H258           -2.2282630736             6.7679842926            3.3350190308

 H259           -3.3953527427             7.3695487287            4.5049529303

 H260           -3.1326450524             6.8679470103            6.9262146121

 H261           -0.2654821122             5.4280586355            4.0747197795

 H262           -2.0947620223             5.4736225683            8.6821395551

 H263            0.8202265350             4.0745637534            5.8538146019

 H264           -2.0276037244             9.2612290397            3.5952607998

 H265           -0.5380514531             8.4670294152            4.1440828915

 C266           -5.2856348100#           -5.5126299307#           5.1332789332#

 C267           -4.4493717758            -5.4055912222            3.8504448256

 C268           -2.9547011607            -5.3528992778            4.1259058940

 O269           -2.2854466592            -4.3815194405            3.7620326655

 N270           -2.4178543193            -6.4245962150            4.7694446108

 H271           -5.2082701773            -6.5080407653            5.5877429586

 H272           -4.6872841195            -4.4933690852            3.2992377172

 H273           -4.6696184307            -6.2534821109            3.1883803860

 H274           -3.0321779324            -7.1004272834            5.2019958606

 H275           -1.4673028411            -6.3818965830            5.1400525778

 H276           -6.3460176720            -5.3465653063            4.9171222916

 H277           -4.9699828500            -4.7851546455            5.8843421364

 C278           -4.9857220139#           -2.1245000581#           5.3205940008#

 C279           -5.3414972868            -1.6214286180            6.6953917805

 O280           -4.5452889559            -1.3924002789            7.5839945333

 H281           -3.9844310024            -2.5596848718            5.2962980999

 H282           -4.9954586642            -1.2631403343            4.6358964000

 H283           -6.4241769275            -1.4401181640            6.8702919859

 H284           -5.7413738667            -2.8236090974            4.9541615860

 C285           -1.2156620018#            3.5767179922#          14.9580160087#

 C286           -2.0893995264             4.5134415979           14.1054479620

 C287           -1.4437539192             4.8101864826           12.7898781654

 N288           -0.5465280691             5.8504746773           12.6230548389

 C289           -1.4172676560             4.1203666923           11.6024705565

 C290           -0.0282066252             5.7617675328           11.3793331979

 N291           -0.5303450269             4.7212935048           10.7365014803

 H292           -0.2147263020             3.9961071733           15.1092490755

 H293           -2.2832421107             5.4519469902           14.6401357026

 H294           -3.0662730090             4.0479345516           13.9327456749

 H295           -0.2999882463             6.5489589405           13.3315450369

 H296           -1.9542488361             3.2263614278           11.3189713987

 H297            0.6863461576             6.4658645825           10.9782275107

 H298           -0.2040964819             4.2689656835            9.2868019585

 H299           -1.6677438227             3.4046400193           15.9424716034

 H300           -1.0995549393             2.6095677681           14.4664574349

 C301           -0.2371729940#            6.7063619895#          17.0391969610#

 C302            0.6168934680             6.9832843577           15.8207962000

 O303            0.1229019540             7.4765494215           14.8027969146

 N304            1.9335933235             6.6675401214           15.9009744133

 H305           -1.0818801720             6.0822344970           16.7353801980

 H306            0.2797839957#            6.2148220025#          17.8670170041#

 H307            2.3537405422             6.2926622747           16.7380743702

 H308            2.5308524793             6.8789395482           15.1125911041

 H309           -0.6498116755             7.6559984685           17.3943554654

309 atom structure with an oxyl radical

 Mn1             2.9306523399            -1.4076959311            3.7152601709

 Mn2             4.5672213676            -1.4774573696            8.6218446966

 Mn3             5.2293826376            -1.4522780057            5.3183871765

 Mn4             6.5167852691             0.0564281865            7.3081336657

 O5              3.5355673090            -1.1932273507            5.6884692253

 O6              5.6598479220             0.2974811295            5.7380327450

 O7              5.0377785347             0.3024828258            8.3283089609

 O8              5.8424251343            -1.7292321939            7.1948969808

 Ca9             3.3924469828             1.1841631182            6.7794571358

 C10             2.1876270142#            1.2855489347#          -1.5249169503#

 C11             1.3832973528             0.8863300578           -0.2923734926

 O12             1.0959173681            -0.2926928376           -0.0660042805

 C13             3.4991882514             0.4881362576           -1.5694428204

 O14             4.3118051759             0.8343093092           -0.4625582744

 H15             1.5881679685             1.0524144375           -2.4136884821

 H16             3.2539102286            -0.5813642425           -1.5657862678

 H17             4.0144677021             0.7124492822           -2.5169612908

 H18             4.6724463597             0.0074406421           -0.0721071385

 H19             2.4044347035             2.3602204605           -1.5357240254

 N20             0.9912754547             1.8920045380            0.5451512649

 C21             0.3771999152             1.6246667272            1.8387959720

 C22            -1.1329998348             1.8417503508            1.8302225449

 O23            -1.8269094855             1.7103388400            2.8202570285

 C24             1.0651133241             2.3695868694            2.9979243378

 C25             2.2036801625             1.5912538723            3.6747503948

 O26             2.7992616536             2.1348063478            4.6269001820

 O27             2.4183561854             0.4229319123            3.2036824282

 H28             1.3113767380             2.8302531031            0.3459973990

 H29             0.5055884629             0.5478156409            2.0043595661

 H30             0.3333392652             2.5878840602            3.7833816676

 H31             1.4525377462             3.3433366963            2.6743644069

 H32            -1.5886415827             2.0911205269            0.8503832179

 C33             1.0582597265#            1.1829963492#          13.4563129973#

 H34             0.0336292861             0.7923517792           13.4274134231

 H35             1.0202996021             2.2004819888           13.8545582577

 H36             1.6320941500             0.5697020800           14.1617300000

 C37             1.6989524852             1.1629143583           12.0743419888

 H38             2.7019792624             1.5999460738           12.1293625831

 H39             1.1122820251             1.7655394273           11.3730630945

 C40             1.8114664354            -0.2685788694           11.5338848502

 H41             0.8057021993            -0.6866268883           11.3845889276

 H42             2.3456663938            -0.9074448385           12.2417769944

 C43             2.5290990411            -0.2945612748           10.1999740772

 O44             2.1485514991             0.4160807671            9.2533421457

 O45             3.5626070703            -1.0864318056           10.1725244591

 C46             3.8991870906            -8.1359202542            8.3962021964

 H47             3.2828674302            -9.0072437697            8.1521457425

 H48             4.4929514440            -8.3748399802            9.2843456633

 C49             4.8810408812            -7.8538944099            7.2677737870

 O50             6.0186817077            -7.3999529702            7.5069848998

 C51             2.9391611925            -6.9452844942            8.6766711711

 H52             2.2902065163            -7.2364044455            9.5142390840

 H53             2.2788075819            -6.8037308297            7.8173763484

 C54             3.5920412722            -5.6332248971            8.9781415984

 N55             4.3539314933            -5.4014986030           10.1189226358

 H56             4.5599243921            -6.0760588739           10.8425463140

 C57             3.5849762451            -4.4326493101            8.3142785553

 H58             3.0845857255            -4.1572588343            7.4010885171

 C59             4.7771023416            -4.1180383465           10.1100553861

 H60             5.3749718958            -3.6503074612           10.8759276972

 N61             4.3307402832            -3.5098820703            9.0235320602

 N62             4.4340745997            -8.0824718091            6.0192331406

 H63             3.5554195141            -8.5920685704            5.9161369211

 C64             5.2069916465#           -7.7515172067#           4.8093070037#

 C65             6.5241252067            -8.5230511507            4.9209526050

 O66             7.6376019745            -8.0667266282            4.7309059760

 H67             6.3975531784            -9.5727399316            5.2435255671

 H68             4.6484843402            -8.1897677448            3.9769880677

 C69             5.3967953162            -6.2409848770            4.5900170741

 H70             6.0126188136            -6.1120521277            3.6951492075

 H71             5.9591267956            -5.8235393283            5.4267339601

 C72             4.0620520139            -5.5118435747            4.4275255003

 H73             3.5875747679            -5.7701506002            3.4775135425

 H74             3.3574179080            -5.8106004827            5.2139470869

 C75             4.1205795297            -3.9960783026            4.4846687758

 O76             3.1715344216            -3.3899232955            3.9019097234

 O77             5.0645893109            -3.4304950451            5.1282974447

 C78             9.1595115093#            0.1191579828#          11.4478227539#

 H79             9.8629323307            -0.1068888733           10.6339957412

 H80             9.7106935855            -0.0853083242           12.3783318263

 C81             7.8947588536            -0.7500309816           11.3792401686

 H82             7.1290338687            -0.3748646484           12.0732100297

 H83             8.1263427854            -1.7706876005           11.7061881914

 C84             7.1801236102            -0.8725267625           10.0299062054

 O85             7.6293188431            -0.2382898571            9.0195480835

 O86             6.1595050653            -1.6201636501           10.0144887336

 C87             8.9059858060             1.6083512485           11.4169350746

 O88             7.8155157813             2.1438809170           11.4031308737

 H89             9.8233492430             2.2353687116           11.4305719369

 C90             7.7935564421#            4.3107436534#           8.7015790214#

 H91             8.3130916299             5.2670843314            8.8252916084

 H92             7.0152933079             4.2486636499            9.4687634446

 H93             8.5044311058             3.5009237796            8.8772890786

 C94             7.1886309057             4.2052585934            7.2976087859

 H95             6.4945676430             5.0264697662            7.0977300828

 H96             7.9897021990             4.2465655323            6.5463774576

 C97             6.4300321879             2.9019460508            7.0973568179

 O98             5.2621781989             2.8983296585            6.6824627307

 O99             7.1283998555             1.8417023774            7.3984496597

 C100           11.5816617848#           -0.6170833945#           3.4808991845#

 H101           12.2835601801            -1.0353544936            4.2123920896

 H102           12.0435425334             0.2810157393            3.0556968477

 H103           11.4610750364            -1.3488420607            2.6743019081

 C104           10.2358934635            -0.2920496801            4.1289586614

 H105            9.5602094593             0.1343526213            3.3769937450

 H106           10.3556053425             0.4616895997            4.9161944253

 C107            9.5842454125            -1.5497196895            4.7316021727

 H108           10.2189735573            -1.9346173756            5.5392256654

 H109            9.4942676517            -2.3306922544            3.9721902588

 C110            8.2178099451            -1.2549721790            5.3187814127

 O111            7.1972881903            -1.7387004579            4.7313185717

 O112            8.1926838040            -0.5326733709            6.3665695041

 C113           10.8187241195#            4.2285681215#          -0.8150213082#

 H114           11.5562910407             3.5259942060           -0.4099754081

 H115           11.1811689515             5.2447768482           -0.6205944548

 H116           10.7831819619             4.0876224753           -1.9000675310

 C117            9.4363222428             4.0061413371           -0.1848222693

 H118            9.0919500147             2.9951113029           -0.4489406028

 H119            8.7111884963             4.7018789363           -0.6296572553

 C120            9.4389379813             4.1882048174            1.3423177092

 H121            9.8126211802             5.1925981080            1.5810911639

 H122           10.1353948163             3.4765285160            1.8051214896

 C123            8.0643508795             4.0442193030            2.0135494711

 H124            7.3597983765             4.7721141625            1.5853022474

 H125            8.1510259036             4.2535561071            3.0841727055

 N126            7.5351771364             2.6865647982            1.8734007699

 H127            7.6513770455             2.2289622445            0.9788952889

 C128            6.7964174446             2.0113930703            2.7761785184

 N129            6.2698289417             2.6181565193            3.8502974141

 H130            5.8621285228             2.0381374956            4.5867648997

 H131            6.0087035145             3.5930254623            3.8362113786

 N132            6.6090508125             0.7058988354            2.6171736014

 H133            7.2480677868             0.0998844541            2.0991361248

 H134            5.8672178815             0.2181156265            3.1326577986

 C135           11.8423975709#           -5.3930895355#           7.2492549606#

 H136           11.3727501275            -6.3703753142            7.0948952607

 H137           12.9078748499            -5.5655847736            7.4321448296

 H138           11.7433967223            -4.8176550936            6.3230938002

 C139           11.2082302406            -4.6476482392            8.4333818976

 H140           11.7237551386            -3.6960961725            8.6030103047

 H141           11.3322039417            -5.2368140573            9.3518282768

 C142            9.7589905960            -4.3575459111            8.2078772754

 N143            8.8207313781            -5.3597545809            7.9977951779

 C144            9.0724748689            -3.1760482322            8.1107443639

 H145            9.3963286113            -2.1510179143            8.1953049134

 C146            7.6243504970            -4.8048927369            7.7770122779

 H147            6.7196673839            -5.3599706230            7.5795930169

 N148            7.7530946896            -3.4841614594            7.8431607232

 H149            6.9820190299            -2.8007807187            7.6303274213

 C150            6.0989355952#           -4.2351882501#          -1.2221973962#

 H151            5.5184951085            -4.7085972051           -2.0282544247

 C152            7.5233420395#           -4.0871570785#          -1.7388095028#

 H153            7.5919055082            -3.7164118647           -2.7845900855

 O154            8.5480225080            -4.3124966846           -1.1245686677

 H155            5.6950849579            -3.2157613516           -1.1615071038

 C156            5.9322718850            -4.9878613429            0.0886474668

 H157            6.2184600064            -6.0338508655           -0.0301024336

 H158            6.5972758656            -4.5686122586            0.8523491789

 C159            4.4893017457            -4.9002840570            0.5944020365

 O160            3.9041310093            -3.7808641975            0.4576400209

 O161            3.9639085168            -5.9283260136            1.1101597855

 C162           -1.8288746693#          -11.9867167443#           4.2485681998#

 H163           -1.3833528517           -12.9523423581            3.9905163374

 H164           -2.8542105975           -12.1476071543            4.6063528490

 C165           -1.0241498411           -11.3382822762            5.3437423053

 H166           -1.5061228727           -10.4565084900            5.8219731116

 O167            0.1024731153           -11.6632363570            5.6710954686

 C168           -1.8457685713           -11.0338168367            3.0229168192

 H169           -2.6089373610           -11.3853748169            2.3177916847

 H170           -2.1762816175           -10.0382350423            3.3545402715

 C171           -0.4805561200           -10.9509004643            2.3228410103

 H172            0.3207412945           -10.7622984471            3.0455131302

 H173           -0.2527631844           -11.9361095298            1.8956295649

 C174           -0.3944498572            -9.9112204907            1.1932716465

 H175            0.5478221507           -10.0619282098            0.6523688009

 H176           -1.1974037048           -10.0858235220            0.4639089657

 C177           -0.4798157852            -8.4389254171            1.6086831715

 H178           -0.4913930279            -7.8049264996            0.7180949341

 H179           -1.3920949449            -8.2192184453            2.1706687570

 N180            0.6831714099            -7.9696995134            2.4365185920

 H181            1.0078732779            -7.0120424481            2.0545925580

 H182            0.4465136787            -7.7967138778            3.4234314385

 H183            1.4907074360            -8.6425776076            2.4649500491

 Cl184           0.7266559504            -6.7855352685            5.5403125174

 O185            0.9849090456            -1.7403461568            4.0253706206

 O186            4.7364687873            -1.1715925730            3.5729541798

 O187            1.1774479321             0.3172480641            6.5645411744

 H188            0.3678411804             0.2896082140            6.0021189787

 H189            1.2526768067            -0.5357409878            7.0246022292

 O190            2.7838395301             3.0933277927            8.1864802009

 H191            3.1700995795             3.0027148337            9.0749410971

 H192            1.8327458853             3.3188622134            8.2986902656

 O193            5.0038831791             2.0382950054           10.4648061492

 H194            5.0301957468             1.3770612359            9.7329736683

 H195            5.9421528797             2.2451820545           10.6281173004

 O196            2.5309402568            -1.9127510343            1.6691735433

 H197            2.7929610891            -2.7680353949            1.2454401486

 O198            3.1925096414            -1.5209335929            7.6576651320

 H199            1.9766783539            -1.3857868384            1.0566433913

 H200            0.6791169540            -2.6970930670            3.9422883467

 H201            0.2952996525            -1.1639305576            4.4327203614

 H202            8.9732133243            -6.3902481742            7.8840108988

 O203           -0.6288587224             1.0769239315            9.0985839911

 H204            0.3103480623             0.7886124416            9.1067681955

 H205           -0.5706711658             1.9673217517            8.7021289202

 O206           -0.9786294925            -0.0710903344            4.9892430038

 H207           -1.5896738585            -0.0581506646            5.7939029398

 H208           -1.2854531624             0.6183606501            4.3672678535

 O209           -2.3363778944            -0.0097773129            7.2353636922

 H210           -2.7549149041            -0.8479382907            7.5002863370

 H211           -1.7285586486             0.2617716530            7.9716590882

 O212            9.1195278211            -4.2661322689            1.6830730842

 H213            8.6401799367            -3.4117699784            1.7739486339

 H214            9.1399711991            -4.4042790343            0.7166932160

 O215            1.2695168831            -5.6024480339            1.4133669895

 H216            2.2525435108            -5.5845691077            1.2742698620

 H217            1.0671786612            -4.9490139438            2.1203615214

 O218            0.3138618217            -4.2260090279            3.6722645205

 H219            0.6376951248            -4.8348332673            4.3664142109

 H220           -0.6720954712            -4.3351692298            3.6689854793

 O221            5.2915761085            -1.5059996027            0.7793993650

 H222            4.8043381368            -2.3416786256            0.5311597621

 H223            4.9303491308            -1.3238571474            1.6741950660

 O224            7.7222126956            -1.8390449718            1.9366187832

 H225            6.8909202896            -1.8743242833            1.3958487307

 H226            7.4218547528            -1.9568725017            2.8635352777

 O227            8.7390882983            -8.0363375999            7.4819861112

 H228            7.7693450432            -8.0451189285            7.6267116692

 H229            8.8066778775            -8.1565506720            6.5163417302

 O230            2.6032181189            -9.8107423601            3.0570743048

 H231            2.4791197424            -9.8570845517            4.0292941672

 H232            3.5428424573           -10.0714574217            2.8754319293

 O233            5.7585920102            -8.0795691159            1.3347891615

 H234            5.0496588201            -7.3986096183            1.2583082056

 H235            6.5647914833            -7.5793208616            1.6093911550

 O236            7.9027392888            -6.6446093310            2.2668347808

 H237            8.0930010009            -6.9126383766            3.1798489362

 H238            8.3231436728            -5.7597314819            2.1213514951

 O239            2.0037230270            -9.5863471288            5.8030799950

 H240            1.4432143738            -8.7756709512            5.8056972232

 H241            1.4327969786           -10.3456751098            6.0372564629

 O242            5.2608950496           -10.2483002098            2.7480689233

 H243            5.5622077437           -11.0254053103            2.2535603824

 H244            5.4778482713            -9.4582714122            2.1638386913

 O245            5.1422863608             0.5442606255           12.8802084310

 H246            4.9797525038             0.9567588302           12.0026821699

 H247            5.4523304984             1.2890207842           13.4176394668

 C248           -1.6084959741#            8.5839729455#           4.3490290361#

 C249           -1.9864400915             7.1152423532            4.0831852914

 C250           -1.4614884055             6.1728319835            5.1466813365

 C251           -2.1195023362             6.0498590032            6.3791213522

 C252           -0.2852168655             5.4339446536            4.9614468430

 C253           -1.6214653411             5.2353569841            7.3940099982

 C254            0.2333283494             4.6108311438            5.9646555094

 C255           -0.4353361267             4.5181417728            7.1892979136

 O256            0.0885918511             3.7217211067            8.1780589306

 H257           -2.0053163023             8.9207041064            5.3132252213

 H258           -1.6026950077             6.8126634814            3.1001630984

 H259           -3.0798161693             7.0326117324            4.0240780229

 H260           -3.0425112597             6.6000709766            6.5498639852

 H261            0.2473222033             5.5123051697            4.0153015244

 H262           -2.1452887567             5.1516019206            8.3407849286

 H263            1.1564387441             4.0597740919            5.8117477876

 H264           -2.0086789737             9.2423173087            3.5690235546

 H265           -0.5201244202             8.7091797432            4.3762394644

 C266           -5.2856347283#           -5.5126299197#           5.1332788950#

 C267           -4.5325369438            -5.4534776177            3.7951670553

 C268           -3.0272533613            -5.4360544323            3.9996525821

 O269           -2.3667014894            -4.4262564345            3.7308756885

 N270           -2.4702244351            -6.5711310161            4.4984794151

 H271           -5.1806037926            -6.4924443157            5.6138671335

 H272           -4.7803607611            -4.5429775578            3.2455556179

 H273           -4.8152637833            -6.3077055535            3.1665913882

 H274           -3.0738116585            -7.2991265512            4.8551618083

 H275           -1.5180096770            -6.5526080860            4.8695156372

 H276           -6.3565330918            -5.3430111551            4.9810440496

 H277           -4.9129736724            -4.7641364015            5.8361533616

 C278           -4.9857219588#           -2.1245000104#           5.3205940553#

 C279           -5.1305670145            -2.0944860493            6.8184126814

 O280           -4.2111231525            -2.1538504540            7.6124602242

 H281           -4.0101343727            -2.5095924989            5.0138180604

 H282           -5.0827947443            -1.0921819851            4.9522821347

 H283           -6.1705742277            -1.9927447651            7.1962803720

 H284           -5.7997546047            -2.6956072310            4.8637411989

 C285           -1.2156620000#            3.5767179820#          14.9580159915#

 C286           -2.1087834363             4.3645014315           13.9841456752

 C287           -1.4412909464             4.6022888547           12.6662368230

 N288           -0.5822585357             5.6690673239           12.4649573536

 C289           -1.4127248259             3.8942870836           11.4889629532

 C290           -0.0827276472             5.5750723464           11.2130449101

 N291           -0.5624790584             4.5098492438           10.5937020253

 H292           -0.2484546022             4.0714380749           15.0988375390

 H293           -2.3865246355             5.3290885168           14.4275306625

 H294           -3.0430949828             3.8164716963           13.8190802414

 H295           -0.3241519498             6.3628176008           13.1718223764

 H296           -1.9274623669             2.9801167775           11.2292723479

 H297            0.6064739873             6.2921581070           10.7907085790

 H298           -0.2145817604             4.0478157713            9.1059737286

 H299           -1.6975468990             3.4835508993           15.9389898480

 H300           -1.0254041418             2.5709998186           14.5806752397

 C301           -0.2371729678#            6.7063619671#          17.0391969230#

 C302            0.5842374084             6.8861393323           15.7786798672

 O303            0.0546292131             7.2448069944           14.7240135268

 N304            1.9180124929             6.6556225925           15.8753792301

 H305           -1.1285577286             6.1259785062           16.7887653743

 H306            0.2797839810#            6.2148220158#          17.8670170212#

 H307            2.3614791499             6.3977085354           16.7440220329

 H308            2.5012521232             6.8180396730           15.0654298220

 H309           -0.5766038746             7.6922591565           17.3748796519

340 atom structure with an OH

 Mn1             2.9037776777            -1.4299586885            3.7986724253

 Mn2             4.5151537726            -1.5138148450            8.7398344635

 Mn3             5.2540169215            -1.3853537364            5.2467594629

 Mn4             6.3737774312             0.0940377322            7.3532039638

 O5              3.4626972548            -1.1196219336            5.4984493039

 O6              5.5906773962             0.3216435765            5.7612870474

 O7              4.8502237708             0.2542740628            8.3434967901

 O8              5.7589604231            -1.7148244088            7.2500274483

 Ca9             3.0805538192             1.1256696704            6.8666023924

 C10             2.1876270786#            1.2855489124#          -1.5249168569#

 C11             1.5372071170             1.0013233984           -0.1787361241

 O12             1.1821405980            -0.1332748950            0.1537312128

 C13             3.5110112521             0.5097699327           -1.6237182400

 O14             4.4013290091             0.9673944981           -0.6228572975

 H15             1.4988419889             0.9539210089           -2.3112408216

 H16             3.2971306896            -0.5610832796           -1.5096927238

 H17             3.9409515229             0.6626679354           -2.6259515987

 H18             4.7637612314             0.1853934116           -0.1486089985

 H19             2.3757315862             2.3557205177           -1.6664636428

 N20             1.3701148210             2.0897346851            0.6222706291

 C21             0.6878050097             2.0736005006            1.9121562624

 C22            -0.5550490803             2.9410794110            1.6977018706

 O23            -0.7489388549             4.0195855198            2.2153568463

 C24             1.5518123186             2.5509959950            3.0753725098

 C25             2.5316662964             1.5354758279            3.6630252028

 O26             3.2884925059             1.9243448923            4.5754084001

 O27             2.4673441770             0.3502509315            3.1780436419

 H28             1.7511178268             2.9713003169            0.3046574515

 H29             0.3720670241             1.0380944792            2.0737018379

 H30             0.8914587972             2.8200275573            3.9070567068

 H31             2.1057945338             3.4627614932            2.8256124403

 H32            -1.2642624558             2.5335563537            0.9474294878

 C33             1.0582598445#            1.1829964025#          13.4563130462#

 H34             0.0042660691             0.8816768138           13.4126525455

 H35             1.1079454262             2.1770852905           13.9111102638

 H36             1.5792297668             0.4866447145           14.1245966336

 C37             1.6915479586             1.1811844851           12.0704880672

 H38             2.7391310324             1.4926606099           12.1450718912

 H39             1.1796637089             1.8912476611           11.4121884935

 C40             1.6200178564            -0.2190084934           11.4400979112

 H41             0.5749347992            -0.4707902358           11.2190375318

 H42             2.0198226116            -0.9698751070           12.1268987477

 C43             2.4019638604            -0.2641579420           10.1454084604

 O44             2.0565392523             0.4207457692            9.1674125199

 O45             3.4541504180            -1.0388016200           10.2080923486

 C46             4.1181597116            -8.2215938403            8.4629653109

 H47             3.5049160690            -9.0994299361            8.2341797366

 H48             4.7780278494            -8.4754934239            9.2990663329

 C49             5.0169206367            -7.8858742982            7.2809221696

 O50             6.1513773463            -7.3959144561            7.4566398510

 C51             3.1529597349            -7.0660336004            8.8549673301

 H52             2.5769974726            -7.4052911979            9.7270087715

 H53             2.4275812448            -6.9165426698            8.0507434902

 C54             3.7828520394            -5.7422974192            9.1532495196

 N55             4.6202106271            -5.5007459853           10.2385343986

 H56             4.9102591331            -6.1791184810           10.9291426174

 C57             3.6624310692            -4.5258384372            8.5341598125

 H58             3.0632200983            -4.2571340113            7.6813200421

 C59             4.9745923879            -4.1947204792           10.2357175127

 H60             5.6071610550            -3.7202226724           10.9687011869

 N61             4.4134046547            -3.5801790300            9.2079305970

 N62             4.5062212730            -8.1128415384            6.0555350078

 H63             3.6362782137            -8.6432365542            5.9931593063

 C64             5.2069915939#           -7.7515171093#           4.8093069903#

 C65             6.5462006226            -8.4948279024            4.8446506534

 O66             7.6414010792            -8.0060110870            4.6322142934

 H67             6.4546596304            -9.5576011216            5.1343922762

 H68             4.6138067329            -8.1910053017            4.0028106572

 C69             5.3678214749            -6.2311397404            4.5982805205

 H70             5.9435243338            -6.0877217336            3.6800121600

 H71             5.9701480748            -5.8238173004            5.4117029862

 C72             4.0338324088            -5.4823049222            4.5040504580

 H73             3.4926788780            -5.7467330044            3.5936812818

 H74             3.3756432998            -5.7488898685            5.3410127871

 C75             4.1361123099            -3.9647053722            4.5265871149

 O76             3.1780492534            -3.3503470179            3.9430422413

 O77             5.0895837127            -3.3977610816            5.1355441930

 C78             9.1595115487#            0.1191579555#          11.4478228097#

 H79             9.8316093664            -0.1205440138           10.6118599756

 H80             9.7309995794            -0.1146832214           12.3591159213

 C81             7.8686369545            -0.7167577666           11.4115416910

 H82             7.1363219115            -0.3408179859           12.1413378552

 H83             8.0881968209            -1.7479832502           11.7125801807

 C84             7.1058541890            -0.8012186218           10.0860605696

 O85             7.5117976471            -0.1496378859            9.0768700494

 O86             6.0765653878            -1.5474999531           10.0948134662

 C87             8.9564178058             1.6154024026           11.4452328221

 O88             7.8850600273             2.1861905194           11.4940381888

 H89             9.8932763017             2.2113354821           11.4228573205

 C90             7.7935564907#            4.3107436794#           8.7015790294#

 H91             8.2156959996             5.3167698170            8.8033027535

 H92             7.3474282833             4.0330278372            9.6604244594

 H93             8.6077728291             3.6113228608            8.4931644105

 C94             6.7500881276             4.2873964604            7.5766135702

 H95             5.9421370880             5.0007774185            7.7617083049

 H96             7.2208907912             4.5731251994            6.6237644006

 C97             6.0890979822             2.9345342066            7.3368168669

 O98             4.9034504192             2.8682379389            6.9835529988

 O99             6.8787395716             1.9079646962            7.5085596409

 C100           11.5816617663#           -0.6170833903#           3.4808991628#

 H101           12.2959240635            -0.9825109354            4.2285907195

 H102           12.0352013089             0.2454089305            2.9806636684

 H103           11.4377313341            -1.4061265897            2.7344267192

 C104           10.2427579847            -0.2327859340            4.1285601327

 H105            9.5587558891             0.1306491178            3.3532318915

 H106           10.3816591399             0.5841685433            4.8471847311

 C107            9.5916228083            -1.4338177222            4.8467775786

 H108           10.2268961751            -1.7397967855            5.6863814089

 H109            9.5128038625            -2.2790441949            4.1571693146

 C110            8.2079984003            -1.1358007450            5.4059414770

 O111            7.2117547704            -1.6562828658            4.7963753061

 O112            8.1269694161            -0.4127094799            6.4452162835

 C113           10.8187241044#            4.2285681162#          -0.8150213109#

 H114           11.5816830643             3.6345464579           -0.2986224470

 H115           11.1181426175             5.2817440929           -0.7597709570

 H116           10.8197498009             3.9333291986           -1.8690750009

 C117            9.4276113842             4.0150041275           -0.1883112510

 H118            9.1482152538             2.9586815119           -0.3173453219

 H119            8.6807552879             4.5943638497           -0.7485682909

 C120            9.3558421942             4.4109557310            1.2994501441

 H121            9.6647996039             5.4594788560            1.4013062833

 H122           10.0745830888             3.8179146111            1.8810093501

 C123            7.9671693166             4.2855331372            1.9519351589

 H124            7.2314314347             4.8835204625            1.3952350559

 H125            8.0006450953             4.6823347518            2.9716138525

 N126            7.5433997467             2.8860003548            2.0247839066

 H127            7.8498648121             2.2790193683            1.2764679574

 C128            6.5968696093             2.3600639736            2.8353196268

 N129            5.8807851065             3.1124090552            3.6747482351

 H130            5.2001479422             2.6537653751            4.2813524855

 H131            5.7843943105             4.1179131702            3.5740000224

 N132            6.4084093476             1.0417094440            2.7956877117

 H133            7.0892715579             0.4018896891            2.3825387750

 H134            5.6624111371             0.5866517044            3.3226455057

 C135           11.8423975767#           -5.3930895508#           7.2492549392#

 H136           11.3780268373            -6.3836489376            7.2006544866

 H137           12.9109377539            -5.5402448607            7.4355075677

 H138           11.7295863255            -4.9131594274            6.2715654126

 C139           11.2205695400            -4.5353195116            8.3625249361

 H140           11.7411378785            -3.5742802891            8.4375835702

 H141           11.3555888891            -5.0366448069            9.3307800997

 C142            9.7671623847            -4.2573752803            8.1364332568

 N143            8.8415282756            -5.2660134210            7.9022112111

 C144            9.0601501454            -3.0843259979            8.0881969739

 H145            9.3705215699            -2.0581326692            8.2030797503

 C146            7.6348518931            -4.7221079937            7.7125927057

 H147            6.7370292327            -5.2837175446            7.5054421196

 N148            7.7417310194            -3.4024707431            7.8242300792

 H149            6.9492053468            -2.7278548708            7.6360540581

 C150            6.0989356649#           -4.2351883292#          -1.2221974906#

 H151            5.5596047502            -4.8635761722           -1.9464516212

 C152            7.5233420035#           -4.0871570102#          -1.7388095312#

 H153            7.5962542415            -3.8672239529           -2.8254843549

 O154            8.5439659854            -4.1672760852           -1.0828913341

 H155            5.6349540839            -3.2423737284           -1.3183321012

 C156            5.9602486043            -4.7760626079            0.1917689627

 H157            6.2889391485            -5.8132369255            0.2284053337

 H158            6.6087804088            -4.2132235942            0.8721317226

 C159            4.5185346924            -4.6652957361            0.6920939145

 O160            3.9438958905            -3.5396989380            0.5219769464

 O161            3.9862170670            -5.6729700232            1.2339858541

 C162           -1.8288746287#          -11.9867167259#           4.2485682366#

 H163           -1.4125737377           -12.9396854339            3.9075460066

 H164           -2.8468006490           -12.1501421070            4.6242323619

 C165           -0.9743461323           -11.4318246245            5.3587330477

 H166           -1.4421035256           -10.6146688618            5.9510588763

 O167            0.1771221652           -11.7593956255            5.5831034382

 C168           -1.8552907014           -10.9494560769            3.0921888129

 H169           -2.6442058661           -11.2366167793            2.3860849214

 H170           -2.1547151820            -9.9728199331            3.5001317968

 C171           -0.5080857115           -10.8442254145            2.3604248758

 H172            0.3159547552           -10.7176046849            3.0707077604

 H173           -0.3112861384           -11.8029846930            1.8631157064

 C174           -0.4371024231            -9.7325273966            1.3001529932

 H175            0.4820955011            -9.8628832283            0.7156339251

 H176           -1.2675342347            -9.8441104707            0.5892966617

 C177           -0.4841383881            -8.2922326897            1.8193313499

 H178           -0.5337047931            -7.5974002280            0.9767729779

 H179           -1.3640885856            -8.1062148390            2.4404744149

 N180            0.7251399972            -7.8897329067            2.6177589007

 H181            1.0731935538            -6.9397564482            2.2405687661

 H182            0.5246055764            -7.7203273640            3.6153873468

 H183            1.5038255512            -8.5946116152            2.6115712996

 Cl184           0.8019141141            -6.8830999979            5.7887831026

 O185            1.1997318990            -1.6170065915            4.3106556159

 O186            4.8234602141            -1.1752679777            3.5419601279

 O187            0.8937748775             0.5213795965            6.0352525907

 H188            0.0361405678             0.9645620907            5.7999421819

 H189            0.8707738738            -0.3338576055            5.5457981821

 O190            2.2346834929             3.2482016009            7.6872101562

 H191            2.8683479540             3.9717820229            7.8546540461

 H192            1.3538702632             3.5206574755            8.0190096410

 O193            5.1129303699             1.9038151900           10.6178875646

 H194            4.9879874009             1.3215854300            9.8372757432

 H195            6.0758961507             2.0270901612           10.6902235126

 O196            2.4963505252            -1.8812996831            1.8899923372

 H197            2.8715267690            -2.6686019630            1.3954979957

 O198            3.0184835520            -1.8057727677            7.8507650345

 H199            1.9756120227            -1.2703332903            1.3192169489

 H200            0.9019403072            -2.5618484710            4.1972472524

 H201            3.1338820310            -1.7818276348            6.8605902099

 H202            9.0132947653            -6.2895355386            7.7552298746

 O203           -0.7067437346             1.2571769527            9.3272764300

 H204            0.1870860246             0.8716299215            9.2036646250

 H205           -0.6368213512             2.1428776604            8.9215314475

 O206           -1.5216381114             1.5415364477            5.3440902332

 H207           -2.0569208290             1.2155105663            6.1203842073

 H208           -1.6308628450             2.5061154239            5.3662697182

 O209           -2.7774925349             0.7845343534            7.5987548965

 H210           -3.2951194606            -0.0330876299            7.6891816102

 H211           -2.0631995838             0.7608277207            8.2809583624

 O212            9.0725584307            -3.9478692434            1.7441720526

 H213            8.6301244724            -3.0734221803            1.8189442840

 H214            9.0899606058            -4.1096429603            0.7810233196

 O215            1.2988507713            -5.5034653319            1.6374826747

 H216            2.2719193187            -5.4271365702            1.4622738255

 H217            1.0831824524            -4.8814804834            2.3722287965

 O218            0.3991917195            -4.1939337313            3.9282046294

 H219            0.6892984890            -4.8179950731            4.6223026711

 H220           -0.5847135187            -4.2744115137            3.9080641122

 O221            5.3137440040            -1.2563984274            0.7856811633

 H222            4.8540164371            -2.1102084726            0.5514585027

 H223            4.9821536542            -1.0892671529            1.6965272844

 O224            7.7344353987            -1.4549232673            2.0165463088

 H225            6.9332258147            -1.5112730700            1.4343781617

 H226            7.4046662550            -1.6768859334            2.9136705686

 O227            8.8696475797            -7.9375045543            7.3352842047

 H228            7.9089077035            -7.9944964977            7.5247489130

 H229            8.8962762537            -8.0424291281            6.3659864138

 O230            2.6202719964            -9.8011099425            3.1326534620

 H231            2.5326755206            -9.8960172950            4.1056579716

 H232            3.5560703383           -10.0316738693            2.9009257831

 O233            5.7453252771            -7.8743675945            1.4154650172

 H234            5.0439077805            -7.1845539531            1.3599233729

 H235            6.5614728148            -7.3825294143            1.6713445023

 O236            7.9201798960            -6.4053017792            2.2794945551

 H237            8.1127246934            -6.7022277494            3.1829514331

 H238            8.2941625966            -5.4962224765            2.1768201452

 O239            2.0989196626            -9.7080662900            5.9210145758

 H240            1.5158733191            -8.9127807286            5.9169140212

 H241            1.5132668064           -10.4786622242            6.0674800120

 O242            5.2725608689           -10.1624690026            2.6613826882

 H243            5.5590746829           -10.9022081917            2.1048791406

 H244            5.4749356877            -9.3337593053            2.1336901313

 O245            5.2301635837             0.5037734338           13.0571641946

 H246            5.0710406913             0.8678158124           12.1562446022

 H247            5.6500254660             1.2492202134           13.5135337449

 C248           -1.6084959763#            8.5839729658#           4.3490290364#

 C249           -2.6409735358             7.4409946370            4.4273238222

 C250           -2.2053282678             6.3857045212            5.4194205253

 C251           -2.6737995221             6.3792183758            6.7398851179

 C252           -1.2177751489             5.4539304903            5.0687505352

 C253           -2.1366570340             5.5243749821            7.7040347964

 C254           -0.6603735169             4.5994426427            6.0212537955

 C255           -1.0861232739             4.6648852685            7.3543811175

 O256           -0.4179745797             3.9186726322            8.2859241058

 H257           -1.4874251124             9.0651799645            5.3267544803

 H258           -2.7667508432             6.9962092658            3.4321172670

 H259           -3.6175010329             7.8502522875            4.7144664064

 H260           -3.4627032522             7.0698058896            7.0306288139

 H261           -0.8660446778             5.3984825697            4.0436099534

 H262           -2.5001605773             5.5484292558            8.7252440487

 H263            0.1553546004             3.9292577668            5.7667102797

 H264           -1.9168967565             9.3512251488            3.6289857370

 H265           -0.6298310248             8.2010200914            4.0374787764

 C266           -5.2856347717#           -5.5126298551#           5.1332789353#

 C267           -4.4799506992            -5.4884236812            3.8227053715

 C268           -2.9817049420            -5.4950059452            4.0779409832

 O269           -2.3036513588            -4.4903342486            3.8490833103

 N270           -2.4464875359            -6.6526890421            4.5655471885

 H271           -5.2440066838            -6.4981939509            5.6123200993

 H272           -4.6863665113            -4.5779811741            3.2556463856

 H273           -4.7600768637            -6.3433247705            3.1942323471

 H274           -3.0711204368            -7.3491356550            4.9501678528

 H275           -1.5132991742            -6.6251745393            4.9806439722

 H276           -6.3411943730            -5.2963771547            4.9394709204

 H277           -4.9088911174            -4.7830947778            5.8524720146

 C278           -4.9857220138#           -2.1245000582#           5.3205939976#

 C279           -5.4814400968            -1.5259328234            6.6099605404

 O280           -4.8046716287            -1.3198082751            7.5986359534

 H281           -4.0035433811            -2.5877546469            5.4287980628

 H282           -4.9063618860            -1.3143372193            4.5804413414

 H283           -6.5580314469            -1.2499450920            6.6210722839

 H284           -5.7218326205            -2.8304224366            4.9259931328

 C285           -1.2156619978#            3.5767179907#          14.9580159920#

 C286           -2.1686124708             4.4823352714           14.1609269633

 C287           -1.6094639307             4.8002831674           12.8120952832

 N288           -0.7455943318             5.8612188414           12.6050778890

 C289           -1.6653636676             4.1412866209           11.6090538536

 C290           -0.3215007550             5.8100727135           11.3232739665

 N291           -0.8586571512             4.7803367186           10.6921757401

 H292           -0.2235727179             4.0312501024           15.0550383647

 H293           -2.3604236700             5.4139721137           14.7084683613

 H294           -3.1380192488             3.9855470638           14.0432767667

 H295           -0.4421111345             6.5290882202           13.3203886658

 H296           -2.2073123792             3.2439338401           11.3468032052

 H297            0.3628269339             6.5234131360           10.8880839488

 H298           -0.6073118729             4.2805444915            9.2402693047

 H299           -1.6048992480             3.3860203271           15.9656262818

 H300           -1.0924617883             2.6155778447           14.4567934997

 C301           -0.2371729983#            6.7063619687#          17.0391969392#

 C302            0.6367217553             6.9754806168           15.8295025756

 O303            0.1624701869             7.4172534561           14.7785718491

 N304            1.9618063080             6.7192001371           15.9643228413

 H305           -1.0785610743             6.0805455199           16.7308454760

 H306            0.2797840007#            6.2148220174#          17.8670170098#

 H307            2.3678878888             6.3974594055           16.8300030207

 H308            2.5779062552             6.9211006506           15.1883043643

 H309           -0.6492117215             7.6576638352           17.3912652357

 C310           -1.7760929928#           -3.1799650231#           9.5045650345#

 C311           -1.8822317425            -3.3488774130            7.9903677608

 C312           -0.9253067941            -2.4051707389            7.2527826303

 C313           -1.6295343330            -4.8105166838            7.6139403905

 H314           -2.9083103503            -3.0871163380            7.6976459351

 H315           -1.1950205552            -1.3570362580            7.4165364316

 H316           -0.9400492382            -2.5885726141            6.1719381196

 H317            0.1086070887            -2.5389885149            7.5967305943

 H318           -1.6891820163            -4.9526293932            6.5322981580

 H319           -2.3576132843            -5.4793473412            8.0926620345

 H320           -0.6282919318            -5.1337799400            7.9211143198

 H321           -2.0586670075#           -2.1742879894#           9.8440009624#

 H322           -0.7470900261            -3.3669620692            9.8420396749

 H323           -2.4290834764            -3.8903498057           10.0287221598

 C324            4.0257129819#            7.3892939799#           5.0853360114#

 C325            5.0856518131             7.0994539735            4.0536073288

 O326            5.4134623339             6.0077637115            3.6284148727

 C327            2.9366313844             6.3412995547            5.3383666736

 C328            2.0193162613             6.7911397278            6.4865904719

 C329            2.6905690046             6.7325876726            7.8551526128

 O330            3.5356634708             5.8859314465            8.1503277474

 N331            2.2683946326             7.6551496352            8.7671300811

 H332            4.5761240111#            7.5099999910#           6.0289889947#

 H333            3.3933438432             5.3828914770            5.6014443041

 H334            2.3319770668             6.1849454785            4.4382503978

 H335            1.1587986052             6.1152356627            6.5403253826

 H336            1.6120421718             7.7924809077            6.2958307048

 H337            1.5985285439             8.3757484985            8.5426380461

 H338            2.6842186722             7.6423118618            9.6888603254

 H339            5.6345186715             7.9985522344            3.6994086498

 H340            3.5969245648             8.3748035555            4.8567102649

340 atom structure with an oxyl radical

 Mn1             2.8613675367            -1.4261319303            3.7736731565

 Mn2             4.5121044192            -1.4434299398            8.6321590538

 Mn3             5.1868385880            -1.3931528206            5.3361918545

 Mn4             6.4010353359             0.1639836238            7.3243761436

 O5              3.4839980270            -1.1884229419            5.7181237213

 O6              5.5412847154             0.3641251446            5.7583035362

 O7              4.9229978588             0.3464603783            8.3465205181

 O8              5.8122021061            -1.6542857125            7.2074813098

 Ca9             3.1848194467             1.1497769724            6.8523767146

 C10             2.1876270634#            1.2855489256#          -1.5249169857#

 C11             1.3542998097             0.8632054685           -0.3251400041

 O12             1.0618601345            -0.3177022626           -0.1187113669

 C13             3.5316579631             0.5424464053           -1.5095143246

 O14             4.2691706993             0.9141947075           -0.3610797523

 H15             1.6315601199             1.0251161283           -2.4340463563

 H16             3.3329793491            -0.5368334799           -1.5252568039

 H17             4.0844540837             0.7954226711           -2.4281754929

 H18             4.6235721855             0.0957776789            0.0563230586

 H19             2.3620461246             2.3678522929           -1.5338840546

 N20             0.9282952564             1.8639990825            0.5017019453

 C21             0.2394352777             1.5840221693            1.7509075772

 C22            -1.2618062654             1.8350316523            1.6399464039

 O23            -2.0374871194             1.6690436783            2.5627133646

 C24             0.8611108631             2.3063452801            2.9588458007

 C25             1.9758351002             1.5309990989            3.6827850816

 O26             2.5044064952             2.0788703335            4.6697290241

 O27             2.2318578176             0.3708871283            3.2129152640

 H28             1.3022777439             2.7914484645            0.3528616279

 H29             0.3454442672             0.5042873187            1.9100083773

 H30             0.0854950534             2.4880861138            3.7086171060

 H31             1.2500992808             3.2926073136            2.6768652177

 H32            -1.6316894452             2.1562948889            0.6451062063

 C33             1.0582597314#            1.1829963145#          13.4563129394#

 H34             0.0230974838             0.8192349433           13.4591236568

 H35             1.0662067584             2.1932378539           13.8759583501

 H36             1.6401098522             0.5399897078           14.1286911472

 C37             1.6493214968             1.1687268036           12.0516458391

 H38             2.6626172011             1.5861777917           12.0681403808

 H39             1.0546350802             1.7919379133           11.3750085322

 C40             1.7088234547            -0.2611625986           11.4973219729

 H41             0.6899517214            -0.6477919507           11.3551274852

 H42             2.2355079427            -0.9184492799           12.1940344012

 C43             2.4161635643            -0.2906414606           10.1590018721

 O44             2.0104625741             0.3896592757            9.2029878196

 O45             3.4747051391            -1.0552728943           10.1529615468

 C46             3.9718217397            -8.1115362128            8.4224968730

 H47             3.3685213962            -8.9986206464            8.2028897408

 H48             4.5832024384            -8.3260800838            9.3049515762

 C49             4.9292266430            -7.8299179001            7.2735982743

 O50             6.0604662343            -7.3492969478            7.4875366289

 C51             2.9950835469            -6.9343260799            8.6990702359

 H52             2.3487107998            -7.2341333179            9.5357335231

 H53             2.3354395092            -6.8022633060            7.8381979209

 C54             3.6284138786            -5.6136931806            9.0037467959

 N55             4.3772011201            -5.3658803444           10.1509495798

 H56             4.5828876712            -6.0316816954           10.8827122075

 C57             3.6101535575            -4.4157211547            8.3359779483

 H58             3.1108353335            -4.1467343432            7.4192846059

 C59             4.7783324960            -4.0747778989           10.1418632802

 H60             5.3581200051            -3.5933684699           10.9135030139

 N61             4.3318570801            -3.4784082181            9.0490720944

 N62             4.4656884465            -8.0893984526            6.0361873626

 H63             3.5932804658            -8.6148309855            5.9551333116

 C64             5.2069915140#           -7.7515172429#           4.8093069829#

 C65             6.5295047429            -8.5197397048            4.8801875360

 O66             7.6352215189            -8.0529354881            4.6711331438

 H67             6.4161205008            -9.5742618605            5.1919308677

 H68             4.6287509288            -8.1859911514            3.9894832143

 C69             5.4022157030            -6.2408671890            4.5909136918

 H70             6.0000712662            -6.1147700262            3.6839342208

 H71             5.9876072940            -5.8319769946            5.4158921854

 C72             4.0731931164            -5.5004542573            4.4586829423

 H73             3.5750857128            -5.7582491675            3.5207607277

 H74             3.3837916598            -5.7912598345            5.2606113587

 C75             4.1348896340            -3.9835871154            4.5059900299

 O76             3.1689496540            -3.3967957595            3.9292488771

 O77             5.0787785588            -3.4001178533            5.1308028402

 C78             9.1595114482#            0.1191579465#          11.4478227400#

 H79             9.8386822634            -0.0710605331           10.6042069728

 H80             9.7330807813            -0.1473596969           12.3488764230

 C81             7.8877110632            -0.7393709048           11.3639428590

 H82             7.1471715467            -0.4270244607           12.1134838947

 H83             8.1360062888            -1.7774671702           11.6163613447

 C84             7.1271604115            -0.7942817799           10.0327836971

 O85             7.5426973544            -0.1164084068            9.0372844334

 O86             6.1102216181            -1.5472853358           10.0222110103

 C87             8.9453792448             1.6114620348           11.5090356311

 O88             7.8731663643             2.1820219067           11.5424834521

 H89             9.8806213135             2.2096545139           11.5488262082

 C90             7.7935564076#            4.3107436388#           8.7015790007#

 H91             8.1591985388             5.3211944430            8.9152527339

 H92             7.4141551481             3.8850073365            9.6326649972

 H93             8.6395721279             3.7010783432            8.3758090091

 C94             6.7056039632             4.3595041663            7.6256463164

 H95             5.8635813420             4.9882924742            7.9275964170

 H96             7.0999229484             4.7971281972            6.6975099456

 C97             6.1136606223             3.0088373606            7.2436320431

 O98             4.9733850969             2.9511552975            6.7475166955

 O99             6.8941087829             1.9894396190            7.4550331371

 C100           11.5816617657#           -0.6170833990#           3.4808992002#

 H101           12.2676131482            -0.9880698414            4.2521873819

 H102           12.0617794221             0.2379975757            2.9922380045

 H103           11.4545791851            -1.4073727125            2.7325237816

 C104           10.2292679877            -0.2179486159            4.0854022062

 H105            9.5719222811             0.1546047502            3.2906424674

 H106           10.3551064706             0.5957612290            4.8101671466

 C107            9.5476807768            -1.4134721962            4.7788959889

 H108           10.1720159758            -1.7462277746            5.6170528786

 H109            9.4563589323            -2.2473841660            4.0774845357

 C110            8.1711440221            -1.0899832849            5.3396886628

 O111            7.1622512739            -1.6022744921            4.7504713847

 O112            8.1201183913            -0.3480247450            6.3704031992

 C113           10.8187240943#            4.2285681070#          -0.8150212799#

 H114           11.5732906052             3.6127911381           -0.3125025596

 H115           11.1210651670             5.2776466311           -0.7174850131

 H116           10.8301864908             3.9721224500           -1.8790991553

 C117            9.4193987144             3.9982303484           -0.2110635716

 H118            9.1346206992             2.9495105758           -0.3810210704

 H119            8.6840707572             4.6062638568           -0.7566523446

 C120            9.3325229612             4.3347040409            1.2902113379

 H121            9.6832474043             5.3644726356            1.4418776328

 H122           10.0107192718             3.6873136363            1.8614279990

 C123            7.9241313868             4.2475004781            1.9091580546

 H124            7.2307080458             4.8967086519            1.3549780450

 H125            7.9534714085             4.6097244666            2.9408577758

 N126            7.4186151930             2.8739104276            1.9389359500

 H127            7.5568630475             2.3142099401            1.1079559469

 C128            6.6165278618             2.3152206341            2.8754917747

 N129            6.0266565251             3.0437093853            3.8282076130

 H130            5.5819158523             2.5782951449            4.6174293153

 H131            5.8641204566             4.0411673833            3.7393358063

 N132            6.4317346444             0.9972365204            2.8338335457

 H133            7.0674421588             0.3613237143            2.3468430200

 H134            5.6999145154             0.5452670252            3.3848894826

 C135           11.8423975518#           -5.3930895502#           7.2492549561#

 H136           11.3673114907            -6.3783689995            7.1940703456

 H137           12.9085660765            -5.5525194894            7.4393512703

 H138           11.7381407802            -4.9080047075            6.2731450651

 C139           11.2247931459            -4.5336660745            8.3632822558

 H140           11.7527226724            -3.5768789526            8.4416487222

 H141           11.3536428331            -5.0380795018            9.3307938885

 C142            9.7745657428            -4.2433085607            8.1353690078

 N143            8.8341565821            -5.2448327879            7.9301047917

 C144            9.0832670196            -3.0622536585            8.0624691593

 H145            9.4063599899            -2.0375552131            8.1545523414

 C146            7.6333572378            -4.6895235114            7.7354443036

 H147            6.7255084620            -5.2419181072            7.5458053513

 N148            7.7602316160            -3.3694607088            7.8146899036

 H149            6.9804566780            -2.6865828484            7.6209571925

 C150            6.0989354963#           -4.2351882770#          -1.2221974302#

 H151            5.5336281330            -4.7809767140           -1.9917242369

 C152            7.5233420621#           -4.0871570681#          -1.7388094266#

 H153            7.5953577612            -3.8186257297           -2.8149006840

 O154            8.5463715952            -4.2110124478           -1.0929406461

 H155            5.6661075967            -3.2242642799           -1.2260392382

 C156            5.9542137761            -4.8916842786            0.1410782862

 H157            6.2802523420            -5.9308601098            0.0967664452

 H158            6.5986669158            -4.3891821721            0.8705277233

 C159            4.5069649617            -4.8205254827            0.6303203252

 O160            3.9092009582            -3.7073319234            0.4941315247

 O161            3.9894195708            -5.8619319671            1.1282840930

 C162           -1.8288744975#          -11.9867165884#           4.2485682399#

 H163           -1.3963684401           -12.9439315688            3.9414225790

 H164           -2.8410917605           -12.1575687945            4.6373792463

 C165           -0.9849139174           -11.3796082541            5.3381809089

 H166           -1.4592343270           -10.5311075465            5.8796653726

 O167            0.1594673815           -11.7007157618            5.6005775325

 C168           -1.8853321611           -10.9934226028            3.0576315392

 H169           -2.6852003748           -11.3096750371            2.3771787030

 H170           -2.1856355113           -10.0058325030            3.4373706468

 C171           -0.5524954063           -10.9038002061            2.2980945348

 H172            0.2871782833           -10.7780279428            2.9896522830

 H173           -0.3730363010           -11.8672971019            1.8043134092

 C174           -0.4999453324            -9.7983805321            1.2305674532

 H175            0.4005042886            -9.9364751388            0.6195792645

 H176           -1.3515467870            -9.9057134392            0.5448224879

 C177           -0.5214496065            -8.3580164537            1.7487534263

 H178           -0.6177148671            -7.6636738792            0.9100180871

 H179           -1.3696118707            -8.1767355054            2.4146879394

 N180            0.7271697474            -7.9497204806            2.4811090824

 H181            1.0813952630            -7.0362710247            2.0277961206

 H182            0.5602276341            -7.7088183580            3.4693550213

 H183            1.4874477220            -8.6776186837            2.5002233729

 Cl184           0.7943743613            -6.7560473226            5.5527810943

 O185            0.9801615742            -1.8624508408            4.3377509131

 O186            4.6527144474            -1.1102762079            3.5978342710

 O187            0.9429974932             0.2945896611            6.5958925247

 H188            0.3135243304             0.3292887558            5.8444002261

 H189            1.0193533664            -0.6430840413            6.8428488762

 O190            2.3828878373             3.1525632899            7.9164444217

 H191            1.4379004798             3.3750393593            8.0353087464

 H192            2.8893595128             3.9870046332            7.9453041190

 O193            5.1050483429             1.6757587306           10.8692897854

 H194            5.0109560572             1.2514758654            9.9905230576

 H195            6.0550630331             1.8703628219           10.9633865508

 O196            2.3626764852            -1.9702381161            1.7440163624

 H197            2.7282120311            -2.7816090653            1.3091926255

 O198            3.1435529165            -1.5444052912            7.6628487880

 H199            1.9102377693            -1.4132831602            1.0779195172

 H200            0.6984106865            -2.7713334548            3.9961518453

 H201            0.2252683915            -1.2246553167            4.2863617697

 H202            8.9875130660            -6.2737430935            7.8073971525

 O203           -0.8467364253             0.9950156309            9.0289880775

 H204            0.0829431110             0.6813455795            9.0271249582

 H205           -0.7668457163             1.8839750203            8.6328779178

 O206           -0.9834050846             0.0056417419            4.6599466254

 H207           -1.5849079715            -0.0343860356            5.4582047560

 H208           -1.4110391718             0.6237930692            4.0318671545

 O209           -2.4575213231             0.3353172134            6.8876343091

 H210           -3.0137099388            -0.3936559876            7.2162904316

 H211           -1.8687395960             0.5551017678            7.6588301003

 O212            8.9938292890            -4.0204058704            1.7455954688

 H213            8.5579559981            -3.1424361056            1.8233624891

 H214            9.0297345264            -4.1706392412            0.7807461452

 O215            1.2942248926            -5.6497209282            1.3107257529

 H216            2.2803518706            -5.6079104518            1.1984301565

 H217            1.0662226571            -4.9880771598            2.0023021093

 O218            0.3307439271            -4.2645515364            3.5435955015

 H219            0.6542154220            -4.8624177673            4.2482023553

 H220           -0.6552416205            -4.3669301944            3.5469471245

 O221            5.2331683141            -1.3939029299            0.8726596237

 H222            4.7792418606            -2.2445672729            0.6161907858

 H223            4.9144440339            -1.2481931813            1.7945374155

 O224            7.6889069934            -1.5101896844            1.9922538118

 H225            6.8637578015            -1.5951952213            1.4457860895

 H226            7.3927276989            -1.6979187417            2.9097270111

 O227            8.7877810158            -7.9239720408            7.4021200344

 H228            7.8208340912            -7.9518168711            7.5643667095

 H229            8.8411739209            -8.0499974328            6.4365751195

 O230            2.5604706525            -9.9007831422            3.0510389941

 H231            2.4709281583            -9.9094879306            4.0282810440

 H232            3.5147770109           -10.0890173341            2.8577087133

 O233            5.7904445412            -7.9942990337            1.3948891802

 H234            5.0833095654            -7.3136001499            1.2986963804

 H235            6.5985493726            -7.4904479322            1.6551806500

 O236            7.9716617787            -6.5366495300            2.2619194848

 H237            8.1230807639            -6.8045317529            3.1825946686

 H238            8.2858148105            -5.6043051570            2.1712535303

 O239            2.0355613516            -9.6043275130            5.8209258187

 H240            1.4582787555            -8.8083134898            5.8053536903

 H241            1.4653579449           -10.3705959532            6.0348584365

 O242            5.2433983849           -10.2088148140            2.7190178468

 H243            5.5500155627           -10.9713240754            2.2055286073

 H244            5.4791233574            -9.4022098460            2.1678497939

 O245            5.0538150447            -0.0150253061           13.0826418275

 H246            4.9455801878             0.4479185085           12.2189967716

 H247            5.1747987473             0.7178515941           13.7048047913

 C248           -1.6084959362#            8.5839729289#           4.3490290518#

 C249           -2.3292207720             7.2374935351            4.1455712745

 C250           -1.9065103203             6.2064346035            5.1704495967

 C251           -2.3791124559             6.2847059679            6.4896474913

 C252           -0.9757208292             5.2011044641            4.8705513055

 C253           -1.9202379251             5.4236732309            7.4837894183

 C254           -0.4914231596             4.3344901116            5.8539670198

 C255           -0.9486708729             4.4597439518            7.1713726198

 O256           -0.3978434814             3.6615007295            8.1345971375

 H257           -1.8149965981             8.9901279729            5.3446678352

 H258           -2.1255072351             6.8634779896            3.1353411750

 H259           -3.4127217385             7.4006664615            4.2012222777

 H260           -3.1190519571             7.0403819620            6.7486649948

 H261           -0.6042179330             5.1051694667            3.8517029977

 H262           -2.2943051290             5.5032830990            8.4989401227

 H263            0.2723949020             3.5958714173            5.6289495856

 H264           -1.9320510665             9.3249147242            3.6087943172

 H265           -0.5234281046             8.4618542932            4.2557213224

 C266           -5.2856344204#           -5.5126297570#           5.1332787851#

 C267           -4.5361258557            -5.4477460573            3.7914940913

 C268           -3.0255458327            -5.4410192822            3.9637468065

 O269           -2.3583074768            -4.4673314494            3.5973725843

 N270           -2.4677106614            -6.5461927612            4.5254059759

 H271           -5.2041582713            -6.5036945513            5.5936496123

 H272           -4.7816535109            -4.5329903132            3.2481078647

 H273           -4.8290372876            -6.2945538638            3.1575961845

 H274           -3.0688498549            -7.2261795552            4.9704794171

 H275           -1.5050145770            -6.5144711241            4.8674312718

 H276           -6.3528086068            -5.3195574028            4.9865826247

 H277           -4.8993623859            -4.7870730538            5.8501194463

 C278           -4.9857218571#           -2.1244999399#           5.3205942263#

 C279           -5.5129689747            -1.4818091314            6.5773670593

 O280           -4.8467640341            -1.2136743580            7.5584401264

 H281           -4.0074415571            -2.5824404303            5.4730749067

 H282           -4.8847090600            -1.3404316104            4.5555976555

 H283           -6.5947104256            -1.2299566628            6.5685773582

 H284           -5.7088373131            -2.8454055446            4.9310503306

 C285           -1.2156620078#            3.5767179789#          14.9580159554#

 C286           -2.2300933959             4.2348452931           14.0089283497

 C287           -1.6288018494             4.5370193377           12.6745896306

 N288           -0.7686728496             5.6066118858           12.4866463577

 C289           -1.6679041805             3.8921396806           11.4635559196

 C290           -0.3320377747             5.5718659471           11.2076285088

 N291           -0.8604427721             4.5486885618           10.5577740430

 H292           -0.3102940576             4.1852149609           15.0514779892

 H293           -2.6194081694             5.1597788093           14.4558074955

 H294           -3.0909695882             3.5717935234           13.8686890706

 H295           -0.4643169292             6.2577511094           13.2141980579

 H296           -2.2014786768             2.9942412127           11.1861408373

 H297            0.3542346310             6.2956283020           10.7914439778

 H298           -0.5993556782             4.0310079043            9.0765279032

 H299           -1.6457924353             3.4482089351           15.9587748591

 H300           -0.9238852748             2.5935812665           14.5884451094

 C301           -0.2371729549#            6.7063619434#          17.0391968788#

 C302            0.6498193000             6.9644998553           15.8329907197

 O303            0.1803902787             7.1305428639           14.7048707393

 N304            1.9876077268             7.0362612161           16.0689563120

 H305           -1.0810515840             6.0889850867           16.7265707390

 H306            0.2797839753#            6.2148220261#          17.8670170309#

 H307            2.3840450205             6.9356607639           16.9916999112

 H308            2.6075052717             7.2469522330           15.2985257666

 H309           -0.6376458735             7.6619996681           17.3966368335

 C310           -1.7760929950#           -3.1799649726#           9.5045649029#

 C311           -2.2564994430            -3.5950498833            8.1108039074

 C312           -1.3910158139            -2.9623318059            7.0120159446

 C313           -2.2610413599            -5.1264329323            8.0007240570

 H314           -3.2879420669            -3.2396359975            7.9869551051

 H315           -1.4326848026            -1.8705134514            7.0733658383

 H316           -1.7170307148            -3.2615831118            6.0086032370

 H317           -0.3440849589            -3.2733779593            7.1158958522

 H318           -2.6104724235            -5.4558322960            7.0162009070

 H319           -2.9132721597            -5.5755404157            8.7605354754

 H320           -1.2516452020            -5.5348340586            8.1361583552

 H321           -2.0586670000#           -2.1742880127#           9.8440010378#

 H322           -0.6815890492            -3.2534668063            9.5698122148

 H323           -2.1831755020            -3.8630697360           10.2627872127

 C324            3.7583589157#            7.3762520312#           5.1844510197#

 C325            4.7656231127             6.9497046820            4.1410873315

 O326            5.2955619126             5.8493043708            4.0970628374

 C327            2.6536442256             6.3562479684            5.5214461866

 C328            1.8718838313             6.8044840178            6.7684794295

 C329            2.7206593439             6.7372522151            8.0329743230

 O330            3.4386082458             5.7729206378            8.3002651955

 N331            2.6350504204             7.8019553879            8.8772201726

 H332            4.2986990520#            7.5050089703#           6.1328509745#

 H333            3.1069551884             5.3793315319            5.7143769651

 H334            1.9538103332             6.2400294634            4.6879049527

 H335            1.0199993064             6.1340618837            6.9167034349

 H336            1.4556812454             7.8095153389            6.6274833304

 H337            2.1037733485             8.6301706289            8.6548558698

 H338            3.1942910955             7.7921410652            9.7206536073

 H339            5.0370394810             7.7273665118            3.3842214662

 H340            3.3418371425             8.3523155335            4.9078641114
